# Potential Targets for CRISPR/Cas Knockdowns to Enhance Genetic Resistance Against Some Diseases in Wheat (*Triticum aestivum L.*)

**DOI:** 10.3389/fgene.2022.926955

**Published:** 2022-06-22

**Authors:** Mehwish Taj, Muhammad Sajjad, Mingju Li, Arooj Yasmeen, Muhammad Salman Mubarik, Sirisha Kaniganti, Chi He

**Affiliations:** ^1^ Department of Biosciences, COMSATS University, Islamabad, Pakistan; ^2^ Yunnan Key Laboratory of Green Prevention and Control of Agricultural Transboundary Pests, Agricultural Environment and Resource Institute, Yunnan Academy of Agricultural Sciences, Kunming, China; ^3^ Department of Biotechnology, University of Narowal, Narowal, Pakistan; ^4^ International Crops Research Institute for the Semi-Arid Tropics, Patancheru, India

**Keywords:** S genes, crispr/cas, stripe rust, leaf rust, powdery mildew, biotic stress, wheat, genetic resources

## Abstract

Wheat is one of the most important food crops worldwide. Even though wheat yields have increased considerably in recent years, future wheat production is predicted to face enormous challenges due to global climate change and new versions of diseases. CRISPR/Cas technology is a clean gene technology and can be efficiently used to target genes prone to biotic stress in wheat genome. Herein, the published research papers reporting the genetic factors corresponding to stripe rust, leaf rust, stem rust, powdery mildew, fusarium head blight and some insect pests were critically reviewed to identify negative genetic factors (Susceptible genes) in bread wheat. Out of all reported genetic factors related to these disease, 33 genetic factors (S genes) were found as negative regulators implying that their down-regulation, deletion or silencing improved disease tolerance/resistance. The results of the published studies provided the concept of proof that these 33 genetic factors are potential targets for CRISPR/Cas knockdowns to improve genetic tolerance/resistance against these diseases in wheat. The sequences of the 33 genes were retrieved and re-mapped on the latest wheat reference genome IWGSC RefSeq v2.1. Phylogenetic analysis revealed that pathogens causing the same type of disease had some common conserved motifs and were closely related. Considering the significance of these disease on wheat yield, the S genes identified in this study are suggested to be disrupted using CRISPR/Cas system in wheat. The knockdown mutants of these S genes will add to genetic resources for improving biotic stress resistance in wheat crop.

## Introduction

Wheat (*Triticum aestivum* L.) is grown in 89 countries and consumed by 2.5 billion people due to its dietary values (CGIAR, 2019). Each growing season, wheat is exposed to a wide range of diseases and pests that affect the crop yield (Gulnaz et al., 2019). Among biotic stresses, pathogenic fungi pose a serious threat to global wheat production. Stripe rust, stem rust, leaf rust, powdery mildew, and head blight are the primary diseases of wheat (Simón et al., 2021). Stripe rust has historically caused and continues to cause catastrophic losses in sensitive wheat cultivars globally (Gad et al., 2019). According to a recent estimate, 21.5% wheat yield losses are due to pests and diseases (Savary et al., 2019). Thus far, various breeding methods and biotechnological tools have exploited resistant genes (R genes) to breed biotic stress tolerant wheat varieties over different periods of time (Supplementary Figure S1). However, susceptible genes (S genes) are not yet explored to improve resistance against pests and diseases.

The advent of CRISPR/Cas (clustered regularly interspaced short palindromic repeats-CRISPR-associated) system such as CRISPR/Cas9 and CRISPR/Cas12 for precise genome editing presents great scope of targeting S genes to improve economical traits in crops including wheat (Mubarik et al., 2021). As an allohexaploid, however, wheat has three closely linked sub genomes that were passed down from three homoeologous ancestors, with 2n = 6x = 42, AABBDD (Petersen et al., 2006). The A, B, and D genomes each contain three copies of a gene that is functionally redundant and complementary. As a result, it is extremely unlikely that natural selection or induced mutagenesis will result in the simultaneous mutation of genes in the human genomes A, B, and D. Consequently, compared to other cereals like rice and maize, wheat’s complicated polyploid structure has hampered the development of functional genomics and breeding (Char et al., 2017), and the failure to eliminate all of a gene’s duplicates may not necessarily result in phenotypic changes due to genome buffering. On the other hand, wheat’s genome is massive (∼17 Gb) and contains a high proportion of repetitive DNA (80%–90%), making targeted modifications extremely difficult. However, with the availability of novel Cas orthologues, gRNA design in the CRISPR/Cas systems has grown more flexible and can be easily created to target a variety of genes (Char and Yang 2020; Gürel et al., 2020).

To date, Cas9 and Cas12a, have been used for genome editing in wheat to create new alleles and disrupt gene’s function (Kefale & Getahun 2022). Due to their unique pros and cons, Cas9 and Cas12a have made the applications of CRISPR/Cas system highly versatile. Cas12a, has certain advantages over Cas9, in its ability to be used for multiplex genome editing and production of staggered DSB (double-stranded break), which promotes HDR (homology-directed repair) instead of NHEJ (non-homologous end joining).

Continuous improvement in genetic resources for biotic stress resistance is pre-requisite for sustained increase in yield potential of newly developing wheat varieties (Alemu, 2019). This report intends to provide a guide for exploiting S genes through CRISPR/Cas knockdowns to develop new genetic resources for breeding biotic stress resistant wheat varieties. The genetically stable knockdown-mutants the S genes could provide new genetic resources for enhancing biotic stress tolerance in future wheat varieties.

## Resistance (R) Versus Susceptible (S) Genes

Plants have evolved a sophisticated immune system through co-evolution with diseases, while pathogens have developed counter-defense mechanisms. The pathogen-associated molecular patterns (PAMPs) such as bacterial flagellin or viral double-stranded RNAs are detected by PRRs on the cell surface, activating PAMP-triggered immunity (PTI). PTI causes dynamic changes in the defense-responsive transcriptome, reactive oxygen species (ROS) generation, and antimicrobial peptide/compound release in the apoplast (Jighly et al., 2016). Pathogens release virulence proteins or effectors and other poisons to reduce PTI (Huerta-Espino et al., 2011). These effector chemicals also change plant physiology to aid infection. Plants have evolved R genes that may detect effector activities and trigger effector triggered immunity (ETI). Strong defensive responses often cause localized cell death or hypersensitivity (Sharma et al., 2015; Alemu, 2019). S genes, on the other hand, are required for pathogen infection and consequently for suitable plant–pathogen interactions. They help in host identification and penetration, pathogen growth and spread, and negative modulation of immunological signals (Zaidi et al., 2018). While R genes are dominant, disease resistance offered by S genes is recessive and comes with a fitness penalty. S-gene-mediated disease resistance is pathogen specific when the damaged pathway is required for pathogen entry, penetration, or post-penetration. A suitable host surface state is essential for bacterial adhesion or fungal/oomycete spore germination prior to penetration. Pathogens enter their hosts in a variety of ways. Direct penetration through physical or chemical barriers, and indirect penetration through natural cell openings like stomata. Pathogens invade host cells after penetration by avoiding plant monitoring systems and/or dampening numerous levels of defense (Zaidi et al., 2018). In the same way, the target S gene involved in protracted or constitutive defense responses can be broad-spectrum. We use genome editing to target S-gene-mediated pathogen resistance.

## Selection of Target S Genes and Their Functions in Wheat

The target S genes were selected based on critical review of research papers published from well-known labs. The genes/genetic factors whose absence, down regulation, reduced expression, silencing or loss of function mutation improved resistance against one or more than one of the diseases or insect pests were considered as S genes. Out of >100 genes reported to be associated with stripe rust, leaf rust, stem rust, powdery mildew and fusarium head blight, 33 were selected as S genes with strong concept of proof (Supplementary Table S1). The R genes or the genes without any functional concept of proof were not included in this mini review. Herein, 20 target S genes were selected for stripe rust. For powdery mildew, fusarium head blight, aphides and leaf rust, 4, 7, 1 and 1, target S genes were selected, respectively. Besides improving resistance to disease, the knockdowns of these S genes could cause some yield penalty or negatively affect some other agronomic traits. For example, the knockdown of *TaNAC21/22* gene produces necrotic and shorter leaves which can lead significant yield penalty. The side effects of knockdowns of the S genes can be recovered by subsequent backcrossing with wild plants. The CRISPR/Cas knockdowns of these genes to develop null mutants will create valuable genetic resources for breeding against disease in wheat. Further, the orthologs of the selected S genes from Zea *mays*, *Oryza sativa*, were also searched to confirm their similar functions in maize and rice using NCBI-BLASTp (https://blast.ncbi.nlm.nih.gov) and phytozyme database (https://phytozome-next.jgi.doe.gov/). Based on their homology, complete sequence information of the selected 33 S genes were extracted from NCBI.

## Mapping of Selected S Genes

**FIGURE 1:**
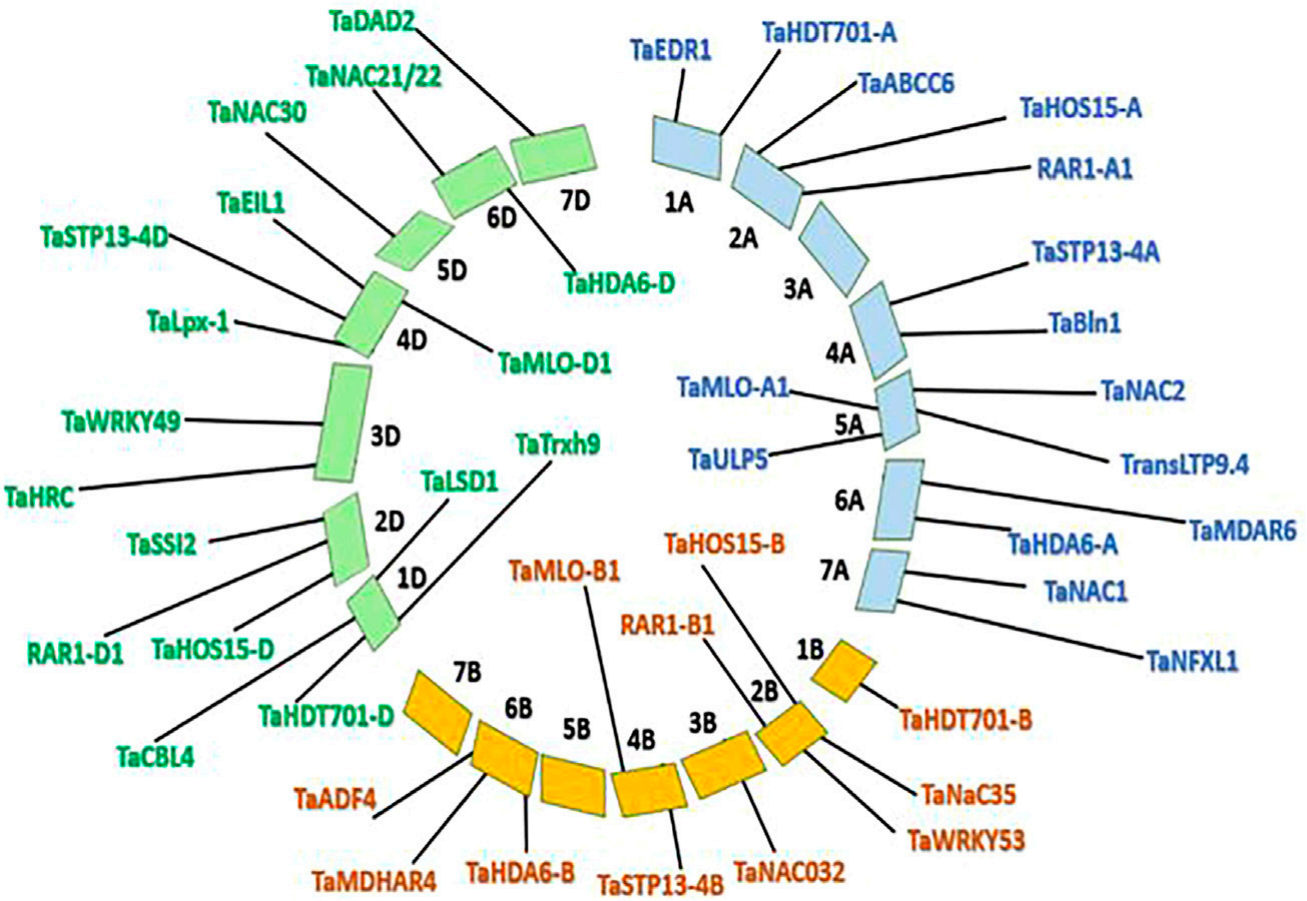
Mapping of selected S genes on wheat genome using URGI tool.

To map the selected S genes Wheat URGI (https://wheat-urgi.versailles. inrae. fr) (Appels et al., 2018) database was used. All the retrieved sequences were matched using public blast. Once the alignment was retrieved, chromosomes to be mapped were selected using IWGSC RefSeq v2.1. The map was constructed based on the highest similarity score and lowest *E*-values (Figure 1).

## Multiple Sequence Alignment and Phylogenetic Analysis of the Selected S Genes

For multiple sequence alignment (MSA) of the selected S genes, Clustal W software (Thompson et al., 1994) was used (Supplementary Figure S2). The Molecular Evolutionary Genetics Analysis (MEGA) program is a computer tool that lets you to compare homologous genetic sequences from many classes or multi - gene families, with a focus on implying evolutionary links and patterns of DNA and protein evolution (Kumar et al., 2018). For phylogenetic analysis of the S genes MEGA software (Kumar et al., 2018) was used (Supplementary Figure S3).

All the coding sequences were retrieved from NCBI database and homology between the negative regulator genes was identified. Based on the phylogenetic analysis pathogens causing strip stripe rust, powdery mildew, leaf rust, and fusarium head blight were predicted to have common conserved motifs and to be closely related from evolution perspective.

## Future Perspectives

The CRISPR/Cas9 genome-editing technique has made a big splash in plant genetics. It is the most sophisticated gene - editing system ever built due to its remarkable versatility in attacking any DNA sequence with the greatest specificity and modification effectiveness (100%). Furthermore, unlike typical transgene-carrying GMOs, CRISPR/Cas9 doesn’t really incorporate foreign genes into the plant genome, so genetically altered plants are not (yet) subject to legal constraints. Since its first use in plants 4 years ago, this approach has proven to be an innovative tool for increasing critical mating targets including yield, quality, herbicide tolerance, and biotic/abiotic stress resistance. This method is also utilized to change the patterns and architecture of plant inflorescences, as well as to manipulate gene expression through transcriptional regulation.

CRISPR-based genome editing and CRISPR, when combined with other breakthroughs such as the better transgenic technologies and generation of high-quality genome sequences, would propel rational design-based molecular breeding of polyploid wheat and functional genomics to the forefront of wheat biology. Gene-edited wheat and transgene-free, we believe, will play a crucial role in addressing environmental challenges while boosting maintainable agriculture. This is important to note that it is not a substitute for conventional breeding; rather, it is one of the strategies for speeding up wheat biology and developing wheat breeding programs.

## Conclusion

Herein, 33 S genes were selected as potential targets for CRISPR/Cas9 knockdowns. The genetically stable knockdown mutants can be used as valuable parents for designing crosses to breed disease resistant cultivars in wheat. The MSA and phylogenic analysis of the selected genes revealed that the S genes related to a specific disease such as stripe rust share some common conserved motifs and have high sequence similarities. Using this information new S genes could be identified using relevant bioinformatics tools and validated in wet lab experiments.
