# Supplementary material for: Potential Targets for CRISPR/Cas Knockdowns to Enhance Genetic Resistance Against Some Diseases in Wheat (Triticum aestivum L.)
Source: Front Genet. 2022 Jun 22;13:926955. doi: 10.3389/fgene.2022.926955 (PMC9245383; doi:10.3389/fgene.2022.926955)
Supplement: Supplementary file 1 [file Table1.DOCX]

Table 1: The selected S genes, their functions and associated wheat disease (s).

|  |  |  |  |  |  |  |
| --- | --- | --- | --- | --- | --- | --- |
| Sr No | **Gene Name** | **Function** | **Positive Effects** | **Negative Effects** | **Disease/Insect pest** | **References** |
| 1. | *TaNAC2* | Highly induced in wheat-Puccinia striiformis (Pst) interactions and ABA treatment. | *TaNAC2* silencing increased tolerance to Pst by dramatically increasing H2O2 production and preventing hyphal development during the initial interaction phase. | TaNAC2 knockdown had no effect on Suwon 11's susceptibility phenotype to Pst during Pst infection. | **Stripe rust** | (X. Zhang et al., 2018) |
| 2. | *TaRop10* and *TaTrxh9* | *TaRop10*, a small GTP-binding protein, comes in contact with *TaTrxh9* and behaves like a negative regulator of wheat tolerance to stripe rust ] | Silencing of *TaRop10* and *TaTrxh9* in wheat resulted in enhanced resistance. | On the leaves of BSMV: *TaTrxh9*, we detected the onset of modest chlorotic mosaic symptoms at 10 dpi. |  | (Shi, Wang, Gao, Yang, Wang, Day, et al., 2021) |
|  |  |  |  | When inoculated with CYR31, the leaves of *TaTrxh9*-silenced plants displayed increased urediospore. |  |  |
| 3. | *TaNAC30* | *TaNAC30* has transcriptional activity, and transcription activation requires its C-terminus. | The virus-induced gene silencing of TaNAC30 inhibited colonization of infectious Pst isolate CYR31. | At 48 and 120 hpi, the region comprising H2O2 was considerably bigger in TaNAC30-knockdown seedlings compared to control plants. |  | (B. Wang et al., 2018) |
|  |  | TaNAC30 expression increased once host plant got infected with a virulent strain (CYR31) of the rust fungus Pst. | Furthermore, comprehensive histological analyses revealed that suppressing TaNAC30 increased tolerance to Pst by causing a substantial increase in H2O2 concentration. |  |  |  |
| 4. | TaNAC1 | It's a transcription factor with an activation domain at its C-terminus that's found in the nucleus of cells. TaNAC1 was found in wheat roots system and was involved in defense-related hormonal therapies like salicylic acid (SA), ethylene, and methyl jasmonate as well as responses to the obligate pathogen *Puccinia striiformis* f. sp. tritici. | TaNAC1 silencing with barley stripe mosaic virus-induced gene silencing (BSMV-VIGS) improved stripe rust resistance. |  |  | (F. Wang et al., 2015) |
| 5. | TaNAC21/22 | TaNAC21/22 is found in the nucleus and function as a transcriptional activator. | The control of NAC21/22 by miR164 impacts stripe rust disease resistance in wheat. In response to Puccinia striiformis f. sp. tritici, transcript accumulation of TaNAC21/22 and tae-miR164 displayed different divergent expression patterns (Pst). | The necrotic region accumulations were substantially higher in knocked down leaves of TaNAC21/22. |  | (H. A. O. Feng et al., 2014) |
|  |  | TaNAC21/22 is found to be a negative regulator of stripe rust resistance when the gene is silenced. |  | Pst hyphal length was considerably shorter in the knocked-down leaves. |  |  |
|  |  |  |  | Knocked down wheat leaves of TaNAC21/22 were revealed to be substantially shorter. |  |  |
| 6. | TaSTP13 | TaSTP13 is transcriptionally activated that leads toward wheat susceptibility to Pst by boosting the buildup of cytoplasmic hexose for fungal sugar uptake in wheat–Pst interactions. | Wheat susceptibility to Pst was lowered when TaSTP13 was knocked out using barley stripe mosaic virus-induced gene silencing (VIGS). | In TaSTP13 knockdown plants, fungal hypha was noticeably broader and showed abnormal swelling structures. |  | (Huai et al., 2020; Jamil et al., 2020) |
| 7. | TaWRKY49 | The WRKY gene TaWRKY49 was first recognized in wheat in association with high-temperature seedling-plant tolerance to Pst (HTSP). Silencing of TaWRKY49 enhanced HTSP resistance. | TaWRKY49 silencing increased disease resistance in wheat plants compared to non-silenced plants. TaWRKY49 silencing resulted in increased resistance, which was linked to genes responsive to salicylic acid and jasmonic acid, TaAOS and TaPR1.1, as well as the ROS-related genes TaPOD and TaCAT, whose expression levels were all repressed. | - |  | (Tolerance & Improvement, n.d.; J. Wang et al., 2017) |
| 8 | TaDAD2 | TaDAD2 is engaged in defense response to the stripe rust fungus as well as the inhibition of plant cell death in wheat. | TaDAD2 expression was reduced by VIGS, which increased wheat susceptibility. TaDAD2 knockdown reduced fungal-induced cell death and enabled for limited fungal growth and uredium development, equating to a lower infection type of the wheat stripe rust fungus . |  |  | (Miché et al., 2018) |
| 9, | TaLSD1 | The nucleus of onion epidermal cells was revealed to be home to TaLSD1. TaLSD1 serves a negative effect in modulating hypersensitive cell death in plants and is engaged in tolerance to disease of wheat against the pathogen stripe rust. | Enhanced wheat tolerance to Pst was associated by an improved hypersensitive response (HR), decrease in Pst hyphal development, and rise in PR1 gene expression when the expression of TaLSD1 was knocked down using VIGS. Bax-induced PCD is partially suppressed by TaLSD1. | At 48 and 120 hpi, in TaLSD1 knockdown plants, hypersensitive cell death induced by infection with avirulent Pst race CYR23 became much more comprehensive, and the necrotic area per infected area was bigger than the control. Increased ROS production during the initial phases of avirulent Pst race intrusion could stimulate the functional impairment of TaLSD1 at the HR margin, transmitting cell death signals to uninfected sites. |  | (J. Guo et al., 2013) |
| 10 | TaMDHAR4 | TaMDHAR4 which is cytoplasmic protein is evaluated as a peroxisomal protein and controlled by miRNA PN-2013. | The mono dehydroascorbate reductase gene, TaMDHAR4, has been illustrated to assist wheat stripe rust infection. The depletion of the hyphae growth of the biotrophic pathogen *Puccinia striiformis* is caused by its mutation. So blocks its sporulation and increasing necrosis at the infection site. | The necrotic area of TaMDHAR4 Knocked down plants was more acute in the wheat leaf surface site on its phenotype, and this observation is compatible with the results of the histological observations. The necrotic area of the host would be expressed by the content of H2O2 in the host cells. |  | (Abou-Attia et al., 2016; Fabre et al., 2020; H. Feng et al., 2014) |
|  |  |  |  |  |  |  |
| 11 | TaDIR1-2 | TaDIR1-2 is involved in the negative regulation of wheat resistance opposed to Puccinia striiformis f. sp. Tritici. | Knocking down the expression of TaDIR1-2 through virus-induced gene silencing enhanced wheat resistance assisted by HR. | H2O2 cumulated in TaDIR1-2 knockdown plants to the size over 2.5- and 1.5-fold higher than in the control plants, and necrotic cell death show particularly enhanced pattern respectively. |  | (Ahmed et al., 2017) |
| 12 | TaULP5 | TaULP5 confers to the consistent association of adult plant resistance wheat seedlings stripe rust pathogen. | Knockdown of TaULP5 enhance the expression levels of some biotic stress-related genes, such as PR1 and PR2, also increased the resistance at the seedling stage of wheat. | In TaULP5-silenced plants the enormous necrotic areas around the Pst infection sites were observed at 48 and 120 hpi . |  | (H. Feng et al., 2016) |
| 13 | TaCBL4 | The calcium detector TaCBL4 and its interconnecting protein TaCIPK5 are needed for wheat resistance to stripe rust fungus. | Silencing of TaCBL4 prompted in increased susceptibility to avirulent Pst infection. | The contagious area was particularly enhanced in TaCBL4-knockdown plants through suited and unsuited relations contrast to the control. |  | (Liu et al., 2018) |
| 14 | TaNUDX23 | TaNUDX23 is a negative regulator of resistance. | Knocking down the expression of TaNUDX23 diminished Pst infection, implied that TaNUDX23 is a negative regulator of resistance. | Wheat leaves immunized with the TaNUDX23-1/2 as silencing makes and CYR31, sporulation was decreased in contrast to the control plant. |  | (Yang et al., 2020) |
| 15 | TaMDAR6 | TaMDAR6 is a negative regulator of plant cell death and assists by implication in stomatal regulation during the wheat stripe rust–fungus interaction. | Silencing of TaMDAR6 increases the resistance of wheat. | H2O2 assembly at the interaction sites slowly enhanced in the TaMDAR6 knocked down plants at 24 and 48 hpi. Also, hypersensitive cell death increased in the TaMDAR6 knocked-down plants at 48 hpi. |  | (Abou-Attia et al., 2016) |
|  |  |  |  |  |  |  |
| 16 | TaEIL1 | TaEIL1, a wheat homologue of AtEIN3, acts as a negative regulator in the wheat–stripe rust fungus interaction. | Knocking down TaEIL1 through the Barley stripe mosaic virus (BSMV) virus-induced gene silencing (VIGS) system attenuated the growth of *Pst*, would increase the wheat resistance to stripe rust fungus. |  |  | (Duan et al., 2013) |
| 17 | TaBln1 | TaBln1 negatively regulates wheat resistance to stripe rust by decreasing Ca2+. | The Knockdown of TaBln1 by virus-induced gene silencing decreased Pst manipulation and evolution and increase the host defense response. | The accumulation of H2O2, number of hyphal branches, infection area, and haustorial mother cells, and haustoria were detected in TaBln1 knockdown plants. |  | (S. Guo et al., 2021) |
|  |  |  |  |  |  |  |
| 18 | TaADF4 | TaADF4, an actin depolymerizing factor from wheat, is required for resistance to the stripe rust pathogen Puccinia striiformis f. sp. Tritici. | Knockdown of TaADF4 induced in increased susceptibility to CYR23, indicating a role for TaADF4 in defense signaling. | TaADF4 knockdown plants had decreased levels of Jasmonic acid. Salicylic acid levels in TaADF4-knockdown plants reduced by approximately 17% at 18 hpi. |  | (B. Zhang et al., 2017) |
| 19 | TaATG8j | TaATG8j plays a role in wheat resistance towards stripe rust fungus by modulating cell death, giving knowledge for the understanding of the techniques of wheat resistance to the stripe rust pathogen. | The virus-induced gene silencing of TaATG8j duplicates provide Suwon 11 susceptible to the avirulent Pst race CYR23, assisted by an enhanced fungal biomass and a reduced necrotic area per infection site. | HR was detected on the fourth leaves of the BSMV: TaATG8j-knockdown wheat seedlings. Sporadic fungal sporulation was detected around the necrotic spots at 18 dpi. |  | (Mamun et al., 2018) |
| 20 | TaRAR1 | TaRar1 is included in wheat defense against the pathogen stripe rust, which is moderated by YrSu . | When the stripe rust resistance gene YrSu was silenced in wheat cultivar Suwon11, the endogenous salicylic acid (SA) level, hydrogen peroxide (H2O2) aggregation, and the level of hypersensitive response (HR) were all reduced, and resistance to the avirulent pathotype of stripe rust was balanced. | After immunization with CYR23, in the TaRar1 silenced leaves the fungal hyphae were particularly extended. |  | (X. Wang et al., 2017) |
|  |  |  |  | The infection area was remarkably enormous in the TaRar1-silenced plants at 120 hpi as compared to the non-silenced. |  |  |
|  |  |  |  | H2O2 accumulation was particularly decreased in the TaRar1 silenced leaves in contrast to the control. |  |  |
| 21 | TaMLO | The loss of function provides resistance to powdery mildew. According to one study, MLO genes might be accountable for responding to powdery mildew infection, and inhibiting mlo genes repressed powdery mildew diseases in barley. | The CRISPR TaMLO knockout shows resistance to powdery mildew disease caused by *Blumeria graminis* f. sp. Tritici (Btg). | - | **Powdery mildew** | (Jaganathan, Ramasamy, Sellamuthu, et al., 2018) |
|  |  |  | Powdery mildew resistance was achieved by using particle bombardment to target the TaMLO-D1, TaMLO-A1, and TaMLO-B1 genes with the CRISPR/Cas9 and TALEN technologies. |  |  |  |
| 22 | TaEDR1 | The wheat Enhanced disease resistance 1 (TaEDR1) gene is a negative regulator of powdery mildew resistance. | CRISPR/Cas9 is used to knock out EDR1, which has detrimental role in the resistance mechanisms against powdery mildew, resulting in wheat plants with enhanced powdery mildew resistance. |  |  | (Zaidi et al., 2018) |
| 23 | TaHDA6, TaHDT701, TaHOS15 | TaHDT701 is associated with RPD3 gene histone deacetylase TaHDA6 and WD40-repeat protein TaHOS15 to form a histone deacetylase complex, in which TaHDT701 can stabilize the TaHDA6-TaHOS15 association. Chromatin immunoprecipitation tests have shown that TaHDT701 can work in partnership with TaHOS15 to hire TaHDA6 promoters of immune-related genes such as TaPR1, TaPR2, TaPR5, and TaWRKY45*.* | TaHDA6 represses histone acetylation at promoters of defense-related genes and thus, negatively regulates their expressions as well as plant defense responses to Bgt . | The interrelation of TaHDA6 along with TaHOS15 was decreased in TaHDT701-silenced plants in contrast by the control, showing that the TaHDT701 balance the TaHDA6-TaHOS15 link in the bread wheat. The expression levels of TaPR2, TaWRK45, TaPR5 and TaPR1 and distribution of histone H4K16Ac were influenced by knockout of TaHDT701, TaHDA6, and TaHOS15. |  | (Kong et al., 2020; Zhi et al., 2020) |
|  |  |  | Silencing of TaHDT701, TaHDA6, and TaHOS15 compromises wheat susceptibility to Bgt. The elimination of TaHDT701, TaHDA6, and TaHOS15 has resulted in a strong resistance to wheat powdery mildew, implying that the complex of TaHDT701-TaHDA6-TaHOS15 histone deacetylase negatively regulates wheat immune responses to Bgt . |  |  |  |
| 24 | TaHDT701 | Histone Deacetylase TaHDT701 Functions in TaHDA6-TaHOS15 Complex to Regulate Wheat Defense Responses to Blumeria graminis f.sp. tritici. | Silencing of TaHDT701, TaHDA6, and TaHOS15 evolved in increased powdery mildew resistance of wheat, intimating that complex of TaHDT701-TaHDA6-TaHOS15 histone deacetylase negatively regulates wheat immune reactions to Bgt. | MNase digestion tests displayed diminished nucleosome habitation at the alike chromatin areas in the TaHDT701, TaHDA6, and TaHOS15-silenced plants. The interaction in between TaHDA6 and TaHOS15 was noticeably decreased in the TaHDT701-silenced plants compared with the control. |  | (Zhi et al., 2020) |
| 25 | Ta_PYL4AS_A | A virus-induced gene silencing indicate a function of the wheat ABA receptor Ta_PYL4AS_A, and its adjacent homologs, interpose FHB susceptibility and in reducing mycotoxin accumulation. | Virus induced gene silencing (VIGS) technique was used to knockdown wheat Ta_PYL4AS_A and its relevant plants, yielding plants with enhanced early-stage resistance to FHB development and reduced mycotoxin accumulation. | - | **Fusarium head blight** | (Fabre et al., 2020; Gordon et al., 2016) |
| 26 | TaSSI2 | As a stearoyl-acyl carrier protein fatty acid desaturase, TaSSI2 might be involved in cell lipid metabolism and carry lipids targets out of the cell for membrane or wax synthesis. A TaSSI2 gene in wheat was proved to be involved in multiple biological functions including FHB, powdery mildew resistance and drought tolerance. | The silencing of the wheat TaSSI2 gene enhance FHB resistance, stimulating salicylic acid signaling and potentially changing the jasmonic acid pathway as illustrated in Arabidopsis ssi2 mutant lines. |  |  | (Fabre et al., 2020; HU et al., 2018) |
| 27 | TaNAC032 | TaNAC032 play a role as transcription component in regulating the FHB resistance in wheat. TaNAC032 plays a role as main regulator of genes connected to the biosynthetic lignin process. | TaNAC032 gene was silenced by using virus- induced gene silencing (VIGS) in NIL-R to show the resistance roles in wheat NILs that are opposed to FHB. | The corresponding expression of gene levels of downstream RRRM genes were particularly lower in Tanac032 silenced plants in contrast to control samples. |  | (Soni et al., 2021) |
| 28 | TaTIR1 | The auxin receptor gene TaTIR1 is included in plant resistance to biotic stresses. | TaTIR1 knockdown spikes indicated particularly enhance resistance to FHB. | In TaTIR1 knockdown plants the transcript levels of various genes that includes AUX/IAA, GH3, AUX, SAUR and ARF were particularly reduced in contrast to WT. |  | (P. Su et al., 2021) |
| 29 | TaLpx-1 | Resistance to *Fusarium graminearum* is provided by lipoxygenase. | TaLpx-1, which encodes a 9-LOX with homology to LOX1 and LOX5, was silenced, resulting in increased resistance to *F. graminearum* in wheat. | - |  | (Jaganathan, Ramasamy, & Sellamuthu, 2018; Nalam et al., 2015) |
| 30 | TaHRC | TaHRC, a gene that encodes a putative histidine-rich calcium-binding protein, is a critical determinant of Fhb1-mediated FHB resistance. | TaHRC generates a nuclear protein that confers FHB susceptibility, and removal spanning this gene's start codon results in FHB resistance. Fhb resistance (knockout). |  |  | (Li et al., 2021; Z. Su et al., 2019) |
|  |  |  | TaHRC is preserved gene in cereal grains and knocking out TaHRC-S expression substantially enhanced FHB resistance, opening up innovative opportunity for improving FHB resistance in wheat and possibly other cereal crops through biotechnology manipulation of TaHRC-S sequence. |  |  |  |
| 31 | TaABCC6, TaNFXL1, TansLTP9.4 | TansLTP9.4 is a gene that encodes a nonspecific lipid-transfer protein (nsLTP), as well as an ABC transporter (TaABCC6), the nuclear transcription factor X box-binding-like1 (TaNFXL1). | Wheat resistance to Fusarium head blight (FHB) was established through aiming three wheat genes: (TaABCC6), (TaNFXL1), and (nsLTP), TansLTP9.4. All 3 genes were determined to be important in FHB defense and were altered with a 42.2 percent mutation efficiency. |  |  | (Zaman et al., 2018) |
| 32 | TaWRKY53 | The WRKY genes belong to a large family of plant-specific transcription factors that play specific role in growth and resistance to biotic and abiotic stresses. The roles of WRKY53 and PAL in aphid tolerance remain unknown. | TaWRKY53 silencing in wheat has validated its function in aphid defense. | Abrogating WKRY53 function had greater negative impact on GR plant antibiotic capacity than knocking out PAL, and it also resulted in PAL transcript reduction. This suggests that WRKY53 possibly regulates expression of wide range of defense-related genes, including PAL and the phenylpropanoid pathway. | **Aphids** | (Kage et al., 2017; Van Eck et al., 2010) |
| 33 | TaNAC35 | When exposed to a virulent isolate of Puccinia triticina (Pt), the TaNAC35 gene inhibits leaf rust resistance in the wheat line Lr14b + Thatcher (TcLr14b). | TaNAC35 inhibition limited the forming of haustorial mother cells (HMC) and mycelial growth, indicating that the TaNAC35 gene plays a negative role in TcLr14b's response to Pt pathotype THTT. | Knockdown of TaNAC35 by VIGS increased resistance to wheat leaf rust pathogen but also caused HR in near isogenic line TcLr14b. | **Leaf rust** | (N. Zhang et al., 2020) |
