# Supplementary material for: Potential Targets for CRISPR/Cas Knockdowns to Enhance Genetic Resistance Against Some Diseases in Wheat (Triticum aestivum L.)
Source: Front Genet. 2022 Jun 22;13:926955. doi: 10.3389/fgene.2022.926955 (PMC9245383; doi:10.3389/fgene.2022.926955)
Supplement: Supplementary file 2 [file DataSheet2.DOCX]

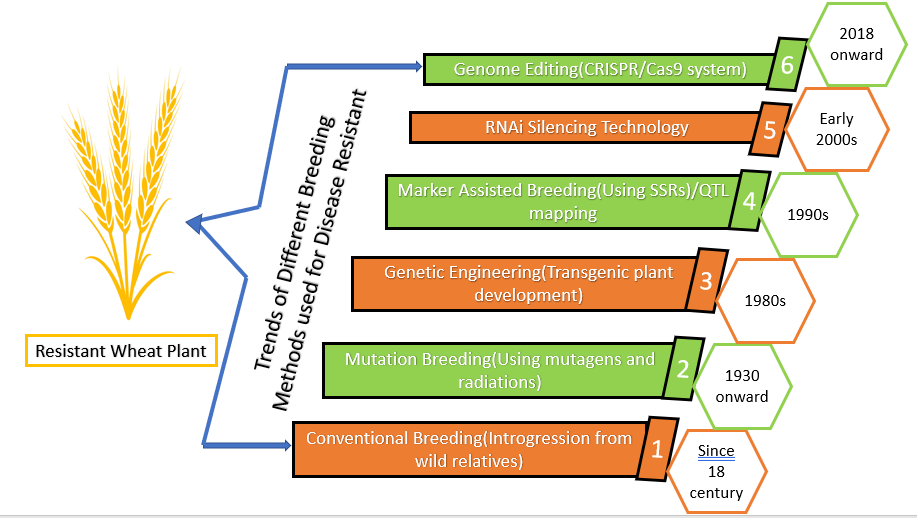


Figure S1. Trends of breeding methods for improving disease resistance in wheat


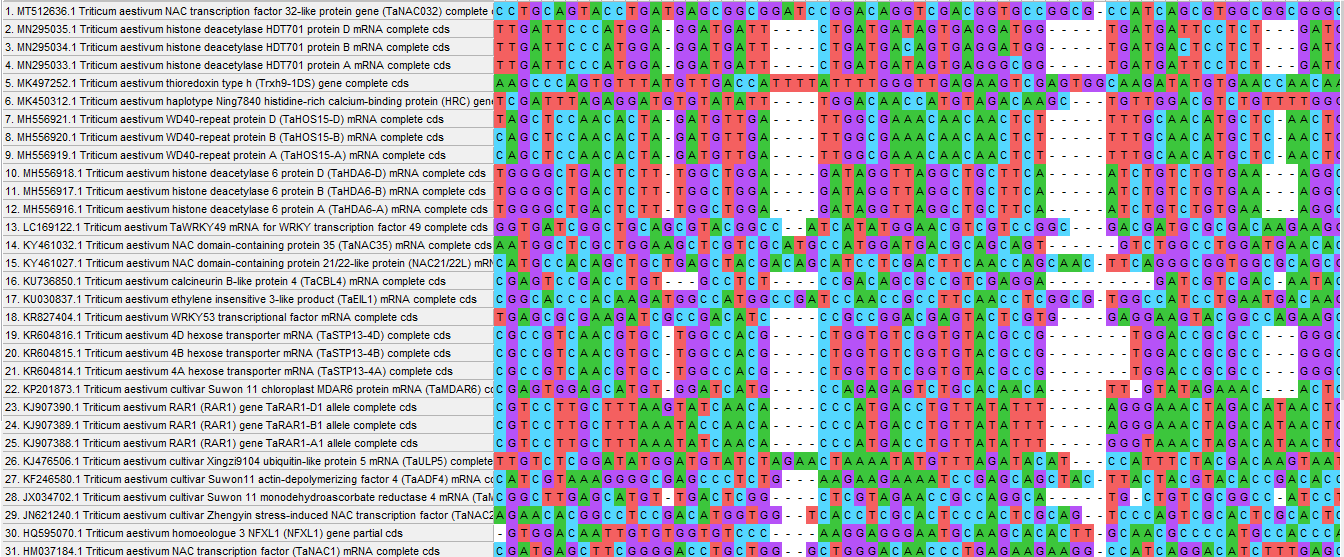


**Figure S2: Multiple Sequence Alignment of the selected S genes using ClustalW**


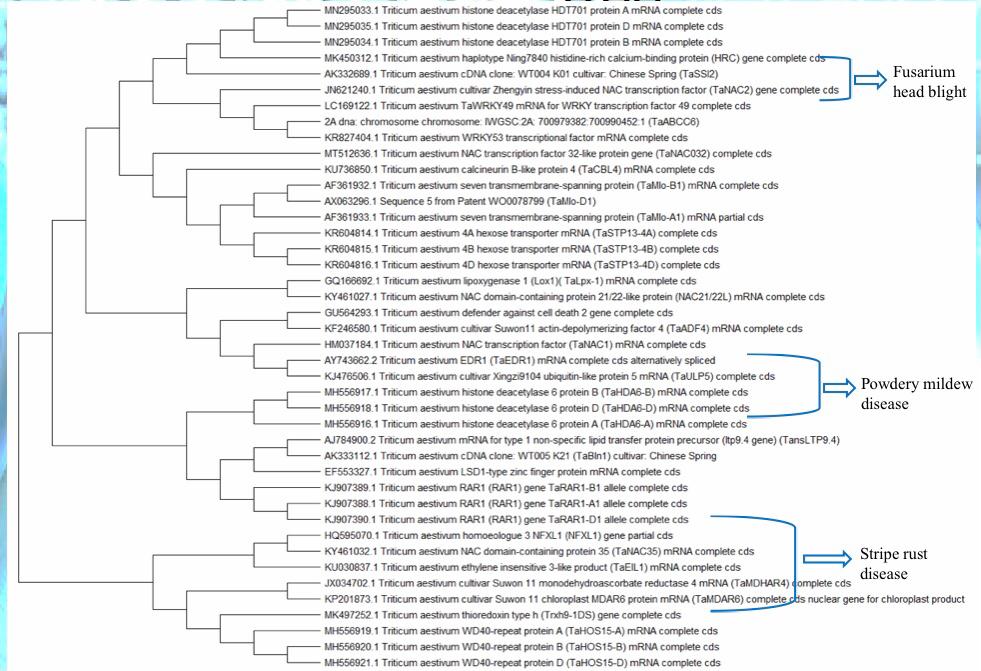


**Figure S3. Construction of phylogenetic tree of identified Negative Regulators Tree using Neighbor Joining Method, MEGA software**
