# Supplementary material for: Potential Targets for CRISPR/Cas Knockdowns to Enhance Genetic Resistance Against Some Diseases in Wheat (Triticum aestivum L.)
Source: Front Genet. 2022 Jun 22;13:926955. doi: 10.3389/fgene.2022.926955 (PMC9245383; doi:10.3389/fgene.2022.926955)
Supplement: Supplementary file 3 [file DataSheet1.DOCX]

***Sequences of Negative Regulators:***

**Table 2: Nucleotide sequences of negative regulators**

| **Nucleotide Sequences of Negative Regulators** | |
| --- | --- |
| TaNAC2 (1222 bp) | >JN621240.1 Triticum aestivum cultivar Zhengyin stress-induced NAC transcription factor (TaNAC2) gene, complete cds  ATGGGGATGCCGGCCGTGAGGAGGAGGGAGAGGGACGCGGAGGCGGAGCTCAACCTGCCCCCCGGCTTCCGCTTCCACCCCACCGACGACGAGCTCGTCGAGCACTACCTCTGCCGCAAGGCGGCGGGGCAGCGCCTCCCGGTCCCCATCATCGCCGAGGTCGACCTCTACCGCTTCGACCCCTGGGCGCTCCCTGACCGCGCCCTCTTCGGCACCCGCGAGTGGTACTTCTTCACCCCCCGCGACCGCAAGTACCCCAACGGCTCCCGACCCAACCGCGCCGCCGGCAACGGCTACTGGAAGGCCACCGGCGCCGACAAGCCCGTCGCGCCCCGCGGCGGGCGGACCATGGGGATCAAGAAGGCCCTCGTGTTTTACGCGGGCAAGGCGCCTAAGGGGGTCAAGACCGACTGGATCATGCATGAGTATCGCCTCGCCGACGCCGGCCGCGCCGCCGCCAGCAAGAAGGGCTCGCTCAGGGTACGTTATTGGTTTGATTATACTTGATGATTTACTGGTTGATTTGGTCGGGATTTCAGATCCCTGCTGCTGCTTCGACTGATTGATTGATTGATTGATTGATTGATTGATTCGGTGCTGATTCCGATCTGAATTTGTTTCTTCAGCTGGACGACTGGGTGCTCTGCCGGCTCTACAACAAGAAGAACGAGTGGGAGAAGATGCAGCTGCAGCAGCAAGGGGAAGAGGAGACGATGATGGAGCCCAAGGCGGAGAACACGGCCTCCGACATGGTGGTCACCTCGCACTCCCACTCGCAGTCCCAGTCGCACTCGCACTCGTGGGGCGAGGCGCGCACGCCGGAGTCGGAGATCGTCGACAACGACCCGTCGCTGTTCCAGCAGGCGACGGCGGCGGCGTTCCAGGCCCAGAGCCCCGCGGCCGCCGCGGCGCACCAGGAGATGATGGCCACGCTGATGGTGCCCAAGAAGGAGGCGGCGGACGAGGCCGGCAGGAACGACCTCTTCGTCGACCTCAGCTACGACGACATCCAGAGCATGTACAACGGCCTCGACATGATGCCGCCCGGGGACGACCTGCTCTACTCCTCCCTCTTCGCCTCCCCCCGTGTCCGCGGGAGCCAGCCCGGCGCCGGCGGCATGCCGGCCCCGTTCTAAGCAGAGCAAAGACAGAGACCGTCAGAGACCCCGAGATCGACAGAAGTGGAATGGACGACTTCTTGGCCGCGAGCGAGCGAGACCGC |
| TaTrxh9 (2307 bp) | >MK497252.1 Triticum aestivum thioredoxin type h (Trxh9-1DS) gene, complete cds  CCAGGCTTATCTTCTCCGTCTCCTCCGACCTCGCCTCGCCCCCTCGCCCCGCGGCTTCTGGGCTCCTTGGCGCCAAAATCCCCGCTTCCGATCTCAGGTAAATCCTCTCGCTGATCTGGTCGTCTTCGATTTGTCCGATGCGGTTCTTGAGCGCGAATTCGCCTGCAAGATTCTGGTTCTTGATCCGCGAGGGAGCGGAGACAACTGGATTTTTGTGTGCGGGTGTGTGTGTGTCGGTGGGTGGGTGCTTTCTTTCCCCCGAGCTCCCTCCTGGGTTATTATGTGTTTAGTTTAAAATCAGGTTCAGGTTCTTGTCCGGTTCTTTTTTAGATAATGGAAGAATTCCGACCTCTGCATCTGGAGATGCGCACACGGCCAGCGTAGCTCATCACAATTGCAAACAGATGCAGCGGTAGTTCCTCGCATCCAGTTGTGATGGGGTAGGGAGAAAAATACTCTCCTCAGTCCATTTGACGACAAGTTATCTGTTATACTGCTATTTCCATCATGGAGTAAATACCTTGCTGAAATTATTGTTCGTGCAACGTCTCCATATTTTTTTAGTGTTACAAACACAGTTTCCTTCTGATAGACCATGACATATTCTACTATTGTTTAGGCCTTCGGGAATCAGGGGGCCTTTCATTGAAGTTCACGATGGGGGGCTGTGTGGGCAAGGTGAGTTCGTGTCTGTTACCGAATCTGTGTTTTGTTCTTGAACTCCCGAGTGATTTTTATGTAATCCTCCTGTCCTCTTTTGGTCAGGGTCGTAGCATCGTGGAAGAAAAGCTTGATTTCAAAGGTGGAAATGTGCATGTCATAACAACCAAAGAGGACTGGGACCAGAAGATTGAAGAAGCAAACAAGGATGGGAAAATTGTAAGCAAACTAGCAATTTGTTACAATGGATTACAGATGTCCTTTGCTTCTTATAGTGTTTGGTATGAACTACTTGATGTTTACGCCTATCAGTTTACACCATATATGAAATGAGAATAGGTCTAAGAAGTCTGTCTACACACACCAATTCCACCATGCCCCTTGTTAGCCTGTAAAATAAGTCCGTTCTTGCAAGAGAACACATTGGCGAGGATGTGGGAATCAAGCACAAATTTCTTTGAGAGACCTAAGCAGATAGTATAACCACCTGCTCAAGCAGAAAGTGTGCACATGAAGGTCCTTAGCTGTTGGGGCATAGACTGTAGAAAGCCTGTAGTGATTAGGGTGTTTATTTTCTTAGTTGTGCACTTGAAATCATGGACAGGTTAACTAAACTATTTTCCTGCAAAAGCAGTTTTGTTAAGGGGATGTTTTCCTTAGCAGAGGGAGGTTCCCTGTAACTGTTCTCCCGCTGAAATCCCGGAATATACACGGGTAGTTGTAGTTATTATGATAGAACATGAAAACCATGCGCTGCAAGCCCAGTGTTTATGTTGACCATTTTATTTTGGGTTGAGAAGTCGAGTGGCAAGATATGTGAACCAACAACTAACAATTCTTGATGCAGTTCCTAGAGTTGCATCTGATGTACTCATTGATTTCTTTTGTAGGTTGTGGCAAACTTCAGCGCTTCGTGGTGTGGGCCATGCCGTGTCATTGCACCTGTTTATGCTGAGATGTCCAAGACTTACCCTCAACTCATGTTCTTGACAATTGATGTTGATGACCTAATGGTAATTCCATCTTCCATTTGTTTGAATTATTTATTCACTACAGGTTAACATGACCCTAAAAAGTCCAGTTGGACAGTTTCTATAACCATTACCATTAAAACCCTGAATAGTTACCGCCGCTTTGTTAAGTCACAAACCTTTCAGCCAAACTTCAAAGTATGCAGTACCGTAGCTTCTCATCTTGCCATTTCCTGTTTCGAAGAAGTATGCAAACAGATAAATACTCCATCTGTACCTTATAAGACGTTTTTAGAGTCCATGACAGTGTCAAAAAACGTCTTATATTAAGTTACAGAGGGAGTATTTGTTACTGCCAATTGTATGGAGTGTGGTGGAGTGTTTTTTTCATCCTGATAGCTCCATGAATAACATGTTTGGTTGATAACACTGTGATTAACAACAGGTTGTTCTGATGCTTCCAGTGTTGACCCACCTTAGTAATTCTCCTTGTGATGATCTGCAGGATTTCAGCTCAACATGGGACATCCGTGCAACCCCGACGTTCTTCTTCCTCAAAAACGGCCAGCAGATCGACAAGCTCGTCGGCGCCAACAAACCTGAGCTCGAGAAGAAAGTACAAGCTCTTGGTGATGGCAGTTGAACCATGCTACAGAATTAGAACAGAGCCAGCTTG |
| TaNAC30 | >SETl_taNAC30 TCCCCAGGGCGAGCCGAAGTCCACCACCAACCCATCCCCCCACCCCCCTCGACCGCCTCCGCGCCGGCCGCCGAGATGGAGACCCCGCCGCCGCCGCCGCGCTGGCCGCCGGGCTTCCGCTTCAGCCCCACCGACGAGGAGCTCGTCCTCTTCTTCCTCAAGCGCCGGGTCGCCGCCGGCCGCCCCTCCCCCTACATCGCCGACGTCGACGTCTACAAGTCCCACCCCTCCCACCTCCCCGAGAGGTCGGCGCTGCGGACGGGGGACAAGCAGTGGTTCTTCTGCAGCCGGCTGGACCGCAAGTACCCCAACGGCTCGCGCGCCAGCCGCACCACCGCCGACGGCTACTGGAAGGCCACGGGCAAGGACCGCTCCATCTGCAACGCCGGCCGCGCCGTGGGGAACAAGAAGACGCTCGTCTACCACCACGGCCGCGCGCCGCGCGGGGAGCGCACCGACTGGGTCATGCACGAGTACACCATCCTCGCCGACGCGCTCCCGCCGCCCGCACGGTGCCGCGAGTCCTACGCCCTCTACAAGCTCTTCGAGAAGAGCGGGGTCGGCCCCAAGAACGGCGAGCAGTACGGCGCGCCCTTCCGGGAGGAGGACTGGCTGGACGACGACGACGACTGCGAGCTGCCCTCTGATCCCATCCCTATCGCCGTCAGTCTACCTAGAGCCGTGACAGTGGACGAGCAGATTGGGGATGTTTTGACAGTTGTCGAGCAGATTGGGGGTGTGACAGTGGACAAGCAGATTGGGGTTATGACAGTGGACGACCAGCTTGGGGATCTTGAGGTGTTCCTGTTGCAAAATGGAGATGATCTGGGAAACAGTGAACCACAGTCGGATTTCTCGACACCGGTTTCTTCACAGGCTCCGCTTCAGCATGGCCATCCTCAAGGTCGGCCCAGCGATGATGGTAACATATCTGAGGTCGCAGATGCTACAACCAGCAGCCGTGGTATGGTAATGGCGGTGAATACGTGCACTGAACTCCCTTTTGGGGATCTTGAGGGGCTACTGATGGAAATATCGGATGATCAACGGGCCACTGAATCGTTCGAAGAATTCTCAGAATTCATTCCCCAACTGCAGCTTCAGCATGATGATCATGAGCATGAGGCTTGGCCCAATGCCAACATGGAGGAGATTAGTGTTGCGGATTATGCCACTAGTAATGGCGTTGTGGACGCTTCAGGATGTACTGTCACCGAGCTTCCCTATGAGGATATTGAAGGGCTTCTGTTGCAATTAGAGAATGACCAGGGAAATGTCCAACCACTGGCAGATTTCTCCACACCAGTTCCTCGTCATGAGTTCCATCAGGTTGGCTCTGGAGATTTCCATGGATGTCACGGTGCTACATTCAACTCTGTAGATCCTTCTTCTGCAGTGCAAGAAAACAGAGATCTTGATCCACGATCGGAGCCCAGTAATCAAATTACACAGTCTGCCCTCACCAACATGCCATTGAATTGGGAAACAGATTGCACTGAAGAAACAAGTGCGCTGCGATCTGTGTCAGGGTTGGCCAGTTATGACGGTCAGGATGCTGAGGAGGAGTTCCTTGAAATCAACGATTTCTTGGATCCAGAAGATGTAGGACAGAGTATGAATTGTACTGCAACTGAGCACTTAATTTCTGCATCCAATGGTATGTTTGACAGTTTGGAGTTTGCCGACGCTTCCATGTTTCTACCTGGCTCATTTGACACAGCTGGAGTGGCGACTGAAAACCAGTTTGGTTATCTTGGTGATAGTGGATCCCAGAACCAGGGATTCCAGTATACATCTGAATCGTGGACGCACAACCAGGTCGCTCTGAATGTGCGGAACCACATGAATCATAACCATGTTGTCTTCTCACATGCATCAGGCACTGCGAATTTCCACACGGTGAATGAGCAACCACATAACCTGAGTCCAGCGGATTCGCAGTCATGGTTCAATTCAGCTGTGTCAGCCCTGCTGGATGCGGTACCTGCCAATCCAGTTTTGGCGGCTGAAAACAATGTTTTCAACAGAACACTCCAACGCATTTTTAGCTTTAGGTCAGACCAAGCTCCGAAGGAAGAAGCAAGCGCCCCAGTTATCCAGGTTAGAAGAAGGGGCGCAGGGCTGATTTCGGTCTCGCTACTGGTTTTGCTTGCTGCCATCATGTGGCCCTTTGCTGCTGGCCCCGGGTACGCAATCAAGTTTTGCAAGGGGTTATGGAAGTCCTCCTTTCCATGCCAAGATGATGCCCAAAGTTTGCTCGACTTACCCTAGCCGTTGATCCTCATCATTACCCCTCAGTTGCTAGTTATTGTTTTGCTATCATTCCCATTGGAAAAAGGGGAAGAGGTAGAAGCATTCCCATTGGAAAAAGGCGAAGGGGTAGAAGCATTTCCAAAGTGTGATTTGAGGTGTTAGTTTTAAGTTCATGCAGGGGGCTTGGAAAACCCAAAAAAGAAAGAAAAAAAATAACTAATGGTAATATATATATATAACTACTTCTCCTCCTTAAAAAAAAAA |
| *TaNAC1*  (1227 bp) | >HM037184.1 Triticum aestivum NAC transcription factor (TNAC1) mRNA, complete cds  TAAGGTAGCAGTGTAGCGATCCGACCGAGAAGATGAGCTCTATTGGCATGATGGAGGCGAGGATGCCGCCGGGGTTCCGGTTCCACCCACGGGACGAGGAGCTGGTCCTCGACTACCTCCTCCACAAGCTCACCGGCCGGCGCGCATACGGTGGCGTCGACATCGTGGACGTCGACCTCAACAAGTGCGAGCCATGGGACCTTCCAGAGGCGGCGTGCGTGGGCGGCAGGGAGTGGTACTTCTTCAGCCTGCGCGACCGCAAGTACGCCACCGGCCAGCGCACCAACCGCGCCACGCGCTCCGGCTACTGGAAGGCCACCGGCAAGGACCGCGCCATCCTCGCGCACGGCGAGGCGTTGGTGGGGATGCGCAAGACGCTCGTCTTCTACCAGGGGAGGGCCCCCAAGGGGACGAGAACGGAGTGGGTCATGCACGAGTTCCGCCTCGAGGAGGAGCGGCACCGCCACCACCACCAGCAGAAGGGCGGCGCCGCCGCCGCCGAGGCGAGGTGCCAGCTCAAGGAAGACTGGGTGCTATGCAGGGTGTTCTACAAGAGCAGAACAAGCAGCCCAAGGCCACCATCTGAAGAAGTCTACACATTTTTTAGCGAGCTGGACCTTCCGACTATGCCACCGCTCGCGCCCCTCATCGACGCGTGCATCGCCTTCGACAGCGGCACCGCGATGAACACCATCGAGCAAGCGTCCTGCTTCTCCGGCCTGCCAGCACTACCCCTCAGGGGATCGATGAGCTTCGGGGACCTGCTGGGCTGGGACAACCCTGAGAAGAAGGCCATCAGGACATCTTTGAGCAACATGTCAAGTAACAGCAATTCCAAGTTGGAGTTGACCCCAAACTGGAGCCAGGAGAACGGCTTGTCACAGATGTGGACACCCCTTTGAATCTTGATATGAGACTATTGTGCTGCTAGTTGAAGTGTGGTGGTGGCTAGTAAGTAATTAGATGACATATTCTTTCTAATGATATTGGAGTAGCATTGGACTATTGGTTGATGCAGTGTCTGTTCCTAGGGATTTGATGGAAGTGTGTGTTGATTTACAAACCAGTAGATTAGTGGAATGGCTTGATATAGCATCTGTACATAGGAATGCTAAGAAATGTCCTATTTTAATTTTACTATTTCTTTTTACTCTCCAAATGTAATGTAACCTCCAGCTGAAAGTAAATACTATGTGGATAAAAAAAAAAAAAAAAAAAAAAAAAAAA |
| TaNAC35 (1401 bp) | >KY461032.1 Triticum aestivum NAC domain-containing protein 35 (TaNAC35) mRNA, complete cds  CCACACTCCGCTCTCGGTCCTTCCCGTCGCAGCGCTGCCGCGCCGGGCGGCCATGGCAGAGTTGAGGAATTGCTTGCATGGATGGAGTGCCCAAATTGCAAATACCGCATTGATAATACTGATGTTTTATCACAGTGGCCAGGACTCCCTGCTGGTGTCAAATTTGATCCAACTGATCTCGAACTGCTTGAACATTTAGAAGGAAAGGTTGGCAGGGTAGCGTCCCATGTACTAATAGATGATTTTATTCCAACCATAAAGGAGGCCCAAGGAATCTGCTATACACATCCGGAAAATCTCCCTGGTATCAAGATGGACGGGAGCACCGGTCACTTCTTCCACAAAGTATCCAATGCATACGTTGTTGGCAAGCGTAAGCGTCGCAAGATTAGCAACAGTGACCACACTGTTTGTGATGAGAATCTCAGATGGCACAAGACTGGAAAATCCAGATCTATCTTAGATAATAACGGTGTCATAAAAGGGTGGAAGAAAATATTGGTTCTTTACATAGGTTATCGGAAAGGAGGTGGCAAGACAGAGAAAACTAATTGGAGAATGCATCAGTACCACCTTGGGGTAGATCAAGATGAAAAGCAGGAGGAGCTTGTTGTTTCCAAAGTCTTTTATCAGGTGCAGTCAATGAATGCTGGGCAGTCTCTAGTGTGCGGTGTTAATGAAGAATTCGATTCATTTGCTGGGGAAGACGATCCTACAACCCCAATGACATACCCTCTGCAGACTCGTTGCCCAAACGGCAGCCCATCCGGAACCGAGCAGAATCAGGAGGAGGGAGAGTCCCGCGTGTCTACAGTTCGGGAAGCGGGGGAATGGCTCGCTGGAAGCTCGTCGCATGCCATGGATGACGCAGCAGTGTCTGGCCTGGATGAACACCTGTCACACGGCAGAATCCCGGAGGAGGCGCTCCTGGGGTTCCCCGACCTCGGAATGCCTCCGGATGTCTCCCTTCTCACCGACATGCAGTTGGGGTCGCAGGATAGCATGGAGATGTGGCTGGTTAGCGTGTTAACAGAGGATGAGGGGGTATTTGAAGCAGCGGAGCAACGAGAAGAGGCTGGTTTTTCTCCGTGATGTAGTAGTACTCCCTCCGTCCGGAAATACTTGTCATCAAAATAAATAAAAGGGGATGTATCTAGATACATCCCTTTTTGTCCGTTTTGATGACAAGTATTTTCGGACGGAGGAAGTATGTTGTATCTCTGTGCATGAGTGTTGGATCTCTGAGAAAATTTTGTATATGATCCTGGAGCTTGCCAACTGAACGTAGCTCGGCTATGTTTAATGTTTGTAGTAATAGCAGTCATTTATTTTCAAATGTAAATGTGGGAGGGCCGCTCCCAATTTTATAGCCATGAAAGTCTGAATGATATAAAAAAAAAA |
| TaLpx-1 (2586 bp) | >GQ166692.1 Triticum aestivum lipoxygenase 1 (Lox1)( TaLpx-1) mRNA, complete cds  ATGATACTGGGCGGGCTCATCGACGGCCTGACGGGGGCGAACAAGAGCGCGCGGCTCAAGGGCACGGTGGTGCTCATGAGGAAGAACGTGCTGGACCTCACCGGCTTCGGCGCCACCATCATGGACGGCATCGGCGACTTCTTCGGCAAGGGCGTCACCTGCCAGCTTATCAGCTCCACCCTCATCGACCGCGACAACGGCGGGCGTGGGAAGGTGGGCGCGGAGGCGGAGCTGGAGCAGTGGGTGACGAGCCTGCCGTCGCTGACCACGGGGGAGTCCAAGTTCGGCCTCACCTTCGACTGGGAGGTGGAGAAGCTGGGCGTGCCGGGCGCCATCATCGTCAACAACCACCACAGCTCCGAGTTCCTGCTCAAGACCGTCACCCTCCACGACGTCCCCGGCCGCGGCAACCTCTCCTTCGTCGCCAACTCATGGATCTACCCCGCCTCCAGCTACACCTACAGCCGTGTCTTCTTCGCCAACGATACGTACCTGCCGAGCCAGATGCCGGCGGCGCTGAAGCCGTACCGCGACGACGAGCTCCGTAACCTGCGGGGCGACGACCAGCAGGGCCCCTACCAGGAGCACGACCGCGTCTACCGCTACGACGTCTACAACGACCTCGGCGAGGGCCGCCCCGTCCTCGGCGGCAGCGCCGACCACCCCTACCCGCGCCGCGGACGCACCGGGCGCAAGCCCAACGCCAACGATCCCAGCCTGGAGAGCCGGCTGTCGCTGCTAGAGCAGATCTACGTGCCGCGGGACGAGAAGTTCGGCCACCTCAAGACCTCCGACTTCCTGGGCTACTCCATCAAGGCCATCACGCAGGGCATCCTGCCGGCGGTGCGCACCTACGTCGACACCACCCCCGGCGAGTTCGACTCCTTCCAGGACATCATCAACCTCTACGAGGGCGGCATCAAGCTGCCCAACGTCCCCGCCCTCGAGGAGCTGCGCAAGCAGTTCCCGCTCCAGCTCATCAAGGACCTCCTCCCCGTGGGCGGCGACTCGCTGCTCAAGCTCCCCGTCCCCCACATCATCCAGGCGGACAAGCAGGCGTGGAGGACCGACGAGGAGTTCGCCCGGGAGGTGCTCGCCGGCGTCAACCCGGTCATGATCACGCGTCTCACGGAGTTCCCGCCAAAAAGTAGTCTGGACCCTAGCAAGTTTGGTGACCACACCAGCACCATCACGGCGGCACACATCGAGAAGAACCTCGAGGGCCTCACCGTGCAGCAGGCGCTGGAAAGCAACAGGCTGTACATCCTTGAGCACCACGACCGGTTCATGCCGTTCCTCATCGACGTCAACAACCTGCCCGGCAACTTCATCTACGCCACCAGGACCCTCTTCTTCCTGCGCGGCGACGGCAGGCTCACGCCGCTCGCCATCGAGCTCAGCGAGCCTGTCATCCAGGGCGGCCTCACCACCGCCAAGAGCAAGGTGTACACGCCGGTGCCGAGCGGCTCCGTCGAAGGCTGGGTGTGGGAGTTCGCCAAGGCCTACGTCGCCGTCAACGACTCCGGGTGGCACCAGCTCGTCAGCCACTGGCTGAACACCCACGCGGTGATGGAGCCGTTCGTGATCTCGACGAACCGGCAGCTCAGCGTGACGCACCCGGTGTACAAGCTGCTGAGCCCGCACTACCGCGACACGATGACCATCAACGCGCTAGCGCGGCAGACGCTCATCAACGCCGGCGGCATCTTCGAGATGACGGTGTTCCCGGGCAAGTTCGCGTTGGGGATGTCGTCAGTGGTGTACAAGGACTGGAAGTTCACGGAGCAGGGCCTGCCCGACGATCTCATCAAGAGGGGCATGGCGGTGGAGGACCCGTCGAGCCCGTACAAGGTGAGGCTGCTGGTGTCTGACTACCCGTACGCGGCGGACGGGCTGGCGATCTGGCACGCCATCGAGCAGTACGTGAGCGAGTACCTGGCCATTTACTACCCGAACGATGGCGTGGTGCAGGGCGACGTGGAGCTGCAGGCGTGGTGGAAGGAGGTGCGCGAGGTGGGGCACGGCGACCTCAAGGTCGCGCCATGGTGGCCGAGGATGCAAGCCGTGGGCGAGCTGGCCAAGGCGTGCACCACCATCATCTGGATCGGGTCGGCGCTGCATGCGGCGGTCAACTTCGGGCAGTACCCCTACGCGGGGTTCCTCCCGAACCGGCCGACGGTGAGCCGGCGCCGCATGCCGGAGCCGGGGACCGAGCAGTACGCGGAGCTGGAGCGCGACCCGGAGCGGGCCTTCATCCACACCATCACTAGCCAGATCCAGACCATCATCGGCATCTCGCTGCTGGAGGTGCTGTCGAAGCACTCCTCCGACGAGCTCTACCTCGGGCAGCGTGACACGCCGGAGTGGACCTCGGACCCCAAGGCCCTGGAGGTGTTCAAGCGGTTCAGCGAGCGGCTAGTGGAGATCGAGAGCAAGGTGGTGGGCATGAACCACGACCCGCAGCTGTTGAACCGCAACGGTCCGGCCAAGTTCCCCTACATGTTGCTCTACCCCAACACCTCCGATCACAAGGGCGCCGCCGCCGGGCTCACCGCTAAGGGCATCCCAAACAGCATTTCCATCTAA |
| TaMLO (1399 bp, 1730 bp, 1693 bp) | >AF361933.1 Triticum aestivum seven transmembrane-spanning protein (TaMlo-A1) mRNA, partial cds  GTGGAACCCGGTTCCGTCAAGAGCAAGTACAAGGACTACTACTGCGCCAAAGAGGGCAAGGTGGCGCTCATGTCCACGGGCAGCCTGCACCAGCTCCACATATTCATCTTCGTGCTAGCCGTCTTCCATGTCACCTACAGCGTCATCATCATGGCTCTAAGCCGTCTCAAGATGAGAACATGGAAGAAATGGGAGACAGAGACCGCCTCCTTGGAATACCAGTTCGCAAATGATCCTGCGCGGCTCCGCTTCACGCACCAGACGTCGTTCGTGAAGCGGCACCTGGGCCTGTCCAGCACCCCCGGCGTCAGATGGGTGGTGGCCTTCTTCAGGCAGTTCTTCAGGTCGGTCACCAAGGTGGACTACCTCACCTTGAGGGCAGGCTTCATCAACGCGCACTTGTCGCAGAACAGCAAGTTCGACTTCCACAAGTACATCAAGAGGTCCATGGAGGACGACTTCAAAGTCGTCGTTGGCATCAGCCTCCCGCTGTGGGCTGTGGCGATCCTCACCCTCTTCCTTGATATCGACGGGATCGGCACACTCACCTGGGTTTCTTTCATCCCTCTCATCATCCTCTTGTGTGTTGGAACCAAGCTAGAGATGATCATCATGGAGATGGCCCTGGAGATCCAGGACCGGTCGAGCGTCATCAAGGGGGCACCCGTGGTCGAGCCCAGCAACAAGTTCTTCTGGTTCCACCGCCCCGACTGGGTCCTCTTCTTCATACACCTGACGCTGTTCCAGAACGCGTTTCAGATGGCACATTTCGTGTGGACAGTGGCCACGCCCGGCTTGAAGGACTGCTTCCATATGAACATCGGGCTGAGCATCATGAAGGTCGTGCTGGGGCTGGCTCTCCAGTTCCTGTGCAGCTACATCACCTTCCCCCTCTACGCGCTAGTCACACAGATGGGATCAAACATGAAGAGGTCCATCTTCGACGAGCAGACAGCCAAGGCGCTGACCAACTGGCGGAACACGGCCAAGGAGAAGAAGAAGGTCCGAGACACGGACATGCTGATGGCGCAGATGATCGGCGACGCAACACCCAGCCGAGGCACGTCCCCGATGCCTAGCCGGGGCTCATCGCCGGTGCACCTGCTTCAGAAGGGCATGGGACGGTCTGACGATCCCCAGAGCGCACCGACCTCGCCAAGGACCATGGAGGAGGCTAGGGACATGTACCCGGTTGTGGTGGCGCATCCTGTACACAGACTAAATCCTGCTGACAGGCGGAGGTCGGTCTCTTCATCAGCCCTCGATGCCGACATCCCCAGCGCAGATTTTTCCTTCAGCCAGGGATGAGACAAGTTTCTGTATTGATGTTAGTCCAATGTATAGCCAACATAGGATGTGATGATTCGTACAATAAGAAATACAATTTTTTAAAAAAAAA  >AF361932.1 Triticum aestivum seven transmembrane-spanning protein (TaMlo-B1) mRNA, complete cds  AAAGAGGTTGCGCTCGGGGACCGATGGCGGACGACGACGAGTACCCCCCAGCGAGGACGCTGCCGGAGACGCCGTCCTGGGCGGTGGCCCTCGTCTTCGCCGTCATGATCATCGTGTCCGTCCTCCTGGAGCACGCGCTCCATAAGCTCGGCCATTGGTTCCACAAGCGGCACAAGAACGCGCTGGCGGAGGCGCTGGAGAAGATCAAGGCGGAGCTCATGCTGGTGGGCTTCATCTCGCTGCTGCTCGCCGTGACGCAGGACCCCATCTCCGGGATATGCATCTCCGAGAAGGCCGCCAGCATCATGCGGCCCTGCAAGCTGCCCCCTGGCTCCGTCAAGAGCAAGTACAAAGACTACTACTGCGCCAAACAGGGCAAGGTGTCGCTCATGTCCACGGGCAGCTTGCACCAGCTGCACATATTCATCTTCGTGCTCGCCGTCTTCCATGTCACCTACAGCGTCATCATCATGGCTCTAAGCCGTCTCAAAATGAGAACCTGGAAGAAATGGGAGACAGAGACCGCCTCCCTGGAATACCAGTTCGCAAATGATCCTGCGCGGTTCCGCTTCACGCACCAGACGTCGTTCGTGAAGCGGCACCTGGGCCTCTCCAGCACCCCCGGCGTCAGATGGGTGGTGGCCTTCTTCAGGCAGTTCTTCAGGTCGGTCACCAAGGTGGACTACTTCACCTTGAGGGCAGGCTTCATCAACGCGCATTTGTCGCATAACAGCAAGTTCGACTTCCACAAGTACATCAAGAGGTCCATGGAGGACGACTTCAAAGTCGTCGTTGGCATCAGCCTCCCGCTGTGGTGTGTGGCGATCCTCACCCTCTTCCTTGACATTGACGGGATCGGCACGCTCACCTGGATTTCTTTCATCCCTCTCGTCATCCTCTTGTGTGTTGGAACCAAGCTGGAGATGATCATCATGGAGATGGCCCTGGAGATCCAGGACCGGGCGAGCGTCATCAAGGGGGCGCCCGTGGTTGAGCCCAGCAACAAGTTCTTCTGGTTCCACCGCCCCGACTGGGTCCTCTTCTTCATACACCTGACGCTATTCCAGAACGCGTTTCAGATGGCACATTTCGTGTGGACAGTGGCCACGCCCGGCTTGAAGAAATGCTTCCATATGCACATCGGGCTGAGCATCATGAAGGTCGTGCTGGGGCTGGCTCTTCAGTTCCTCTGCAGCTATATCACCTTCCCGCTCTACGCGCTCGTCACACAGATGGGATCAAACATGAAGAGGTCCATCTTCGACGAGCAGACGGCCAAGGCGCTAACAAACTGGCGGAACACGGCCAAGGAGAAGAAGAAGGTCCGAGACACGGACATGCTGATGGCGCAGATGATCGGCGACGCGACGCCCAGCCGAGGGGCGTCGCCCATGCCTAGCCGGGGCTCGTCGCCAGTGCACCTGCTTCACAAGGGCATGGGACGGTCCGACGATCCCCAGAGCACGCCAACCTCGCCAAGGGCCATGGAGGAGGCTAGGGACATGTACCCGGTTGTGGTGGCGCATCCAGTGCACAGACTAAATCCTGCTGACAGGAGAAGGTCGGTCTCGTCGTCGGCACTCGATGTCGACATTCCCAGCGCAGATTTTTCCTTCAGCCAGGGATGAGACAAGTTTCTGTATTGATGTTAGTCCAATGTATAGCCAACATAGGATGTCATGATTCGTACAATAAGAAATACAAATTTTTACTGAGTCAAAAAAAAAAAA  >AX063296.1 Sequence 5 from Patent WO0078799 (TaMlo-D1)  ATGGCGGAGGACTACGAGTACCCCCCGGCGCGGACGCTGCCGGAGACGCCGTCCTGGGCGGTGGCGCTCGTCTTCGCCGTCATGATCATCGTGTCCGTCCTCCTGGAGCACGCGCTCCACAAGCTCGGCCATTGGTTCCACAAGCGGCACAAGAACGCGCTGGCGGAGGCGCTGGAGAAGATCAAAGCGGAGCTGATGCTGGTGGGGTTCATCTCGCTGCTGCTCGCCGTGACGCAGGACCCAATCTCCGGGATATGCATCTCCGAGAAGGCCGCCAGCATCATGCGGCCCTGCAGCCTGCCCCCTGGTTCCGTCAAGAGCAAGTACAAAGACTACTACTGCGCCAAAAAGGGCAAGGTGTCGCTAATGTCCACGGGCAGCTTGCACCAGCTCCACATGTTCATCTTCGTGCTCGCCGTCTTCCATGTCACCTACAGCGTCATCATCATGGCTCTAAGCCGTCTCAAAATGAGGACATGGAAGAAATGGGAGACAGAGACCGYCTCCTTGGAATACCAGTTCGCAAATGATCCTGCGCGGTTCCGCTTCACGCACCAGACGTCGTTCGTGAAGCGTCACCTGGGCCTCTCCAGCACCCCCGGCATCAGATGGGTGGTGGCCTTCTTCAGGCAGTTCTTCAGGTCGGTCACCAAGGTGGACTACCTCACCCTGAGGGCAGGCTTCATCAACGCGCATTTGTCGCATAACAGCAAGTTCGACTTCCACAAGTACATCAAGAGGTCCATGGAGGACGACTTCAAAGTCGTCGTTGGCATCAGCCTCCCGCTGTGGTGTGTGGCGATCCTCACCCTCTTCCTTGATATTGACGGGATCGGCACGCTCACCTGGATTTCTTTCATCCCTCTCGTCATCCTCTTGTGTGTTGGAACCAAGCTGGAGATGATCATCATGGAGATGGCCCTGGAGATCCAGGACCGGGCGAGCGTCATCAAGGGGGCGCCCGTGGTTGAGCCCAGCAACAAGTTCTTCTGGTTCCACCGCCCCGACTGGGTCCTCTTCTTCATACACCTGACGCTGTTCCAGAATGCGTTTCAGATGGCACATTTCGTCTGGACAGTGGCCACGCCCGGCTTGAAGAAATGCTTCCATATGCACATCGGTCTGAGCATCATGAAGGTCGTGCTGGGGCTGGCTCTTCAGTTCCTCTGCAGCTATATCACCTTCCCCCTCTACGCGCTCGTCACACAGATGGGATCGAACATGAAGAGGTCCATCTTCGACGAGCAGACGGCCAAGGCGCTGACCAACTGGCGGAACACGGCCAAGGAGAAGAAGAAGGTCCGAGACACGGACATGCTGATGGCGCAGATGATCGGCGACGCGACGCCCAGCCGAGGCACGTCGCCGATGCCTAGCCGGGCTTCGTCACCGGTGCACCTGCTTCACAAGGGCATGGGACGGTCCGACGATCCCCAGAGCGCGCCGACCTCGCCAAGGACCATGGAGGAGGCTAGGGACATGTACCCGGTTGTGGTGGCGCATCCCGTGCACAGACTAAATCCTGCTGACAGGCGGAGGTCGGTCTCTTCGTCGGCACTCGATGCCGACATCCCCAGCGCAGATTTTTCCTTCAGCCAGGGATGAGACAAGTTTCTGTATTGATGTTAGTCCAATGTATAGCCAACATAGGATGTCATGATTCGTACAATAAGAAATACAAATTTTTACTGAG |
| TaEDR1 (3050 bp) | >AY743662.2 Triticum aestivum EDR1 (TaEDR1) mRNA, complete cds, alternatively spliced  CGCCAAACTGAAATAACCAAGAGAGGAAATATTTTCGAAACCCAAACCCTAGGTGCTCGTGGGGAGCGCCATGAAGATCCCGTTTGTGACCAAGTGGTCGCACCGATCCAGCGAGCCCGCGGGGCCGTCGAATTCGGCTGCAGCGCAGCAGCAGCAGCAGCAGCAGGCGCCGTCTCCTCCTCCTCCCGTGGCGTCGACAGAGGCGGCAGGGGATGAGTTCATTCTGCAGGAGGAAGAGTACCAGATGCAACTGGCGTTGGCGCTATCAGCGTCGGCGTCGGGCGCCGAGGGCGCTGGGGATCCCGACGGGGAGCAGATCAGAAAGGCGAAGCTGATGAGCCTCGGGAAGGGCCACCCGGTCACCAACAGCGATCGTGGCGGGGGAGACACCCCGGAGTCGCTCTCCCGCCGTTACAGGGACTATAACTTTCTTGATTACAATGAGAAAGTAATTGATGGATTCTACGACGTATTTGGCCTCTCTGCGGGATCATCTGGGCAGGGCAAAATACCTTCACTGGCAGAGCTTCAGATGAGCATTGGGGATCTTGGATATGAAGTAATTGTGGTTGACTATAAATTTGATAATGCTCTGCAGGAGATGAAGGAAGTAGCAGAATGCTGCCTGTTGGGCTGTCCTGACATTACAGTATTGGTGCGACGAATAGCTGAAGTTGTTGCAGATCACATGGGTGGTCCAGTGATCGATGCAAATGAAATGATCACTAGGTGGTTGAGCAAAAGCATTGAGCAGAGGACATCACACCAGACAAGCCTTCTGCATATTGGCAGTATAGAGATAGGCTTGTCTCGCCATCGTGCCTTACTTTTCAAGATTCTTGCTGATATTGTTGGTATCCCTTGCAAGCTGGTTAAAGGGAGTCATTACACTGGTGTTGAAGATGACGCTATTAACATAATAAAGATGGATGACAAAAGGGAGTTTTTGGTGGATGTTATGGCTGCTCCAGGGACTCTCATTCCAGCAGATGTCTTTAATTCAAAGGGTACTCCATTCAACTTCAGTCAAACATTGGGTCAGAATCAGGTGGTGGAGTCAGCAAGTAACATCGAAGATGACCCAGTTGCATTACAGTCAGAGCATAAACGTAACCAAGGGCATATGTTTGCCAATAATAATCGGATCTCAGTCAATCTATCAAGCTATGAGAATACAATGACCGCTGGAAGTAGTGCTAGTGAACCTGGGACATTGGACCCTAGGATGCAATTAGGTAAAACATCAACTTTGCCTAGTGCTCCTTCCAAGCAGAAGAAGAATCTGCAATTGATTACAGACTCTCATGAAACTGAAGAGTCCCGAAAACTATTTGTGGAGTTAGATCCTTTCAATGCTATTGAATCTGGGAAAAGCTCATTGGCATTCAAGGGATTAAATAATAGAAACAATGAATTCCAAAGGCGTAGAGAGAATGTAGTCCCACCATCTGTAAGATCTCAACAGCCATTGGTGATGAAAAACTGGTCTGCTTGCAATGACATTTCCAACAACAAGCAATACAATGTTGCTGATGGGTCAGTTCCTCGGAGAAATGCCACTGACAATGCATCGTCATCTCAGTTGGCGTTGTCAACTGCAAAGCATTACAATTCCAATGTTAGAGAGCTAAACGATAGAGTGTATGCAGCACCTGCTCGTAATTATGACAACAAGATAGTTGGTACCTCGGCTATGGCCAAAGCATTGACTGGAGAGTGCCCTGACAGATCACAGGTGCCACCTGGTCTTTATTATGACAAGATGCTTGGTACCTCTTCTATGAATGCAGCTTCTACATCCGGAATCGGGAAAGTTGCAGAAAAGGACCCTCATAATGATCCGGGAAAAGGTCCCATCTATTCTAGATTTGATGGTGAACTTTCTAAAAATGCTCAAGGATTTACTCCCGAAAGGGATGAGCACAAGGAAAATTGTGGCAGTCATGACCACAAAATGTTATATCCTGATCCAAGAAAGTCCCCTCTTGACAGATTCATGGACAGGCCAAGGCAGAGCATAGAATGTGTTTTTCCATCCCAAGTTGGATCAAATAAGGCTGACATGGTGTTGGATGAAGTGTCTGAATGTGAAATCCTTTGGGAAGATCTTGTAATCGATGAAAGAATTGGCATAGGTTCATATGGAGAAGTCTACCATGCTGATTGGAATGGAACTGAAGTAGCTGTAAAGAAGTTCTTGGATCAAGAGTTCTATGGTGATGCTTTAGAGGAATTTCGTTGTGAAGTGAGGATTATGCGTCGGCTCCGTCATCCAAATATTGTTCTCTTTATGGGTGCAGTAACACGGCCTCCACACTTATCTATTGTATCAGAATATCTTCCAAGGGGAAGCTTATATAAGATCATTCATCGCCCTAATTGCCAAATCGACGAGAAGCGTAGGATTAAAATGGCCCTTGATGTGGCCAGAGGCATGAATTGTCTTCATACCAGTGTACCAACAATTGTTCACCGGGATCTAAAATCACCAAACTTGCTGGTTGACGATAATTGGACTGTGAAGGTCTGTGATTTCGGACTTTCACGTCTGAAGCACAGTACATTTTTGTCATCAAAATCCACTGCCGGGACTCCTGAGTGGATGGCACCAGAGGTTTTGCGGAATGAGCAATCCAATGAGAAGTGTGATATTTACAGCTTTGGTGTTATCCTGTGGGAGCTAGCAACACTAAGAAAGCCATGGCATGGGATGAACCAAATGCAAGTTGTGGGCGCAGTTGGCTTCCAGGACCGACGGCTTGACATTCCAAAAGAAGTAGATCCTATAGTTGCATCAATTATACGTGATTGCTGGCAGAAGGATCCAAACTTGCGTCCTTCTTTCATCCAATTAACTAGCTACCTGAAGACATTGCAAAGGCTTGTAATCCCTTCACATCAGGAGACAGCGAGCAACCATGTACCCTATGAAATATCTTTATATCGGTGAACCGCACATCTCTACCCCGGGGTTGTCACCCACCAAGTGTGAATAAGCAGTAATTATGATTTTGTCATCGAAAAAAAAAAAAAAAAAAAAAAAAAAAAAA |
| TaNAC21/22 (1553 bp) | >KY461027.1 Triticum aestivum NAC domain-containing protein 21/22-like protein (NAC21/22L) mRNA, complete cds  CTCTAGCATCACACAAGGTTCAAGCTACAGCAACATAGATATATGCCCTGCCTAATTCCTGCTACCTACTCTACATACCTAGCTTCTTCTCTCCTCTCTGGAGATCTTGTCAATCTCTCTCTCCTCCGTCTAGCTAGCTAGAGCTAGCTCAATTGGTGCCTTTAGGCAACGAAGTTCATCGAGATCTCTCATCCATACCCGTAGTACACATCGCAATTCACAATTCAAATTGTAGACAGAGTCGAGCTGAGCTGAGCAGGGGGAGGTTTCAGAGAGAAGATAGTGCACAGATAGAGATAATAAGCAATTCCATGGGGCTGAGGGACATCGAGATGACGCTGCCGCCGGGGTTCCGCTTCTACCCGAGCGACGAGGAGCTGGTGTGCCACTACCTGCACGGCAAGGTGGCCAACCGGCGCCTCTCCGGAGGAGCCGCCGGGACCATGGTGGAGGTCGATCTGCACGTCCACGAGCCATGGGAGCTGCCAGACGTGGCGAAGCTGAGCACGAACGAGTGGTACTTCTTCAGCTTCCGCGACCGCAAGTACGCGACGGGGCTGCGCACGAACCGCGCCACCAGGTCCGGCTACTGGAAGGCCACAGGCAAGGACCGCGTCATCCGCAGCCCGAGGTCGTCGTCATCCCGCTCTGGCCGCGCCGTCATCGTCGGCATGCGCAAGACCCTCGTCTTCTACCGCGGCCGCGCCCCCAACGGCAGTAAAACCTGCTGGGTCATGCACGAGTTCCGCATCGAAAACCCTCACGCCCCACCCAAGGAGGACTGGGTGTTGTGCAGAGTTTTCCACAAGAAGAAAGCTGACACGGAGTACGCCATGGACGGCGAGCAAGAGATCGTCGGGGGCATGGCTCGCAGCGCTGCTGCCGTGAGTGGATCCTGCCACGATCCGGATCAGTACCACCACTCGCCTCCGGCGGCGCTGTTCCCCTCCCTCGGCGCAGGCGGTCACCCTTACCAGCTCACGTCCTGTGATCATCACCATACCCACGGCGCCGCCGGCGTCTCGCTCACTGACGTGGACCCCTTCGTGGACATGCCACAGCTGCTGAGCTACGACAGCATCCTCGACTTCAACCAGCAACTTCAGGGCGGTGGCGCAGCGGCGGGTTTGAGAGACGGCGCCGGGGATCAGTGTGGCGGGGAGCTGATGGACCTAGGGCTCCAGGTGCAGGAGGAGCACTACAACTACAACAGCTTGATGTAGATGTGAATATATGATATGGATCTGTGGGTGTTGCATGCTGCTATTGCTCGGCCATACACAGTAGTGTGTATTGGCCATCATTATTCGTTTGTGATCTTGTGCAGATTTCGTTATTTGTGCATGTAGTGTAGGGTAGAAGCATAATACATACTATATACATACTATTGCAATGGGTTATATAATTGATCTCGATTGATTAGGATATGTGAGAGTGTGAGGCTGGTGTGTCATGTACATATGTACTGTATGGATCATTTCTGTTGTTGGAGAATGGTGACAAGTTGGGGCATTTGATGTTGTTGTTCCTATACCTGTAATGACTAATAACTA |
| TaABCC6 (5236 bp) | >2A dna: chromosome chromosome: IWGSC:2A: 700979382:700990452:1 (TaABCC6)  ACTGCCTCGCTTCAAGCGAGAGATAGGCGCGCCCTTCCAGGGGAGCTCCTTTTTGCTGCATTCCAGTTTGCTAAAAAAATGCCGCATTCCAGAAGGTTCCCTGAACGAGTTCTGTAGGCTGTAGCATGTCTTATATCTATCAAAATGGGCCGGCTCAACGCAGTTTGATCGATCTGTATCCTGTGCGATACAGCGGCAATCTGCTGCAAAATGCGGCAGATAGGGTTTAATATTAAGGCAGTCCAGCACTGCAATCTCGACTTGGACATCTAGCCAACAATTTTCCCCAAAAGCAAGTCTGGCCAATCATTGATTCCCAGCAAGTAGAGGTCAAGAGAAAACGACAATGCTTATAGAACAGCCATAAACTGCAGAAAAAAGAGCATGCATTGACTGACCATGAAGAGTTGATGGAGTAGCAACCAAGGTACCACGTTCGTCCTTACGTATTATACATACAACGCAAACGCCCTTCTCCAATAGAAAATGGAAGCATTATTGTGTAATCATTGAGGAGACTAGACTTCACTTGCGCCGTCGCCATAATGTCAAAAACGCTCTTATATTATGGGTCTGAGGGAGTATCTTCTTGCCCCACCTGGCTCTCGCTCGCTTAGTTTTAATCTGACCTGATCACGGCCAAGGCCTCGTGCGTCATTCATCCACGTTAGGTTAGGTGGCGTCCAACGTCATGTGCTTGGATCAGCTGCCTTAATTTGATATGCAATTCTCTGTCAGTTTGTCGCCTCGTTGATTCATTTTGAATTCTCTGCTCAGCATTTTTGTTGTTGTTGCACTCTCTAACCTTGCCTGCCCAAGTGCCCGTCACCATCGGGTCCTCGGGTCGCTCCACCTCCGATATATATCTCACCGTCGATCACCACCCCTCAACTCTGTCTCTCACTCGCATTTGGCCTCACCGCGCCATGGAGGGAGGGAAGAGCCAGCTGCACTCGCCTCTTCTTCTTCTTCTTCCCCAGAGGATGCAGGAGATGTTCCTCGCCCACACGGCGGGCTTGCTCCATGATTCGCCGAGTAAGTCTTGACAAATTTCAGGTCCTGTTAATAAGGGTCTGCTTGGTTTTCTTTTTGTATTCCGACATGGAGCAGAAACCGCACAAGAATGTGCATACAAACATGGCAGCATTTGTAGCCCATGACCTGTAAATTTAGTTAGTTAGCCTCGATGGTTTTGTCCTTTTGTATAACTAACTAACTGAAGTAATAGCCACAAAGTTCAAGATTTCAGTGACCAAGAAATAGTTTTATAAATTTACTTATGGTTTCAAACATGGTCATGAAGAAAATTTAGCAAACTGGGCCGATGACAGTCAGATCATGTGCAATGATCAGCTTTCCGTTTTCATCCTAGCATCCTTTCTGCAAAAATATAATATCACTCTGACTAACAAACACTCCTATGCATACAACTGTCTAAAAGAAACCAAGGGCTTGCTGTCAAAAAAAACAAGGGCTAGTATATCTTTTCTAGACTTTTTCCCTTCAAGTATTAGTCATTCAATCTTTAAGAAATAAGGAATATGTCAATACAGGGAACACTAGGAGAATGTATGTATGTTTTTTCTTTCTCAATTAAGAAGAGGAAAGAGTGTAAAATAACCCCCTCAAAAACACATCAATTACTATGCTTCCATATGCACCAAGCTACCAACACAATCAAAGTGTCAAAACACTTGGCATTTGAGAAAAAGGGTTTCCCCCCGCTTTGTATTCCAAAGCAACTATCCGATACAGCCAACGATAGGTGCTGGGGCGGAAACAGCACAAGCACGCCCAAAAGAAAGAAAGAGAAAAAGAAAAGAAACAAATGCCGATAACGGTGGATCGACAAAAATGACGAAGCCTCGCAACCGCTGCGTCCACCGGAGATCGCCCACCAAGCTCCGAGACTCCGAAGCGCCGGTACCAATCAACACCTCCAAGAAGGGACGCGACGATGGCGACGCTGCTGCTAAGGGTTTCCCCCGGTACACGGCGAGGAGAGAGGAAGGGTAGCCCCGACGCCCTCTAGGAAGGTCCGAGGGCACCCTCAGGCGCCACCGCGTCAGTGTCGGCCAAGCCAACAGAGATTTCTCCCGATCCCATCCTCCACCTCAGGCACTCCGGAGCTCGCCACCAAACCGACCACCACCCTACGCCAACACGGACACGAAGCTTCCCGCGCTGTCTCACCACGGCACCGCGAAGATGGACTGCACAACGAAGAAGAGGAGCCGGGACTAGGGCAGCAGCACCGTCAGCATACGGGAGGGCCCCACCTCCACCGTCCATGACGGTAGCCGACCGGGCGCCAAAGCAGGAGCCTACTGGGCCCGTGGCCCCACAGGCCCGGCCGGGCCCTCAGAGGCCCGGACAGCTCCCGCCTCCACGCAGCAGCTGGCACGCCGTCGACGCCGCTGCCCCAGTCCAAGCCGCTCCCCACCCTCTCCATACGGAGAGCGTCGCGGTCGCGCCGGAGCCGACCCGCAAGGACCCAGATGGAGCCCTACGGGCCCAGATCTGGGCCGGGATGCGCCACCGGCCATCCAGCACCACCGGAGCGCCCCGCCGCCAAGAGGCGGCACCACCGCGGCGAGCGCCGCCGGCCTCCGCCACCAGGACGCCGGGCCGCCACCAGCCGAGCACCGCCGCCGCCTCCCCCCACCCCGGGAAGGAGGAGAGCGCGCGGGTAAGGGACGACCCGTCGCCGCCGACACCACCCGGCCGGACNNNNNNNNNNNNNNNNNNNNNNNNNNNNNNNNNNNNNNNNNNNNNNNNNNNNNNNNNNNNNNNNNNNNNNNNNNNNNNNNNNNNNNNNNNNNNNNNNNNNNNNNNNNNNNNNNNNNNNNNNNNNNNNNNNNNNNNNNNNNNNNNNNNNNNNNNNNNNNNNNNNNNNNNNNNNNNNNNNNNNNNNNNNNNNNNNNNNNNNNNNNNNNNNNNNNNNNNNNNNNNNNNNNNNNNNNNNNNNNNNNNNNNNNNNNNNNNNNNNNNNNNNNNNNNNNNNNNNNNNNNNNNNNNNNNNNNNNNNNNNNNNNNNNNNNNNNNNNNNNNNNNNNNNNNNNNNNNNCGGCCGGACCCGGGGGCGACCGCCAGCGGCGGCGGGGGGAAAGGGCGGAGGAGGACGGACGAGAGGGAGGTGAGCGTGCGCCGCCCCGGCCGCCTCCCGGGGAGACGGCGGAGGCGGTGGGACGGGGAGGAGGGGATGGGGGGCGGGGAGGGGAGGCCTGCGCCGGGCGGCTGGCGGCGGCGGGAAGGAGCCGGGCGGCCGGCGCGGCGGCGGCGGCGGTGGGAAACCCTAGCGGGGGAGCGGGAGCGGGGTGGGAGCGGCCGTCTCAGCTCTCCCACGCTTTCAAACACTTGGCATTTACCTATCAACTTGCCGCTTTGCTTGGCGCCTTGCTTCTGTTCTACCTCTTTGCCATTCATGGCTTGAGTTCGGGCAACCCAAGTGGTGTGTCATTTGTTGTAGTGGTGCTTGCTGGGAATTAGCACACTCTTAACGGTAATCTGAAACACTGCAGTGGAAATGATGTAACATCAGCACCACCTCTATGCATTTACCACATGGATCGTTGCTCAGTCACTGAAGTCAAAACTTAGGATGATATGGAATACATTCTGTCAGCCAAAACTTAATACTTTTTTGCAAGTTTCTGAGGTGGAGAGACAGAGCCATAAGTTCCTACCAACATGACTTGTGTTTTTAGCCTCGGGTTGCCATTGCTGGTAGAACTAGCTTGGAGAATGTACATATATAAACTCCAATTTTTCCAAAAGTCGACTAATGTCTTATTCAGGAAATTCCATTTTCATAAAGAAAAAACATGTTGTTTTCTGTTTTCTCGAGCATTGATGACGTAGATGTAGCTACTATTCATAATAAAAATTGGATAACCGATTCTGCTGTTGATTCTTGACACACAAATTTGCTATGCAACCTTGCAGATTCCATTATATCGCAACACATGCAAGAATGGCCAGAAATATATTCACCTTGTTTCTGGACGAGCACTTTTGTATATATACAGCTGGTATTTATCACGAGTATCATGGCTCAGTTTCTGTTCAAGCGAATCAGATGCTGCAGGCAGAGATTGAAGACTGCAACTCCTGAAAGCAATAAGCATTCTAATCAGGAACAAAACAATGCAGACATAAAGCTGGGTCTCTCATATCAAGCAAGCAAAGTCTGTTGTCTGCTTATATTAGCCACTCATGTCCTGAGGATATTCTTTTTGCAGTTACAAGGAAGAATAAGTGGTTGCAAGTACCCACCTTTTGTTCTGGGTGAAGGCATACAGGTGCTCTCCTGGATATTATTGTCACTAGCAGTATTCAGTCTACAGAAGACAAAATCTGCAAAGCATCCGCTCATTATTCGGGCATGGTTGGTACTTAGCTTTCTGCAATCAATAATCAGTGTGATATTTGATCTCAGATTCAGCTTATCAGATCATGGATACATGGGGTTTGCGGAATTGATGGACCTGTTCACACTTGTTATCTGCACTTATCTGTTTGCAATTTCTGTCAGAGGAAAAACAGGAATCACCTTAATAAACAGCAGCATAACAGAGCCACTATTGAGTCCATCTGCAGGACAGCAGACAGAAACCAAAAGAACAAGTCTGTATGGCAAAGCAAGCGTTCTGGACCTTGTCACATTCTCCTGGATGACTCCTCTATTTGTTATCGGATATAAGAAACCTCTAGATAAGAATGATGTGCCAGATATTGATGAAAGGGACTATGCGGATTTACTCTCTGATTCATTTAAAAGGATCCTAGCAGATGTTGAACACAGGCATGGTTTAAGTACTTTATCAATCTATAGAGCAATGTTCCTATTTATTAGAAGAAAAGCAACACTCAATGCAGTATTTGCAATTCTGTGTGCATGTGCATCCTATGTTGGACCATCACTGATTAATGACTTGGTGAAATTCCTTGGGGGGGAGAGGAAAAATGGACTGCAAAAAGGTTATCTTCTTGCTGTTGCATTTTTAGGTGCCAAAGTTGTGGAGACAATAGCAGAGAGGCAGTGGATTTTTGGAGCTCAAAGGCTCGGGATGCGGCTACGAGCTGCTTTGATATCTCACATCTACCAAAAGGGGCTCCGGTTATCCTGCGGCGCAAGGCAGAAGCATTCCAGTGGAGAGATCATAAACTACATGAGTGTAGATATACAAAGGATAACCGAAGTTATGTGGTACACAAACTACATTTGGATGTTACCCATACAGCTTTCTCTAGCAGTCTATGTTCTCCATCTAAACCTAGGTGCTGGAGCATGGGCTGGTTTAGCAGCAACACTCGCAATAATGACTTGCAATATTCCTCTGACCAGACTGCAGAAAAGGTTGCAATCAGAGATCATGGCTGCTAAAGACAACAGAATGAAGGCAACAACGGAAGTGCTTAGAAGCATGAAAATACTGAAACTTCAAGCATGGGATACAGAGTACCTTCAAAAGCTAGAAGCTTTACGAAGGGAGGAGCACAATTGGTTGTGGAAATCTGTAAGGTTGTCAGCTGTAACAACATTCATATTTTGGGGGTCCCCTGCATTCATATCCTCCATAACATTCGGTACATGTATATTGATGGGGATTCCTCTAACAGCTGGTACTGTTTTGTCTGCTCTTGCAACGTTCCGGATGCTACAAGATCCAATCTTCACACTCCCTGATTTACTTTCGGTGTTTGCTCAGGGGAAAGTTTCAGCTGATCGAGTAGCACAATACCTCCAGGAAGAAGAATTGAAAGACGACGCAATTACAGAAGTACCAAGGAGTGACACAGACTTTGATGTGGAGATTGATCATGGAGCATTCAGCTGGGAACCTGAGACCACATCTCCAACTATAACAGATGTAAATTTAAAAGTAAAGAGAGGGATGAAAGTAGCAATCTGTGGAGTAGTTGGCTCTGGGAAATCCAGTCTATTATCATGCATACTTGGGGAGATGCCTAAGCTAGCTGGGACTGTGAGGGTCAGTGGGAGCAGAGCATATGTTCCTCAGACTGCCTGGATCCTATCTGGGAACATCAGAGACAACATTCTGTTTGGAAACCCATATGACAGGGAAAAGTACCAAAAGGTAATACAAGCTTGTGCATTGACAAAAGATCTTGAGCTATTTGCAAATGGCGATTTGACGGAGATTGGAGAAAGAGGAATTAACATGAGTGGTGGACAGAAGCAGAGGATTCAGATTGCAAGGTCAGTGTACGAGGATGCAGATATATACCTCTTTGATGATCCTTTCAGTGCAGTAGATGCTCACACTGGAGGACAACTTTTCAAGGTTTGTTTATTCAGTGTCCATAAACCCATAAAATGTGTTTATCTATGTTCGTATGCTGTTAACTTGACAATTTATTTCCAAAATTTAGTCGCATATGATTTAAACAGGATAAATATCACCAGTTTTGCTTATTCTTCAACGGTTTAGAGGCATCAGTCTGCATGAATGCTAATAGAATGAACTACTATATTATTCTGCAGGATTGCCTCATGGGGATGCTTAAAGACAAAACAATATTGTATGTGACACATCAAGTTGAATTTCTTCCAGCCGCAGATCTTATACTAGTAAGTGCAAAAAAAAAGTTTTCGCCCGCAAATAATGCATACTCCCTCCGTCCCAAAATAAGTGTCTCAACTTTGTACTAACTTTAGTACAAAGTTGTACTCCCTCCGTTCCTAAATATAAGTCTTTGGAGACATTCCACTATGGACCACATACGGAGTAAAATGAATGAACTACACTCTAAAATGCATCTATATACATCCGTATGTGGTCCATAGTGAAATCTCTACAAAGACTTATATTTAGGAACGGAGGGAGTACTAAAGTTGAGACACTTATTTTGGGACGGAGGGAGTATACGTTAGCATTGCTAAGATATGCAAAATAACCAAAGGGATACAAAATAATAGGTGATGCAGAATGGGAAGATTGTGCAGAAAGGAACATTTGATGATCTCCTTCAACAGAACATAGGATTTGAAGCCATAGTCGGAGCCCATAGCCAGGCAACTGAGTCTGTCATAAATGCCGAGAGTTCCAGCAGAATTTTGTCAACAGAGAACCAAAAGTTAGCAGATAGTGATGATGAGTTTGAGAGAGAAAACCACATTGATGATCAAGTTGAGGGTATAATTAAGCAAGAGTCTGCACACGATGTCTCACAAGGTATCAATGAAAAAGGAAGGCTAACACAAGATGAGGAACGAGAAAAGGGAGGGATTGGCAAGACGATCTACTGGGCATACCTGACGGCTGTTCATGGTGGCGCATTAGCACCGATAATAGTAGCAGCACAGTCATTCTTCCAAATATTCCAGGTCGCAAGCAACTATTGGATGGCATGGGCGTGTCCTCCAACGTCTGCAACCACCCCAAGGGTTGGATTAGGCCTTCTTTTCTTCGTATACATAGTGCTATCTATAGGAAGTGCACTATGTGTTTTTGGTCGGTCTATGCTTGTTTCGCTTGTTGGCCTGCTAACAGCGGAGAAGTTCTTCAAGAATATGCTCCATTGCATCCTCCGTGCTCCAATGTCCTTCTTTGATTCCACACCCACTGGCAGGATCCTAAACAGGGTTGGTGTTCGATTACTCCTCACATCATTTTGCACTGGCTAGTCCTGCTATTTCCCTCAAAAAAAAATTTGCACTGTCTCCTTGAAATTAATAATTGTGTACTGTACTTTGTAACAGGTCTCCAATGACCAAAGTGTCTTAGATCTGAAAATGGCAGACAGCCTAGGTTGGTGCGCGTTTTCTTTTATACAAATTCTGGGGACCATTGGCGTTATGTCACAAGTTGCGTGGCCGGTTTTTGTCATCTTTATTCCAGTGACAGCAATCTGTTATGTGTTTCAAGTAAGAGCTCCTTGCAGTGATATTTTTGCACTAGATTTAACTCGTCCGAGTACAATGTATGCATCAAGTTATTTCCGGGAAGCCATTAGTGCATATTTCCTCCTTGATGATTTTTCACAAAGCACTGAATCCAATATTCTCCCTATGACATGCAGCGGTACTACATACCAACAGCAAGAGAGCTGGCTCGTCTGCAACAAATTCAAAGGGCTCCAATACTCCACCATTCTGCGGAATCACTTACAGGAGCGGCAAGTATTAGAGCATATGGACGGAAAGACCGCTTCAGCAAAGCAAACATCAGTCTTGTTAACAACCACTTACAACCATGGTTCCATAACGTCTCGGCTGTGGAGTGGCTTTGCTTCAGGCTAAACATGCTATCTAACTTTGTCTTTGCCTTTTCTTTGACTCTGTTGGTGAGTCTTCCCGAAGGATTTATAAATCCAAGTAAGTTTTTCATGCAGACAAGTTATAGTTGATTCATTTTTGTAGTCTATACTCTGATCACTAATTTTTGGTGGCTGTGAAACTGGCATAATTGTATGCACAGGCATTGCGGGACTTGCAGTGACTTATGCGTTGAACCTCAACGGGCAGTTGTCATCTGTAACCTGGAACATTTGCAACACAGAGAATAAAATGATTTCAGTTGAAAGAATAATGCAGTACTCAAGGATCCCTAGTGAGGCTCCTCTAATAGTAGATGATCACCGCCCCCCAAACAGCTGGCCAAAGGATGGTACTATAAATATAAGAAACTTGGAGGTATGCATGATGTTACTGCGAGTTATCACTTATAGTAACACAAAAGGAAAGCTCTTACTTTTGAATAATGAAATGTAATCTCTTTTATTTTTCAATTTTCTCATAAAATATGTGCATGCTCAAATGAAGCCACTAATGCGACTATTTGCTTTTACAGGTTCGATATGCAGAGCATCTCCCCTCTGTTTTAAGAAATATATCATGTACAATTCCAGGACGGAAGAAGGTGGGGATTGTTGGACGTACTGGCAGTGGAAAGTCAACTTTGATTCAATCGCTTTTCCGGATTGTTGAACCGAGACAAGGGACAATTGAAATTGATAATGTTGATCTCAGCAAAATCGGGTTGCATGATTTACGAGGCAGACTTAGCATCATCCCACAGGATCCAACCATGTTTGAGGGCACAGTGAGAGGAAATCTTGATCCACTAAATGAATATTCTGATCAACATGTATGGGAGGTAAATAATTCAATTATACATGTTCTAATGATAAGAGAGTAAATTATATATAATGTGCAGCAAAAGGGAGCCTAACACCTCATATTATTGTTCTTTATTGCCAATAGTCCATGTTACAAAACAGATAGTTCTGGATAATTGTACCTTTTTCAACAAAAATATTGCTAACAGTTTATGCTGATAAAATATGATCTCATGATTATGCTAATCTATGAAAGGTCAAAATTGCTGACAGAGTATCCTAGGAAAAATATTTGGATGGTCATTTTTACTATTGCAAATACCGAAGATGATGTAATGTTTCATACAGATTGCAACCTGATCAAATCCTTCTTCAGATATATCTATATATATAATGAATAAAAGAACAGGAGATATTGTAAAAAAAATGTAATGATTAAAAGAATGGAACACTGTACTTCCTCTACTGACCAGATGTATACATTTATTTTGGCCAGACATTGGACAAATGCCAGCTTGGTGACATAGTTCGTCAAAGCCCAAAGAAGCTGGACTCAACAGGTTTGTTTTTGTGTACAACAGCACACATATGCTAGACTGTCTAACAGAGTGTGTATATCTCCATTGAGATGATCAAATGAGATTTCTCCTGCAGTTGTCGGAAATGGGGAAAACTGGAGTGTCGGACAGAGGCAGTTGTTTTGCCTGGGAAGGGTTCTACTGAAGCGAAGCAATGTCCTTGTCCCCGACGAGGCAACTGCTTCAGTTGACTCATCTACGGATGCAATTATCCAGCAAACACTCCGTGAGGAGTTTGGGGACTGCACGGTGCTGACAGTAGCACATAGAATTCACACAGTTATCGACAGTGATCTTATTCTTGTCTTCAGCGAAGGTAAGCCGCACCATTGACTAACAAAAAATATTTATCCACATTTTAGGACCTAGTGCCGCATCGCTAAAATCTGTACTCTTTTGTGCTGAGCAGGAAGAATTATAGAATACGACACGCCATCAAGATTACTGGAGGACAAAAACTCTGAATTTTTGAGGCTCATAAAGGAGTACTCACAGAGATCCAAGGGGTTTTGACAGCACCAGAAACAATTGAACGGCTGGCGAATCCGACATAATGCCAACTAGGCGCTCTGCAGAACTTAGTAGAAAGGCCACACACACACCACACGGACTTTCTTCAGATGACAAGCGCGCGCAGAACCCACCTAGAAAGAGACGATGGAATTTACAATGGGATGGGGCTAGCTAGATTATTCAGGCGATTCGGAAAAATTATTAAGTCGTGCAATGTACTAGTACTAGTACACTAAGCAGTCTAGCACTATGTAAGCTGCTCTCTGATGTGAGAAAAATAAGAGTGTGTAAGCGATCCCGCGTGAGCTCTTAAATTTCCTCGAGAGAACGTTGTCGCAGCAGACTAGCAGAGGAGTCCCGCGTAATCGATTGTCAGTTAAAGAAGTAACTGGACAGAGTTCGCGGCGGGGTACTTACATGCGGCGTATCCAGCCAGGCATTCCGTCGAGCAGGTAGTGAGCCGAGTCCTGCACAAGGAGTTGGGCATGGTCTGACGAGAGTATACCCGATCTGAGCGCAAGCATCACACCGAAGCCGCTGATTTGGGGCACTCGCTACCTTGCTGGGATTTCGAGCGGTGGCGGTGTGCCGCCGGCGTCGTGGACCGTGGTGCTTGGTGGTACGGCTTGTTTTCTCGTTCAGTCCACGGTTCGGCAAAGCTAAACGTTCGAACCCCACCGGTCGGTATGCAATAGCTATAAAATCGGCCCAACAAACAGTTCTGGAAGTTCAGACCGGTTTTAGAACCTTCCGAGTTTTTTTTTATTTTTCTGTGTTTCTCTATATTCTTTTGTTTTTTATTTGGGAAACACTTATCATCACCCGGCGGATCACGGCGGCCGCATCCGCCACCTCCCCTGCGTGACACGCATCCAAGTCAGGCGCCGCATACGATC |
| TaNFXL1 (632 bp) | >HQ595070.1 Triticum aestivum homoeologue 3 NFXL1 (NFXL1) gene, partial cds  CTTCCTTGCCGCGAGACCATATTCAATGATCTCTCTTGTGCGTGTGGCAGGTCATCTATTCCGCCACCGCAGCCTTGCGGCACACCAACTCCATCATGCCCACACCAGTGCATTGTCCCTCAGCCTTGTGGACACCCAGCTTCGCACCAATGCCATTTTGGGGACTGCCCACCTTGCGTTGTGCCAGTCATGAGAGAGTGCGTTGGGGGACATGTGATGTTAAGGAACATCCCTTGTGGTTCAAAGGATATCAGATGCAACCAGCCCTGTGGAAAGAACCGTCAATGTGGACTGCATGCTTGTGCCAGGCCTTGCCATCCTTCCCCTTGTGATCCATCGCCCACAAATGGAGAAGCTAGTTCAAGCTCCGGGGGCAAAGTTTCATGTGGACAATTGTGTGGTGTCCCAAGGAGGGAATGCAAGCACACTTGCAACGCCCCATGCCACCCATCGTCGCCTTGCCCAGACGTGAGATGTGAACATCGTGCGACTATTACCTGTTCTTGTGGCCGTATCAGTACAACTGTACCCTGCAGTGCTGGTGGAGCCTACAATGGTGATAGTACGTTTGATATCTCCGTCATCCAGCAGCTGCCAATGCCTCTCCAACCAGTGGAATCAAATGGGAAAAG |
| TansLTP9.4 (771 bp) | >AJ784900.2 Triticum aestivum mRNA for type 1 non-specific lipid transfer protein precursor (ltp9.4 gene) (TansLTP9.4)  GCTTAACCACACTCATCTGATCACCCATAGCTAGCTAGCTAGCAGCTTAAGGAACTAGCTTGCAGCTACCGATCGATGGCTCGTCTCAACAGCAAGGCTGTGGTGGCCGCCGTGGTCCTGGCGGCGGTGGTGCTGATGATGGCCGGCAGGGAGGCCTCGGCGGCGCTGTCGTGCGGGCAGGTGGACTCCAAGCTCGCGCCGTGCGTGGCGTACGTAACGGGGAGGGCGTCCTCGATCAGCAAGGAGTGCTGCTCCGGCGTGCAGGGGCTGAACGGCATGGCCCGCAGCAGCTCGGACCGCAAGATAGCGTGCAGGTGCCTCAAGAGCCTCGCCACCAGCATCAAGTCCATCAACATGGGCAAGGTCTCCGGCGTGCCCGGCAAGTGCGGCGTCAGTGTGCCCTTCCCCATCAGCATGTCCACCAACTGCGACACTGTCAACTAGTTCAATATATAACCCTTCCTACGTGCATGCATGGACGCGCTTGTGTGGAGCTTAATGCCTATGTTGATGGAGTGCTCATACGATACTGATGAGCTACTAAAAAAATAAAATAAAGTTGCCTGCATGTGTTATTTCAGTAGTGTGTCTATGTCTGTACCTTGCACACACACTGTGTGTGCATATATATTTATGCGACATGTATGCACACGTCCGTATGCGTGAGTGAACTGTCAGTTCCTTGCTGTACTCCAGTCTAGCCGAGATGTGTACGTGTGATGTGCCTGACCTACTTGTTCAGGCAATTAATGAATAGTAATTATTTTCCTA |
| TaSTP13 (1548 bp, 1545 bp, 1545 bp) | >KR604814.1 Triticum aestivum 4A hexose transporter mRNA (TaSTP13-4A), complete cds  ATGCCGGGCGGAGGGTTCGCCGTGTCGGCGCCTTCCGGCGTGGAGTTCGAGGCCAAGATCACGCCCATCGTCATCATCTCCTGCATCATGGCGGCCACCGGCGGCCTCATGTTCGGCTACGACGTCGGCATCTCAGGCGGAGTGACGTCGATGGACGATTTCCTGCGCGAGTTCTTCCCGGCGGTGCTGCGCCGGAAGAACCAGGACAAGGAGAGCAATTACTGCAAGTACGACAACCAGGGCCTGCAGCTCTTCACGTCGTCGCTTTACCTCGCCGGCCTCACCGCCACCTTCTTCGCCTCCTACACCACCCGCCGCCTCGGCCGCCGCCTCACCATGCTCATCGCCGGCGTCTTTTTCATCATCGGCGTCATCTTCAACGGGGCCGCACAGAACCTCGCCATGCTCATCATCGGCAGGATCCTGCTCGGCTGCGGCGTCGGCTTCGCCAACCAGGCCGTTCCCCTGTTCCTGTCGGAGATCGCGCCGACGAGGATCCGCGGCGGGCTCAACATCCTGTTCCAGCTGAACGTGACCATCGGCATCCTGTTCGCTAACCTGGTGAACTACGGCACGAGCAAGATCCACCCGTGGGGCTGGCGGCTGTCGCTGTCGCTGGCCGGCATCCCGGCGGCGATGCTCACCCTGGGCGCGCTCTTCGTCACCGACACCCCCAACAGCCTCATCGAGCGCGGCCACCTGGAGGAGGGCAGGGCGGTGCTCAAGCGCATCCGCGGCACCGACAACGTGGAGCCGGAGTTCAACGAGATCGTGGAGGCGAGCCGGATCGCGCAGGAGGTGAAGCACCCGTTCCGCAACCTGCTCCAGCGCCGGAACCGGCCCCAGCTGGTCATCGCCGTGCTCCTCCAGATCTTCCAGCAGTTCACGGGCATCAACGCCATCATGTTCTACGCCCCCGTGCTGTTCAACACGCTGGGGTTCAAGAGCGACGCGTCGCTCTACTCGGCGGTGATCACCGGCGCCGTCAACGTGCTGGCCACGCTGGTGTCGGTGTACGCCGTGGACCGCGCCGGGCGGCGCGCGCTGCTGCTGGAGGCCGGCGTGCAGATGTTCCTGTCGCAGGTGGTGATCGCCGTGGTGCTGGGCATCAAGGTGACGGACAGGTCCGACAACCTGGGCCACGGGTGGGCCATCCTGGTGGTGGTCATGGTGTGCACCTACGTGGCGTCCTTCGCCTGGTCCTGGGGCCCGCTGGGGTGGCTCATCCCGAGCGAGACGTTCCCGCTGGAGACGCGGTCGGCCGGGCAGAGCGTGACGGTGTGCGTCAACCTGCTCTTCACCTTCCTCATCGCGCAGGCGTTCCTCTCCATGCTCTGCCACCTCAAGTTCGCCATCTTCATCTTCTTCTCGGCGTGGGTGCTCGTCATGTCCGTCTTCGTGCTCTTCTTCCTCCCGGAGACCAAGAACGTGCCCATCGAGGAGATGACCGACAAGGTGTGGAAGCAGCACTGGTTCTGGAAGAGGTACATGGACGACGACGACCACCACCACCACAACATCGCCAACGGCAAGAACGCCACCGTCTGA  >KR604815.1 Triticum aestivum 4B hexose transporter mRNA (TaSTP13-4B), complete cds  ATGCCGGGCGGAGGGTTCGCTGTGTCGGCGCCGTCCGGCGTGGAGTTCGAGGCCAAGATCACGCCCATCGTCATCATCTCCTGCATCATGGCGGCCACCGGCGGCCTCATGTTCGGCTACGATGTCGGCATCTCAGGCGGAGTGACATCGATGGACGATTTCCTGCGTGAGTTCTTCCCGGCGGTGCTGCGCCGGAAGAACCAGGACAAGGAGAGCAACTACTGCAAGTACGACAACCAGGGCCTGCAGCTCTTCACCTCATCGCTCTACCTCGCCGGCCTCACCGCCACCTTCTTCGCCTCCTACACTACCCGCCGCCTCGGCCGCCGCCTCACCATGCTCATCGCCGGCGTCTTCTTCATCATCGGCGTCATCTTCAACGGGGCCGCCCAGAACCTCGCTATGCTTATCATCGGCAGGATCCTGCTCGGCTGCGGCGTCGGTTTCGCCAACCAGGCCGTTCCCCTGTTCCTGTCTGAGATCGCGCCGACGAGGATCCGCGGCGGGCTCAACATCCTGTTCCAGTTGAACGTGACCATCGGCATCCTGTTCGCAAACCTGGTCAACTACGGCACCAGCAAGATCCACCCGTGGGGCTGGCGGCTGTCGCTGTCGCTGGCTGGCATCCCGGCGGCGATGCTCACCCTGGGCGCGCTCTTCGTCACCGACACCCCCAACAGCCTCATCGAGCGCGGCCACCTGGAGGAGGGCAAGGCGGTGCTCAAGCGGATCCGCGGCACCGACAACGTGGAGCCGGAGTTCAACGAGATCGTGGAGGCAAGCCGCATCGCGCAGGAGGTGAAGCACCCGTTCCGGAACCTGCTCCAGCGCCGCAACCGCCCCCAGCTGGTCATCGCCGTGCTGCTCCAGATCTTCCAGCAGTTCACGGGGATCAACGCCATCATGTTCTACGCCCCCGTGCTGTTCAACACGCTGGGGTTCAAGAGCGACGCGTCGCTCTACTCGGCGGTGATCACGGGCGCCGTCAACGTGCTGGCCACGCTGGTGTCGGTGTACGCCGTGGACCGCGCCGGGCGGCGGGCGCTGCTGCTGGAGGCCGGCGTGCAGATGTTCCTGTCGCAGGTGGTGATCGCCGTGGTGCTGGGCATCAAGGTGACGGACAAGTCGGACAACCTGGGCCACGGGTGGGCCATCCTGGTGGTGGTCATGGTGTGCACCTACGTGGCCTCCTTCGCCTGGTCCTGGGGCCCGCTGGGGTGGCTCATCCCCAGCGAGACGTTCCCGCTGGAGACGCGGTCGGCGGGGCAGAGCGTGACGGTGTGCGTCAACCTGCTCTTCACCTTCCTCATCGCGCAGGCCTTCCTCTCCATGCTCTGCCACCTCAAGTTCGCCATCTTCATCTTCTTCTCGGCCTGGGTGCTCGTCATGTCCGTCTTCGTGCTCTTCTTCCTCCCGGAGACCAAGAACGTGCCCATCGAGGAGATGACCGACAAGGTGTGGAAGCAGCACTGGTTCTGGAAGCGCTTCATGGACGACGACGACCAACACCACAACATCGCCAACGGCAAGAACGCCACCGTCTGA  >KR604816.1 Triticum aestivum 4D hexose transporter mRNA (TaSTP13-4D), complete cds  ATGCCGGGCGGGGGGTTCGCCGTGTCGGCGCCGTCCGGCGTGGAGTTCGAGGCCAAGATCACGCCCATCGTCATCATCTCCTGCATCATGGCGGCCACCGGCGGCCTCATGTTCGGCTACGACGTCGGCATCTCAGGCGGAGTGACATCGATGGACGATTTCCTGCGTGAGTTCTTCCCGGCGGTGCTGCGCCGGAAGAACCAGGACAAGGAGAGCAACTACTGCAAGTACGACAACCAGGGCCTGCAGCTCTTCACCTCGTCGCTCTACCTCGCCGGCCTCACCGCCACCTTCTTCGCCTCCTACACCACCCGCCGCCTCGGACGCCGCCTCACCATGCTCATCGCCGGCGTCTTCTTCATCATCGGCGTCATCTTCAACGGGGCCGCCCAGAACCTCGCCATGCTCATCATCGGCAGGATCCTGCTTGGTTGCGGCGTCGGCTTCGCCAACCAGGCCGTTCCCCTGTTCCTGTCGGAAATCGCGCCGACGAGGATCCGCGGCGGGCTCAACATCCTGTTCCAGCTGAACGTGACCATCGGCATCCTGTTCGCGAACCTGGTGAACTACGGCACGAGCAAGATCCACCCGTGGGGCTGGCGGCTGTCGCTGTCGCTGGCCGGCATCCCGGCGGCGATGCTCACCCTGGGCGCGCTCTTCGTCACCGACACCCCCAACAGCCTCATCGAGCGCGGCCACCTGGAGGAGGGCAAGGCGGTGCTCAAGCGGATCCGCGGCACCGACAACGTGGAGCCGGAGTTCAACGAGATCGTGGAGGCGAGCCGCATCGCGCAGGAGGTGAAGCACCCGTTCCGGAACCTGCTCCAGCGCCGGAACCGCCCGCAGCTGGTCATCGCCGTGCTGCTCCAGATCTTCCAGCAGTTCACGGGGATCAACGCCATCATGTTCTACGCCCCCGTGCTGTTCAACACGCTCGGGTTCAAGAGCGACGCGTCGCTCTACTCGGCGGTGATCACGGGCGCCGTCAACGTGCTGGCCACGCTGGTGTCGGTGTACGCCGTGGACCGCGCCGGGCGGCGCGCGCTGCTGCTGGAGGCTGGCGTGCAGATGTTCCTGTCGCAGGTGGTGATCGCCGTGGTGCTGGGCATCAAGGTGACGGACAAGTCGGACAACCTGGGCCACGGGTGGGCCATCCTGGTGGTGGTCATGGTGTGCACCTACGTGGCCTCCTTCGCCTGGTCCTGGGGCCCGCTGGGGTGGGTCATCCCCAGCGAGACGTTCCCGCTGGAGACGCGGTCGGCGGGGCAGAGCGTGACGGTGTGCGTCAACCTGCTCTTCACCTTCCTCATCGCGCAGGCCTTCCTCTCCATGCTCTGCCACCTCAAGTTCGCCATCTTCATCTTCTTCTCGGCCTGGGTGCTCGTCATGTCCGTCTTCGTGCTCTTCTTCCTCCCGGAGACCAAGAACGTGCCCATCGAGGAGATGACCGACAAGGTGTGGAAGCAGCACTGGTTCTGGAAGAGATTCATGGACGACGACGACCACCACCACAACATCGCCAACGGCAAGAACGCCACCGTCTGA |
| TaWRKY49 (1221 bp) | >LC169122.1 Triticum aestivum TaWRKY49 mRNA for WRKY transcription factor 49, complete cds  TGGGCTCCTCGTCCAGGAACAAGAGATGGGAGATGTGCTGAGAGCTCACGACGTCGCAGCCACCGCCGACGACGACGAGGCTGGCGTTTGGCCCGGCGAGCTCGACGAGCAGCTCATACGGGAGCTCCTCAGCGATGACAGCCTCCTCGGCTCCATGGCCCCGCCCGACGACGGCTCGGAGCGCCACCGTTCGTGCGACACGGGAGGCGCCCCAGCCGCCGCGCCGTGCAACAGCGGCGGCAGAACCGCTGCCGAGCACGAGCCGTTGCCGCCGGTTTCGGCGTCGAGCATGGCGCTGTGCTCGTCGTACTCCGGCCCTACGATCAGGGACATCGAGAAGGCGCTGTGGTCCCGGCCGTACACCTCCAGCCAGCGCTACGGCTCGCTGTATTTCCGCAGGTACGGAGCGCTGGGCACAGCGCCGGAGAGCAAGCACACGACCAAGGTGAGGGGCTGCGGCGGCGGCAAGACGCCGATGGACGGGTACAGGTGGAGGAAGTACGGGCAGAAGTTCATCAAGAACAACCCCCATCCCAGGAGCTACTACAAGTGCACCAGCGCACGGTGCAGCGCCAAGAAGCACGTCGAGAAGTCCACCGACGACCCGGAGATGCTCATCGTCACCTACGAGGGGTCGCACCTCCACGGCCCGCAGACGACGACACTCCGGCGCCTCCAGCCCCCGGACGCCGCCGCGGACCTACCTGGCGCGGCGGGCGACGCCGTCGCCGGTCCGGTGATCGGCTGCAGCGTACGGCCATCATATGGAACGTCGTCCGGCGACGATGCGCGACAAGAAGGCAACGAGCCGCTGCAGGGGCGTCACGAGGGACGACGCGCCCATGGCGCCGTGCAGCGTGTGACGCCGACGGACTCCTTGGCCTCTTCCCTGCCGCATTCTGCTGCTGCCGTTGATGCAACTGTGCTTTCTTCTTCTAGCCTGGACTCACCATGGTCCCTGGAAGCGCTACTCCCCGTAGAGAGGATCTGATCGAGGTGACTAGCTAGGCCACTAGGGCGAGAGCGTCTACTATGACGTTCGGCGTACCAGGGTATAATTAGGAGTATACAACGTACACAGTAGAGATCGTCAAAAGATCATATGAACGATTGTGGTAACCATGTTGGTGGTGAGTACCTGCGGAAACATGTAGTAGCTGATTGGGTTATATTATCGGTTTACCTGCTGAAACATGTATTACCTGACCTTCTTCATGGCGG |
| TaWRKY53 (942 bp) | >KR827404.1 Triticum aestivum WRKY53 transcriptional factor mRNA, complete cds  ATGGCCGTGGACCTCATGGGCTGCTACACCCCTCGCCGCGCCGACGACCAGCTCGCCATCCAGGAGGCCGCCACCGCCGGCCTCCGCAGCCTGGAGCTCCTGGTCTCCTCCCTCTCCGGCGCGGCGCCGTCCAAGGCGCCCCAGCAGCACCCGCAGCAGCCGTTCGGCGAGATCGCCGACCAGGCCGTCTCCAAGTTCCGCAAGGTGATCTCCATCCTCGACCGCGCCGGTCACGCCCGCTTCCGCCGCGGCCCCGTCCAGTCGGCGCCGCCGCCGCCGCCTCCTCCAGCACCGGTCGCTCCACCTCCTCCCCCACCTTTGACCGTCGTGGCGCCGGTGTCGGTGGCCGCCCCGCTCCCGCAGCCGCAGAGCCTGACGCTGGACTTCACCAAGCCGAACCTGACCATGTCGGGCGCGACGTCCGTGACATCCACGTCCTTCTTCTCTTCGGTGACCGCCGGCGAGGGCAGCGTGTCCAAGGGCCGGAGCCTGGTCTCCGCCGGCAAGCCGCCGCTGTCCGGGCACAAGAGAAAGCCCTGCGCCGGCGCGCACTCCGAGGCCAACACCACCGGCAGCCGATGCCACTGCTCCAAGAGAAGGAAGAACCGCGTGAAGACGACGGTGAGGGTGCCGGCGGTGAGCGCGAAGATCGCCGACATCCCGCCGGACGAGTACTCGTGGAGGAAGTACGGCCAGAAGCCCATCAAGGGGTCCCCTTACCCACGTGGCTACTACAAGTGCAGCACAGTGCGAGGGTGCCCTGCCCGGAAGCACGTGGAGCGCGCCCTGGACGACCCGGCGATGCTGGTGGTGACTTACGAGGGCGAGCACCGCCACTCGCCGGGGCCGATGCCGATGCAGATGGCGCCGTCGCCGATGCCAATGCCGATGGGCGCTCCCGTAGCCGTAGCTAGTGTGTCCGCCGGCAACGGGCACGTCTGA |
| TaHRC (2057 bp) | >MK450312.1 Triticum aestivum haplotype Ning7840 histidine-rich calcium-binding protein (HRC) gene, complete cds  AGCCCAACCCGAACGCTCCGCCCATATATCGATGGGTCCGTCCCACGGGCAAGCCGCCTCCCCCAGCCCAACCCACACGGCACGCCCAAACCCAAAGCCAAGTCGCACTCCTCCTCCTCCTCCTATCCTCTCCCTCGCCGAGGCGGCGACTGGCCAGCGCCGCAGCAGCAACCGCCGCCGCCGCCGCCGCCCGCGCGTCTCCGTTCAATTCGGTGAGTCGCGACGTCCCCCTGTATTCTTCTGAAAAATTCAGTAGGTCGCTATATCTTCAATTTCCGTGGGAAAATTGTAGGCCCTCCCGGCGCCGCGGTCGCCAGGGCTCTAGATCGCGCCGCGCGGGCGTCGTAGGGGTCCAGATCCGGACGAGCAGGGCTGAGAGGACGTGCTTCTATGCGCCCTTTCGGCGTTCGATTTTCAAAACGCCGTTGCCGAAGTTCATCGCGATTCTACTACGGTTTCGCCGCCGGCCGTGGCTTTAGATCTGGCGCGCCTCGTGAATTATTACGGGGTTTTGGTGCGAAATTTCGTGGCCTCGAGAATTATTATCGGGAATTGCGGATATAGATTAGATTTCCCCTAGAGGAGTCTTTTTGGGGAGGCAAATTACCTTGACCTGAGAATTTACCCTGACCGGAATTGTGGTGGTTATTGCAGGCCAATTTGGGGCTGAGGAAAGAGGAGGAGCGACTTTTCTCTGGTCTGGTGGTCGGAAATTCAGGACAAGGTAAGAAAGTGTATAATATGTCTCCACCTACCGCAATCATTGGATCTGTTAGTGCTTATGTGTGGCTGAGTCATGCATGCGTGCGCTGTACTTGTGGTAGTGCCAGCTGCGATTATTCCTACTAGCCGCCTGGTGGCAGTCAGCCCTGGCTTGGTTTATACTGCTGGCAGAAGCCTGGATTGTAGTTCTATTGATTGATCTGGCGAAAGCTTGAACCTTTAAGCTGCTGTTTTGATATTAAGTGGATTACATGAACCAGTTTGGTACTATTTCGGTGTTAGAGCATCTCCAATAGCATGTGTATATTTGGATGTCTATATATTCATATAGACAATGGTCTAAAAAGATTCCCTCATATACACATCCAGTTTTGCATCAGAACGTCTATATACAGGGACCATGACAGGTGGGCCGTTGCTGGGGAGAGGAAGAAATCATGACTACAGCTGAGTTTAGACAACGCCTTCACATTGTCCAGGCGTGGACATTGTCGATGTCGATTTAGAGGATGTGTATATTTGGACAACCATGTAGACAAGCTGTTGGACGTCTGTTTTGGGCTCACGTCGTGCAAATGGTCGAGGACAAGAAGAAGAGACTCCTTGAGAAGAAGGAAGCTCCACTGAAATGGCAGCAGAAACTGGAAGGGGCAATTAAGGCCACTGAAGAAAAGGAGAAGAAGCTCAAGTCGAAAAAGCACAGGAGGCGAAGCTATTCTTCCTCAGAATCCGACAGTGAATCCGAGAGCGACAGTGATCGGAAACGCAGGAAGAGGAAGGACCGCAAAAGGCACAAAAAACATGGCCACTCTGACTCTGATGGTGCCAGGAGGCGCAAGCACAGGTCAAAGAGGAGGAGCTCGGACTCTAGCGATGAGAGCGACAGTGATGAATATGATAGCGAATCTGAAGAGGATCGCCGAAGGAAGAAGCACTCGCACAGGAGGAAGCATCGCCGGCACTCTTCAAGGTCAGAGTCTGATGCTTCAGATTACAGCAGCGATGATGATGAGCGGAGATCAACCAGGAAGGACCACACTAGGAGCCGCAGGCGTCGCCACCGATCCTCAGACGATGAATCTGAGGAGAAGATCAGGTTGAGGCATAGGAAGCGTCACAGATCAAGTGACGAGGACAAGCCGTCAGATTCTGACAACCATAAGCGTCACAGGAGCCGCTCTATGTCCTTGGATGACGGTGCTGCTGGCGAGCCAGACAAGATGAATGATGGCAAGGGGTCTCACAAAAGCCGGCACCACCGCCGCCACCACCATCACCATCATGATCATCGTGCGAACTCTGCTGAACCCAGTGACGGGAAGCAACTCGTGTAA |
| TaDAD2 (1120 bp) | >GU564293.1 Triticum aestivum defender against cell death 2 gene, complete cds  GGCGAGCTCGGACGCTACAAAATCTCGACAGCTTGTATGACAGTTGCTCATCCGAGGAAGTTCATGATCACCAGGTGGAGCACCAGATTGCGCAGGACGAAATCAGCAAAGGCTCTTTCCGGGGGAAGATCCTGCAGATGATGTTTAGAAATACAATGTTATGCCTGAAACCAAAGAATAATTTGCATGATGTAACAGTGGAAGTATAGTTACCTTGAATTCCTTGTTGTCCTTGTTGACTTGGATACGGAGGCAAACTGTAAAAACATTGACAGTTATATATTACATATCCTGGAGACAAGGAGGAATGAACTCAATCTGGAGGGGTAAAAGAAAGAAATTCCTACCACCAAGCACTGCAGTTCCAATGCACGACAGGACACCAGACAGGAAAGAGTTGAAGGGGAATGACCCAACTATTCCCATGTAAACGACCTGTTTTGCCACATACAAGAGCATTTCAGAAGCTTGCAAATGGCTCTGTGCTACCATACCAAGCCATGGTTTATATTGCAACTAGGGGGGACACAAGGCCAGCGAGTAAAAACATTTGCTCAGATGGAAATTGCTGAAAGAATCTGGCCAACAGTCAGTCAGCACAAACAAGTGCCCGAACCAAAGAATGTGGCTAGACGCATATCAGACACTGTTACTAGGTGCATCAGATTCTTGCAACCTGAAAGCCCACTGCTGCTGAAAGATGATGAAATCTTGTATAAAAAGCATACAAGACACATGCCACCATGGAATCAGTGCATAGTTCTAGCAGACCACCAGCTATGGCGCTATGACGCCTAATGAATATCCTAAACTCAGTATGCTACTCTTCTCCATCCCAAATCAGGGCTCGCGAGTCCCCGGAAAGCATCGAAAATTACCTGAACCAGGGCAGTTGCGACGGCGAAAACCACGTACAGGTCAATGATCTGCGGAGCACGAGAAGTCGAGAAGCACATCAGTAACCCGGCAGCCGCCAGCGGCAGAGGAAAGAAAATACGAGTACTGGGGAAAGAAAACGAAATTTACCTTGAGATTCGTGGGCGTGGCGGCGTATGCCTTGGTGAGGGACTGGATCAGGAGCTTGGCGTCCCCCGCGGGCTTCGGCATCCTTCCCGGGGGA |
| TaLSD1 (1054 bp) | >EF553327.1 Triticum aestivum LSD1-type zinc finger protein mRNA, complete cds  CCACGCGTCCGACCAAACTTATTCGTCTCGAAATCTGCACCAGATCGAGCTCAGCAACGCGAGCCGAGCCGGAGGCGTTCGTCTGCCCGGGGTGGCGCCATGCGGATCCGTCTCTTCCTCTGAACCGGGCAAGCGCGAGCCGCCTTCCTCCTCGTGCTCGACGGCGGCGACGAGCGTCGGTGGCGGCATAGCACAGTCCAGCAACGAGCAGGGTTTCATGTAGGGGAGGGCAACCTGTTCCCAGCCCTCGATTTCCACCGGAGCGTGCGAAGTCCAGTCGGCAGCAACGGCGACGCCGGTGGAGGCAGCCGCGGGCACGGCAATGCCGGTTCCTCTTGCCCCTTACCCAACCCCTCCTGTGCCCTTCACACCACCCGCTCCCAATGCAGGGGCTCAAAGCCAACTTGTCTGCTCTGGGTGCCGGAATCTGCTCATGTATCCTGCCGGCGCAACGTCGGTCTGCTGCGCAGTTTGCAGTACCGTGACCGCTGTTCCGGCTCCCGGGACTGAAATGGCGCAGCTAGTTTGCGGAGGATGCCACACTCTTCTCATGTACATACGCGGCGCCACAAGTGTACAGTGTTCTTGCTGCCACACTGTCAACCTGGCAATGGAAGCGAATCAGGTTGCGCATGTAAACTGCGGGAGTTGCCGAATGCTGCTCATGTACCAGTATGGCGCGAGGTCCGTCAAATGTGCAGTCTGCAGTTTCGTCACGTCAGTTGGGGCATCATCGGGTGCAGAGCAGAAGCCCAGCAACTGAACTCCTGCAACTTTATTTGGGAAGCAGCACCCCGGAATAAGAATGCTTTGAGTGCACATGTATAGAGTTCTTCCCAGAAGCAATCTCTTTCTCCCCTCTCCATTTCCCACCTTCGTAGGGGCTGGCGAGCTTACGGAATAAACAAGAGTTTTTGGTAATACATAGTGCTCAAAATTCGTCGAATCATCTGTGTAACTGGATCAGACAGATATATGAACCCACCATGTTTATGTTCAATCCTAGCTTCTTTTACTTGTGTTCTATATATTTCAGATTTTTTTTCTTGTGGTC |
| TaHDA6 (1377 bp) | >MH556916.1 Triticum aestivum histone deacetylase 6 protein A (TaHDA6-A) mRNA, complete cds  ATGGCGGCGTCCGGCGAGGGAGCGTCGCTGCCGTCTCCGGCAGGTGGCGAGGACAGCCGCCGCCGTCGTGTGAGCTACTTCTACGAGCCCACGATCGGCGACTACTACTACGGGCAGGGCCACCCGATGAAGCCCCACCGCATCCGCATGGCGCACTCGCTAGTTATCCACTATGGTCTGCACCGCCTGCTCGAGCTTTCACGCCCCTTCCCTGCCTCGGAAGCCGACATCAGCCGCTTCCACTCCGACGAATATGTCTCCTTCCTTGCCTCCGCGACCGGCAACCCGACCATCCTCGACCCCCGCGCCGTCAAGCGCTTCAACGTCGGGGAGGACTGCCCCGTCTTTGACGGACTCTTCCCCTTCTGCCAGGCCTCCGCCGGTGGCAGCATCGGCGCCGCCGTCAAGCTCAACCGTGGCGACGCCGACATCACCGTGAACTGGGCCGGCGGGCTCCACCACGCCAAGAAGGGCGAGGCCTCTGGGTTCTGCTATGTCAATGACATCGTCCTCGCCATTCTTGAGCTTCTCAAGTTCCACAGGCGTGTGCTATATGTAGACATTGATGTTCACCATGGAGATGGTGTGGAGGAGGCCTTCTTCACTACAAACCGAGTCATGACGGTTTCTTTCCACAAGTATGGAGACTTCTTTCCTGGAACTGGACATATAACTGATGTTGGAGCGGGTGAAGGGAAACATTATGCTGTAAATGTCCCCTTGAGTGATGGTATTGATGATGACACCTTCCGTGATCTGTTCCAATGCATCATTAAAAGAGTAATGGAGGTCTATCAGCCAGAGGTAGTTGTTCTCCAGTGTGGGGCTGACTCTTTGGCTGGAGATAGGTTAGGCTGCTTCAATCTGTCTGTGAAAGGCCATGCAGACTGTCTCCGTTTTCTCAGGTCGTTCAATATTCCTATGATGGTTTTGGGAGGTGGAGGTTACACCATCAGAAATGTTGCTCGCTGTTGGTGCTATGAGACTGCAGTTGCTGTTGGCGTTGAACCTGATAACAAGTTGCCTTATAATGACTATTACGAGTACTTTGGCCCTGACTATAATCTTCATATTCAACCAAGAATTGTGGAAAATCTGAATACTACAAAAGACTTGGAGAATATAAAGAACATGATATTGGATCATCTTTCAAAGTTAGAACATGTCCCGAACGCTCAATTCCATGAAAGACCATCAGATCCTGAAGGTCCAGAAGAGAAGGAGGAGGATATGGACAAGAGACCAGCTCAGCGCAGCAGATTATGGAGTGGAGGAGCTTATGATTCTGACACAGAAGATCCTGACAACATGAAAACTGAGGCTAACGACTTATCTGCCAACTCTATCATGAAGGATGCATCAAATGATGATTTGTAG  >MH556917.1 Triticum aestivum histone deacetylase 6 protein B (TaHDA6-B) mRNA, complete cds  ATGGCGGCGTCCGGCGAGGGAGCGTCGCTGCCGTCTCCGGCAGGTGGCGAGGACAGCCGCCGCCGTCGTGTGAGCTACTTCTACGAGCCCACGATCGGCGACTACTACTACGGGCAGGGCCACCCGATGAAGCCCCACCGCATCCGCATGGCGCACTCGCTAGTTATCCACTATGGTCTGCACCGCCTGCTCGAGCTCTCACGCCCCTTCCCTGCCTCGGAAGCCGACATCAGCCGCTTCCACTCCGACGAATATGTCTCCTTCCTTGCCTCTGCGACCGGCAACCCGACCATCCTCGACCCCCGCGCCGTCAAGCGCTTCAACGTCGGGGAGGACTGCCCCGTCTTTGACGGACTCTTCCCCTTCTGCCAGGCCTCCGCCGGTGGCAGCATCGGCGCCGCCGTCAAGCTCAACCGTGGCGACGCCGACATCACCGTGAACTGGGCTGGCGGGCTCCACCATGCCAAGAAGGGCGAGGCCTCTGGGTTCTGCTATGTCAATGACATCGTCCTCGCCATTCTTGAGCTTCTCAAGTTCCACAGGCGTGTGCTATATGTAGACATTGATGTCCACCATGGAGATGGTGTGGAGGAGGCCTTCTTCACTACAAACCGAGTCATGACGGTTTCTTTCCACAAGTATGGAGACTTCTTTCCTGGAACTGGGCATATAACTGATGTTGGAGCGGGTGAAGGGAAACATTATGCTGTAAATGTCCCCCTGAGTGATGGTATTGATGATGACACCTTCCGTGATCTGTTCCAATGCATCATTAAAAGGGTAATGGAGGTTTATCAGCCAGAGGTAGTTGTTCTCCAGTGTGGGGCTGACTCTTTGGCTGGAGATAGGTTAGGCTGCTTCAATCTGTCTGTGAAAGGCCACGCAGACTGCCTCCGTTTTCTCAGGTCATTCAATATTCCTATGATGGTTTTGGGAGGTGGAGGTTACACCATCAGAAATGTTGCTCGCTGTTGGTGCTATGAGACTGCAGTTGCTGTTGGCGTTGAACCTGATAACAAGTTGCCTTATAATGACTATTACGAGTATTTTGGCCCTGACTATAATCTTCATATTCAACCAAGAATTGTGGAAAATCTGAATACTACAAAAGACTTGGAGAATATAAAGAACATGATATTGAATCATCTTTCAAAGTTAGAACATGTCCCAAACGCTCAATTCCATGAAAGACCATCAGATCCTGAAGGTCCAGAAGAGAAGGAGGAGGATATGGACAAGAGACCAGCTCAGCGCAGCAGATTATGGAGTGGAGGAGCTTATGATTCTGACACAGAAGATCCTGACCACATGAAAACCGAGGCTAACGACTTATCTGCCAACTCTATCATGAAGGATGCATCAAATGATGATTTGTAG  >MH556918.1 Triticum aestivum histone deacetylase 6 protein D (TaHDA6-D) mRNA, complete cds  ATGGCGGCGTCCGGCGAGGGAGCGTCGCTGCCGTCTCCGGCAGGTGGCGAGGACAGCCGCCGCCGTCGTGTGAGCTACTTCTACGAGCCCACGATCGGCGACTACTACTACGGGCAGGGCCACCCGATGAAGCCCCACCGCATCCGCATGGCGCACTCGCTAGTTATCCACTATGGTCTGCACCGCCTGCTCGAGCTCTCACGCCCCTTCCCTGCCTCGGAAGCCGACATCAGCCGCTTCCACTCCGACGAATATGTCTCCTTCCTTGCCTCCGCGACCGGCAACCCGACCATCCTCGACCCCCGCGCCGTCAAGCGCTTCAACGTCGGGGAGGACTGCCCCGTCTTTGACGGACTCTTCCCCTTCTGCCAGGCCTCCGCCGGTGGCAGCATCGGCGCCGCCGTCAAGCTCAACCGTGGCGACGCCGACATCACCGTGAACTGGGCTGGCGGGCTCCACCACGCCAAGAAGGGCGAGGCCTCTGGGTTCTGCTATGTCAATGACATCGTCCTCGCCATTCTTGAGCTTCTCAAGTTCCACAGGCGTGTGCTATATGTAGACATTGATGTTCACCACGGAGATGGTGTGGAGGAGGCCTTCTTCACTACAAACCGAGTCATGACGGTTTCTTTCCACAAGTATGGAGACTTCTTTCCTGGAACTGGACATATAACTGATGTTGGAGCGGGTGAAGGGAAACATTATGCTGTAAATGTCCCCCTGAGTGATGGCATTGATGATGACACCTTCCGTGATCTGTTCCAATGCATCATTAAAAGAGTAATGGAGGTTTATCAGCCAGAGGTAGTTGTTCTCCAGTGTGGGGCTGACTCTTTGGCTGGAGATAGGTTAGGCTGCTTCAATCTGTCTGTGAAAGGCCACGCAGACTGCCTCCGTTTTCTCAGGTCATTCAATATTCCTATGATGGTTTTGGGAGGTGGAGGTTACACCATCAGAAATGTTGCTCGCTGTTGGTGCTATGAGACTGCAGTTGCTGTTGGCGTTGAACCTGATAACAAGTTGCCTTATAATGACTACTACGAGTACTTTGGCCCTGACTATAATCTTCATATTCAACCAAGAATCGTGGAAAATCTGAATACTACAAAAGACTTGGAGAATATAAAGAACATGATATTGGATCATCTTTCAAAGTTAGAACATGTCCCGAACGCTCAATTCCATGAAAGACCATCAGATCCTGAAGGTCCAGAAGAGAAGGAGGAGGATATGGACAAGAGACCAGCTCAGCGCAGCAGATTGTGGAGTGGAGGAGCTTATGATTCTGACACAGAAGATCCTGACAACATGAAAACCGAGGCTAACGACTTATCTGCCAACTCTATCATGAAGGATGCATCAAATGATGATTTGTAG |
| TaHDT701 (933 bp, 933 bp, 936 bp) | >MN295033.1 Triticum aestivum histone deacetylase HDT701 protein A mRNA, complete cds  ATGGAGTTCTGGGGCCTTGAGGTTAAGCCTAACCAGTCCCTCAAGGTTTCACCTGATGATGACCACTTCCTCCATCTCTCCCAGGGTGCCCTTGGTGAAGTCAAGAAGGATGACAAGGCTACCATGTTCGTCAAGATTGGCGACCAGAAGCTAGCCATCGGGACCCTCTCTACTGACAAGTTCCCTCAGATCCAGTTCGACCTCGTCTTTGAGAAGGAGTTTGAGCTCTCACACAATTCCAAGACGTCCAGCGTCTTCTTCTCTGGCTACAAGGTTTTCCAGCCTGCCGACGGAGATGAGATGGATTTTGATTCCGAGGAAGAGTCTGAGGAGGAGCAAGACACCATCATTCCAGCACTCACCAAGGAGAATGGCAAACCTGAAGCCAAGGAGCAGAAGAAGCAGGTTAAGATTGACACTGCTGCCCCTTCAAAGTCCAAGCCTGCTGCTAAGGATGGGGGAAAATCTAACAAGGATGATGACAGCGATGATGATGATGACAGTGATGAGGATGACAGCCAAGATGACTCTGGTGATGATGGAGCATTGATTCCCATGGAGGATGATTCTGATGATAGTGAGGGCGGTGATGATTCCTCTGATGATAGTGAGGATAGCTCTGATGAGGAGGAAGAAGAAACACCCAAGAAGCAAGAGACTGGGAAGAAGAGGGCGGCTGGAAGCGTGTTGAAGACCCCTGTTACTGACAAGAAGGCCAAGATTGCAACACCGTCAGGCCAGAAGACAGGCGACAAGAAGGGAGCTGTCCATGTGGCGACTCCTCACCCGGCCAAGAAGGCAGGCAAGACCCCGGCTACCAGCGACAAGTCGCCCAAGTCTGGAGGGTCGGTCGCGTGCAAGTCGTGCAGCAGGACATTCAACAGCGAGGGCGCTCTAGCTTCGCACTCCAAGGCAAAGCACGAGGCCAAGTAG  >MN295034.1 Triticum aestivum histone deacetylase HDT701 protein B mRNA, complete cds  ATGGAGTTCTGGGGCCTTGAGGTTAAGCCTAACCAGTCCGTCAAGGTTTCACCTGATGATGACCACTTCCTCCATCTCTCCCAGGGTGCCCTTGGCGAAGTCAAGAAGGATGACAAGGCAACCATGTTCGTCAAGATCGGCGACCAGAAGCTGGCCATCGGGACCCTCTCTACCGACAAGTTCCCTCAGATCCAGTTTGACCTCGTCTTCGAGAAGGAGTTTGAGCTGTCACACAATTCCAAGACATCCAGCGTCTTCTTCTCTGGCTACAAGGTTTTCCAGCCTGCCGAGGGAGATGAGATGGATTTTGATTCTGAGGATGACTCTGAGGAGGAGGAAGAGAAGATCATTCCAGCACTCACCAAGGAAAATGGCAAACCTGAAGCCAAGGAGCAGAAGAAGCAGGTTAAGATTGACACTGCTGCGCCTTCAAAGTCAAAGGCCGCTGCCAAGGATGTGGGAAAATCTAAGAAGGATGATGACAGCGATGATGATGATGACAGTGATGAGGATGACAGCGAAGATGACTCTGGTGATGATGGAGCATTGATTCCCATGGAGGATGATTCTGATGACAGTGAGGATGGTGATGACTCCTCTGATGATAGCGAGGATAGCTCTGATGAGGAGGAAGAAGAAACACCCAAGAAGCAAGAGACTGGGAAGAAGAGGGTGGCTGGAAGCGTGTTGAAGACCCCTGTTACTGACAAGAAGGCCAAGATTGCAACACCGTCAGGCCAGAAGACAGGTGACAAGAAGGGAGCTGTCCATGTGGCGACTCCGCACCCGGCCAAGAAGGCAGGCAAGACCCCAGCAACCAGTGAGAAGTCGCCCAAGTCTGGAGGGTCGGTCGCGTGCAAGTCGTGCAGCAAGACATTCAACAGTGAGGGCGCTCTAGCTTCGCACTCCAAGGCCAAGCATGAGGCCAAGTAG  >MN295035.1 Triticum aestivum histone deacetylase HDT701 protein D mRNA, complete cds  ATGGAGTTCTGGGGCCTTGAGGTTAAGCCTAACCAGTCCGTCAAGGTTTCACCTGATGACGAGCACTTCCTCCATCTCTCCCAGGGTGCCCTTGGTGAAGTGAAGAAGGATGACAAGGCAACCATGTTCGTCAAGATTGGCGACCAGAAGCTAGCCATCGGGACCCTCTCTACTGACAAGTTCCCTCAGATCCAGTTTGACCTCGTCTTCGAGAAGGAGTTTGAGCTCTCACACAATTCCAAGACGTCCAGCGTCTTCTTCTCTGGCTACAAGGTCTTCCAGCCTGCCGAGGGAGATGAGATGGATTTTGATTCCGAGGACGAGTCTGAGGAGGAGGAGGAAGAGAAGATCATCCCAGCACTCACCAAGGAAAACGGCAAACCTGAAGCCAAGGAGCAGAAGAAGCAGGTTAAGATTGACACTGCTGCACATTCAAAGTCAAAGGCTGCTGCCAAGGATGTGGGAAAATCTAAGAAGGATGATGACAGCGATGACGATGATGACAGTGATGAGGATGACAGCGAAGATGACTCTGGTGATGATGGAGCATTGATTCCCATGGAGGATGATTCTGATGATAGTGAGGATGGTGATGATTCCTCTGATGATAGTGAGGATAGCTCTGATGAGGAGGAAGAAGAAACACCCAAGAAGCCAGAGACTGGGAAGAAGAGGGCGGCTGGAAGCGTGTTGAAGACCCCTGTTACTGACAAGAAGGCCAAGATTGCAACACCGTCAGGCCAGAAGACAGGTGACAAGAAGGGAGCTGTCCATGTGGCGACTCCTCACCCGGCCAAGAAGGCAGGCAAGACCCCGGCTACCAGCGAGAAGTCGCCCAAGTCTGGAGGGTCGGTCGCGTGCAAGTCGTGCAGCAGGACATTCAACAGTGAGGGTGCTCTAGCTTCGCACTCGAAGGCCAAGCATGAGGCCAAGTAG |
| TaHOS15 (1671 bp, 1668 bp, 1671 bp) | >MH556919.1 Triticum aestivum WD40-repeat protein A (TaHOS15-A) mRNA, complete cds  ATGGGAGCGATAACCTCGGCGGAGCTCAACTTCCTCGTCTTCCGCTACCTACAGGAGTCCGGTTTTGTTCATGCTGCATTTACTTTAGGGTATGAAGCAGGGATACATAAAGGTGGGATAGATGGAAATGTTGTCCCTCCTGGCGCTCTTATCACTATCGTGCAGAAAGGCCTCCAGTACATAGAATTAGAAGCAAATACTGATGAAAATGATCAAGAAGTTGAGAAGGATTTTGCTCTTCTGGAACCTCTTGAAATCATCACAAAAGATGTTGAAGAGTTGCAACAGATTGTGAAGAAGAGAAAAAGGGAGAGGCTTCAAATTGATTGTGACAAGGACAAGGGGAAAGCGAAAGAGTGCATTGAGGAGCATGAACACCGTCTTGGTGGTGAACGGGAGAGAGAGCGCCATGACAAAGAAAAAGACCAAGAGATGGAAAGAGATAGAACTGAAAGAGACAAGGTGCAAGAGAAAGAGAAGGAAAGGGAAAAACAACACACAGAGCATATTGATAAGGTTAAGCATGAAGAGGATCCCCTTGCCAGTGGAGGTCCTAAACCAATGGATGTAAGTACAACTCCTCATGAAATTTTTAGTGCGGATGTAACCGTTTTGGAAGGACACAGTTCAGAGGTTTTTGCTTGTGCATGGAGTCCAGCTGGTTCTCTTCTAGCTTCTGGGTCAGGAGACTCAACTGCTAGAATTTGGACAATTCCTGATGGTCCATGTGGTTCCATTCAATCCTCTCCTGCAAGTGGTCATGTTTTGAAACATTTTAAGGGTAGGACCAACGAGAAGAGCAAGGATGTCACCACACTTGACTGGAATGGGGAAGGAACACTATTAGCCACGGGCTCCTATGATGGCCAAGCAAGAATATGGAGTAGGGACGGAGAGTTAAAGCAGACTTTATTCAAACACAAGGGACCTATATTTTCTTTAAAATGGAATAGGAAAGGTGATTTTCTTCTAAGTGGCAGCGTAGACAAAACTGCTATTGTCTGGGATACAAAGACATGGGAGTGCAAGCAGCAATTTGAATTTCATTCAGCTCCAACACTAGATGTTGATTGGCGAAACAACAACTCTTTTGCAACATGCTCAACTGATAACATGATCTATGTTTGCAAGATTGGGGATCCACGCCCAGTTAAAACATTCAGTGGCCATCAAAGTGAAGTTAATGCTATCAAGTGGGATCCAACTGGTTCTTTGTTGGCTTCTTGTTCTGATGATTGGACTGCAAAGATATGGAGTGTGAAGCAGGACAAATGTGTATATGATTTTAAAGAGCATACCAAGGAAATATACACTATTAGATGGAGCCCCACAGGTCCAGGAACAAATAATCCCAATCAACAGCTGCTTTTGGCAAGCGCATCGTTTGATTCATCTATCAAGCTATGGGAAGTTGAGAAAGGACACCTTCTGTACAGCTTGGCTGGCCATAGGCAGCCAGTTTATTCTGTGGCCTTTAGCCCTGATGGCGAGTACCTGGCTAGTGGGTCGCTGGATCAAAGCCTACATATATGGTCCGTCAAAGAAGGCAGGATCCTGAAGACCTTCAGAGGGAGCGGTGGCATTTTTGAAGTGTGCTGGAACAAAGAAGGCAGCAAGATAGCAGCTTGCTTCTCCAACAATACTGTCTGCGTCATGGATTTCAGGATGTAG  >MH556920.1 Triticum aestivum WD40-repeat protein B (TaHOS15-B) mRNA, complete cds  ATGGGAGCGATAACCTCGGCGGAGCTCAACTTCCTCGTCTTCCGCTACCTACAGGAGTCCGGTTTTGTTCATGCCGCATTTACTTTAGGGTATGAAGCAGGGATACATAAAGGTGGGATAGATGGAAATGTTGTCCCTCCTGGCGCTCTTATCACTATCGTGCAGAAAGGCCTCCAGTACATAGAATTAGAAGCAAATACTGATGAAAATGATCAAGAAGTTGAGAAGGATTTTGCTCTTCTGGAACCTCTTGAAATCATCACAAAAGATGTTGAAGAGTTGCAACAGATTGTGAAGAAGAGAAAAAGGGAGAGGCTTCAAATTGATTCTGACAAGGACAAGGGGAAAGCGAAAGAGTGCATCGAGGAGCATGAACATCGTCTTGGTGGTGAACGGGAGAGAGAGCGCCATGACAAAGAAAAAGACCAAGAGATGGAAAAAGATAGAACTGAAAGAGACAAGGTGCAAGAGAAAGAGAAGGAAAGGGAAAAACAACACACAGAGCATATTGATAAGGTTAAGCACGAAGAGGATCCCCTTGCCAGTGGAGGTCCTAAACCAATGGATGTAAGTACAACTCATGAAATTTTTAGTGCGGATGTAACCGTTTTGGAAGGACACAGTTCAGAGGTTTTTGCTTGTGCATGGAGTCCAGCTGGTTCTCTTCTAGCTTCTGGGTCAGGAGACTCGACTGCTAGAATTTGGACAATTGCTGATGGTCCATGTGGTTCCATTCAATCCTCTCCTGCAAGTGTTCATGTTTTGAAACATTTTAAGGGTAGGACCAACGAGAAGAGCAAGGATGTCACCACACTTGACTGGAATGGGGAAGGAACACTATTGGCCACGGGCTCCTATGATGGCCAGGCAAGAATATGGAGTAGGGACGGAGAGTTAAAGCAAACTTTATTCAAACACAAGGGACCTATATTTTCTTTGAAATGGAATAGGAAAGGTGATTTTCTTCTAAGTGGCAGCGTAGACAAAACTGCTATTGTCTGGGATACAAAGACATGGGAGTGCAAGCAGCAATTTGAATTTCATTCAGCTCCAACACTAGATGTTGATTGGCGAAACAACAACTCTTTTGCAACATGCTCAACTGATAACATGATCTATGTTTGCAAGATTGGGGATCCACGCCCAGTTAAAACATTCAGTGGCCATCAAAGTGAAGTTAATGCTATCAAGTGGGATCCAACTGGTTCTTTGTTGGCTTCTTGTTCTGATGATTGGACTGCAAAGATATGGAGTGTGAAGCAGGACAAATGTGTATATGATTTTAAAGAGCATACCAAGGAAATATACACTATTAGATGGAGCCCCACAGGTCCAGGAACAAATAATCCCAATCAACAGCTGCTTTTGGCAAGCGCATCGTTTGATTCATCTATCAAGCTATGGGAAGTTGAGAAAGGACACCTTCTGTACAGCTTGGCTGGCCATAGGCAGCCAGTTTATTCTGTGGCCTTTAGCCCTGATGGTGAGTACCTGGCTAGTGGGTCGCTGGATCAAAGCCTACATATATGGTCCGTCAAAGAAGGCAGGATCCTGAAGACCTTCAGAGGGAGCGGTGGCATTTTTGAAGTGTGCTGGAACAAAGAAGGCAGCAAGATAGCAGCTTGCTTCTCCAACAATACTGTCTGCGTCATGGATTTCAGGATGTAG  >MH556921.1 Triticum aestivum WD40-repeat protein D (TaHOS15-D) mRNA, complete cds  ATGGGAGCGATAACCTCGGCGGAGCTCAACTTCCTCGTCTTCCGCTACCTACAGGAGTCCGGTTTTGTTCATGCTGCATTTACTTTAGGGTATGAAGCAGGGATACATAAAGGTGGGATAGATGGAAATGTTGTCCCTCCTGGCGCTCTTATCACTATCGTGCAGAAAGGCCTCCAGTACATAGAATTAGAAGCAAATACTGATGAAAATGATCAAGAAGTTGAGAAGGATTTTGCTCTTCTGGAACCTCTTGAAATCATCACAAAAGATGTTGAAGAGTTGCAACAGATTGTGAAGAAGAGAAAAAGGGAGAGGCTTCAAATTGATTGTGACAAGGACAAGGGGAAAGCGAAAGAGTGCATTGAGGAGCATGAACACCGTCTTGGTGGTGAACGGGAGAGAGAGCGCCATGACAAAGAAAAAGACCAAGAGATGGAAAGAGATAGAACTGAAAGAGACAAGGTGCAAGAGAAAGAGAAGGAAAGGGAAAAACAACACACAGAGCATATTGATAAGGTTAAGCATGAAGAGGATCCCCTTGCCAGTGGAGGTCCTAAACCAATGGATGTAAGTACAACTCCTCATGAAATTTTTAGTGCGGATGTAACCGTTTTGGAAGGACACAGTTCAGAGGTTTTTGCTTGTGCATGGAGTCCAGCTGGTTCTCTTCTAGCTTCTGGGTCAGGAGACTCAACTGCTAGAATTTGGACAATTCCTGATGGTCCATGTGGTTCCATTCAATCCTCTCCTGCAAGTGGTCATGTTTTGAAACATTTTAAGGGTAGGACCAACGAGAAGAGCAAGGATGTCACCACACTTGACTGGAATGGGGAAGGAACACTATTAGCCACGGGCTCCTATGATGGCCAAGCAAGAATATGGAGTAGGGACGGAGAGTTAAAGCAGACTTTATTCAAACACAAGGGACCTATATTTTCTTTAAAATGGAATAGGAAAGGTGATTTTCTTCTAAGTGGCAGCGTAGACAAAACTGCTATTGTCTGGGATACAAAGACATGGGAGTGCAAGCAGCAATTTGAATTTCATGTAGCTCCAACACTAGATGTTGATTGGCGAAACAACAACTCTTTTGCAACATGCTCAACTGATAACATGATCTATGTTTGCAAGATTGGGGATCCACGCCCAGTTAAAACATTCAGTGGCCATCAAAGTGAAGTTAATGCTATCAAGTGGGATCCAACTGGTTCTTTGTTGGCTTCTTGTTCTGATGATTGGACTGCAAAGATATGGAGTGTGAAGCAGGACAAATGTGTATATGATTTTAAAGAGCATACCAAGGAAATATACACTATTAGATGGAGCCCCACAGGTCCAGGAACAAATAATCCCAATCAACAGCTGCTTTTGGCAAGCGCATCGTTTGATTCATCTATCAAGCTATGGGAAGTTGAGAAAGGACACCTTCTGTACAGCTTGGCTGGCCATAGGCAGCCAGTTTATTCTGTGGCCTTTAGCCCTGATGGTGAGTACCTGGCTAGTGGGTCGCTGGATCAAAGCCTACATATATGGTCCGTCAAAGAAGGCAGGATCCTGAAGACCTTCAGAGGGAGCGGTGGCATTTTTGAAGTGTGCTGGAACAAAGAAGGCAGCAAGATAGCAGCTTGCTTCTCCAACAATACTGTCTGCGTCATGGATTTCAGGATGTAG |
| TaMDHAR4 (1448 bp) | >JX034702.1 Triticum aestivum cultivar Suwon 11 monodehydroascorbate reductase 4 mRNA (TaMDHAR4), complete cds  TCGGGATCCATGGGGCGCGCGTTCGTGTACGTCGTCCTGGGCGGCGGCGTGGCCGCGGGCTACGCGGCGCTCGAGTTCGCCCGCCGCGGCGGCTACTCCCGCGGCGAGCTCTGCATCATCTCCGAAGAGGCCGTTGCTCCTTATGAACGCCCTGCATTAAGCAAAGGCTATTTACTCCCAGAAGATCCTTCTCGTCTTCCAAAATTCCATACTTGTGTTGGAGCTAATGATGAGTTACTGACGACAAAATGGTACAAAGAACAAGGTATTGAACTTGTTCTTGGAACAAGAGTGATATCTGCTGATGTGAGACGGAAGACATTGCTTACAGCCACAGGAGAAACTATCAGCTATAAGACACTTATAATTGCAACAGGTGCTCGGGCTTTGAAGCTGGAAGAGTTTGGAATAAGCGGTTCAGATGCTGCAAACATATGTTATTTGCGCAATCTTGAAGACGCAGATAAATTAGTGAATGCGATGAGCTCATGTTCTGGTGGAAATGCTGTTGTCATCGGTGGTGGCTACATAGGAATGGAATGTGCAGCAGCATTGGTTACTAACAAGATAAAAGTAACCATGGTCTTCCCTGAAAAACACTGCATGGGTCGGCTATTTACAGAAAAAATTGCAGACTATTATGAAAGCTACTACACTTTGAAAGGAGTTACTTTTACCAAAGGAACTGTGCTTACATCCTTTGAAAAAGACTCAACAGGGAAGGTGACTTCGGTAATCTTAAGAGATGGTAACCACCTTCCTGCTGATATGGTAGTAGTTGGTATTGGGATCCGTGCAAATACCAGCCTTTTTGAAGGTCAGCTGCTGATGGAGAAAGGCGGCATAAAGGTAAACGGGCAAATGCAAACCAGCGACAGCTCCGTGTATGCTGTCGGTGATGTTGCCGCGTTCCCTATCAAGCTCTTTGACGGTGATATCCGACGGCTTGAGCATGTTGACTCGGCTCGTAGAACCGCCAGGCATGCTGTCGCGGCCATCCTGGAGCCTTCCAAAACCAGGGACGTCGATTACCTGCCATTCTTCTACTCCAGAGTCTTCACACTGTCCTGGCAGTTCTACGGAGACAATGTCGGAGAAGTAATTCACTACGGCGACTTCACGAGCAACAGCCCGAGGTTCGGCGCATACTGGGTCAGTAAGGGCCAGATCACGGGTGCGTTCCTGGAAGGTGGGAACCGAGACGACTACGAGGCGTTATCGATGGTTGTCCGGCGTAAAACCAAGGTCTTAGACATGCCTGAACTTGAAAGACAAGGCCTGGCGTTTGCCATCCAGGAAAGCAAGAAAGACGTGCCTGACAGCGGGGTTACCCTCGGCGAGAAGCCCACTTTCGTGTGGTATGCGACGGCGGGGGTTGTTGCGGCAGTGTCGATCTCCGCATTCGGTTATTGGTACGGCAGGAAGCGCCGCAGGTGGTGAGGTACCCC |
| TaSSI2 (1672 bp) | >AK332689.1 Triticum aestivum cDNA, clone: WT004_K01, cultivar: Chinese Spring (TaSSI2)  GGAGGTAGAGACAGACAACCAGGAGCACCCAAGAAGCGGTGCAGGAGGAATCCCGGCCTCTGTAGCAAACAAGAATCCATCCACCTGTCCTCCTCATCGAGATCTCGCACGGGGCGGCTCGTGGTGAGGTGAGTGAGTCGACCGGCCATGGCGTTCAGGGCCTGCCTCTCGTCCCACAAGGCCTCGCCGTCGCCTTCCGTCGCGCAGAGGAGGGCCAGCAATGGGGCACCGCCGGTGGTGGCCATGGCCTCCACCATGAACGAGGTCAAAATTGCCAAAAAGCCCTATGCTCCCCCACGCGAGGTGCATCTCCAAGTCATGCATTCGCTACCAGCACAAAAGCAGGAGATCTTTGATTCACTTCAATCTTGGGCTAGGGACAACCTATTGAACCTTCTGAAGCCAGTTGAGAAGTCATGGCAGCCACAGGACTTCCTACCAGAGCCTTCTTCCGAGGGATTTTATGATGAAGTAAAAGAACTGAGGGAGCGGGCAAAGGAAATCCCTGATGACTACTTTGTTTGCCTGGTTGGTGACATGGTTACTGAGGAAGCCCTTCCTACGTACCAAACAATGCTCAACACCCTTGATGGTGTCCGAGATGAAACTGGCGCAAGCCCAACTGCCTGGGCTGTTTGGACAAGAGCATGGACTGCTGAAGAAAACAGGCACGGTGATCTACTGAACAAGTACATGTACCTTTCCGGACGGGTTGACATGAGACAAATTGAGAAGACTATACAGTACCTTATTGGTTCTGGAATGGATCCAGGAACTGAGAACAATCCCTACATGGGTTTCCTTTACACATCATTCCAAGAGAGAGCTACTTTCATATCCCATGGCAATACTGCTAGGCATGCCAAGCAATTTGGGGACCTTAAACTGGCCCAGATATGTGGTACAATAGCAGCTGATGAGAAGCGCCATGAGACGGCTTACACCAAGATAGTTGAGAAGCTTTTTGAAATAGATCCTGACTACACGGTGCTTGCATTTGCTGACATGATGAGGAAGAAGATCTCGATGCCTGCTCATCTCATGTACGATGGTGAGGACGACAACCTATTTGAGCACTTCAGCTCGGTGGCGCAGCGGCTTGGGGTCTACACAGCGAAAGACTATGCCGACATCCTCGAGTTCTTGGTCCAGAGGTGGAAAGTTGCAGATCTCACTGGACTGTCCGGTGAAGGAAGACGGTCGCAGGACTATGTTTGCACTTTGGCAACGAGATTCAGGCGGCTGGACGAAAGAGCGCAGGCAAGGGCGAAACAGGGGCCTGTGGTTCCGTTCAGCTGGGTCTACGACCGCAAAGTGCAGCTCTAATAAAGAGGAAGGAACACAAGGTTAATGAGACTGCATCCATCCTATTATTTCGTGTTTTGGGTCAGTGTAGGTTTTCCTAATAGCAGGGTAGAATGTTTTATGTGCTGTCCTGTAAGTTGTTGGATGACAGTGGGGAAGAGGCTAGTGAAGGTGGTGTGAGATGCTGGCTGGCCATGAAGGGCTCCGTTGCAACCTTTTGTTGTGTTGAATATTTTATGTGACCGTTGAAGTTTACAAGGCTTGTATAAGTTTCAAAGTAATAAATGGATATTTGTATACAGACAGACCTATATGCTGGTGCACCATGAGATTCCAAACTTTAAAGCCAAAAAAAAAAAAAAAACGA |
| TaNAC032 (2492 bp) | >MT512636.1 Triticum aestivum NAC transcription factor 32-like protein gene (TaNAC032), complete cds  AAAAAATTCCGCAGTAATTATTTCTGTCTTATGTACGTACAGTAGGTACGAAAGCTGACGAAGAACCGTACGTAGGTTTGCTGTAGCGAAGTGACAGTATTGTTTAAGCCTGAGTAAAACTGTAGTTCCACATCAGTCACCAGCTGGCTATCGAGCAGCATCGATGCGTGCATAAACACAAAATCTGCCTTCGCACTGACATGTGAACGCTGAAAAGGGCGTAAATCATGAAGAGGTTTGGCCCATTTGGGAGTATTAATACGCGGCTGTCAAGCTCACGCGATGTCACGTTACGCGTAATCATCCGGTAGCTTGACGTGCCATCGGACGTCAGGGAGGCTAATCCAGCCGTGCGTCCACGATCGATCGGTCTGCGCCACGCGCGTCGCCGTCCAGGACTCGAGAGCCATCGCCATGCACGTCGCTCTCGCATAATCTCATTGCTTGGCTACCCTGACGACATGGGCACGTACGGTTACTTAGCCTAAGTAACTTACGCGTGTGCATGTATATATAGGCCATCCGCCAGCCGACTTCTTGGAGCGTTCCCAAATCAGGCCATCTCTTCTTCGTCGGCTCCGACTCCGAGCTAGTGCCGTGATGGAAGAAGGTTTTGTGTTCCGGGGGTGCGAGCTGCCGCCGGGGTTCCGGTTCCAGCCGACGGACCAGGAGATCATCGTCTGCTACCTCAAGAGGAAGGTCGCCTCCGCCGCCTCCGCCGTCACCTCCATCATCGCCGACGTCGACATCTACAAGTTCGACCCGTGGGAACTGCCCGGTAAGAATGCATGCATGCATACGTACGGCATACGTAGCCACGCCATGCAACCAGTGCAAAGTTTTGTTTCTATCTCTTTGACTCTTTCGTCCATGCACGAGCTTCCAAGAAAAATAAATACCGTTAGCTGCCGACCAGCTATCGACACCCATTTTTAATGTACATATCATCGTTCGGAACAAGACTGCAAGAGAGAAGTATTGTAATGCTGAATATACTGAGAAGTGGACGGAAGTTTGACAATTTTAATTTGTGCGTGCATGCAGACAAGGCGCAGTTCGGGGAGGGGGAGTGGTTCTTCTTCAGCCCGAGGGACCGCAAGTACCCCAACGGCGCGCGGCCCAACCGCACGGCGGGCTCGGGGTACTGGAAGGCCACCGGCACCGACAAGCCGATCCTGGCTGCCGGCGGCGCGCGCTGCCTCGGGGTCAAGAAGGCGCTCGTCTTCTACCAGGGCCGCTCCCCGCGGGGCACCAAGACTGAGTGGGTCATGCACGAGTACCGCCTCCTCCACGCCGACGCCGGCGCCGCCACCCGTCACAAGCCCCACGACTCCATGAGGGTGCGTACCAAACCATACGCGTAACTCGATGAATCTACGCACACACAGCTGGTTGTCGGTCAACTTAATTACATACTCTATTTCTTATGTTGGGTACAGCTGGACGACTGGGTCCTGTGCCGCGTCCGCAAGAAGGGCGTCGCCGTCGCGCCGGACATGGATGGCAACCCCGGAGCGCCGAGCCACGCGGAGGTTCAGGCAATTGATAGTACCACCGCAGCCGCGCACGGCGCCTTCGGCGACGACTGGACCGACGGCCAGCTCCTGCAGTACCTGATGAGCGGCGGATCCGGACAGGTCGACGGTGCCGGCGCCATCAGCGTGGCGGCGGGCCACGTCCATGACGGTGCGCGACGCGAGAGTGCGCCGGAGGTACATCTGGCGTCAGTGCTGGAGAACATCAAGAGGGACCTCTCGTTCCATGCCATGGACGACGTGTATTTCCTCCAGCCCAGCAAGCGGGCCAACTGCATGGGAGGAGGTGCGGGCCACCACACCGACGGCGACCAGCTGTCGCCGCCGACGTCCTTGTCCATGTTCGAGGACGATTAGTTAGGTGTTGCCTTGCGCACGAGGTATATGCATGCACGTAATCTTCAGTCGACCAGTGTGAGTAGAGTCACGAGTACTCGGGTAGCCACGACTGGGGATATAAACTGTCATCGATTTTATTTCCTCCACACTTGAGGAACAAATTAATTTCTCAATCAGTGCAGCTTCATGTGAACAGTCACGCATCTCTAGCAAATATCCTACTGGTCACACCTCATAACTCATATGGCTATCCAGTACTACTACTAATGTGGCACCTGCTCATCGCAAAATAATAATTTAAAATGGCACCTGGCATCACTTTTGAAACTCCTAATCATTCGAACACTTTTGGAAGCGAAAGTTCAAATCTGAACCCTTCGAGCACTTACCGAAAAAGGCTTTCGCCCCGCTTTATATATAAAGCACCAAACCACAAGCATCCAGTATAAACACACGCCACGACACCGCAACACACACACGCGCAGCAACACCACCCCTAGCACAACGGCCCCGAAAAAGGTGAGACTACATATGACGAACCATGCGTTCCGAGGTGGCACCTTCAGGAAGGATACGATATCGGAGCGCCGCTGTCGCCCGATCCAAGGATCAGAGTTT |
| TaULP5 (655 bp) | >KJ476506.1 Triticum aestivum cultivar Xingzi9104 ubiquitin-like protein 5 mRNA (TaULP5), complete cds  CTCTTCCAGAAAAAAAACAACTCCTTCGAGAGATTTCGCGGGGCGCAGGTTCGTGAGGAGCTAGGGTTTGGGAGGGAGCAGCGGAGAAGATGATCGAGGTGGTGCTGAACGACAGGCTGGGGAAGAAGGTGCGGGTGAAGTGCAACGAGGACGACACCATCGGCGACCTGAAGAAGCTCGTCGCGGCGCAGACGGGGACCAGGCCCGAGAAGATCCGCATCCAGAAGTGGTACACCATCTACAAGGACCACATCACCCTCGGCGACTACGAGATCCACGACGGCATGGGCCTCGAGCTCTACTACAACTAGAAGTATGGCCGCATTTGAGTCCCGTTGTCTTAAGAAGCTACTCCCTCCGTTCGGAATTACTTGTCTCGGATATGGATGTATCTAGAACTAAAATATGTTTAGATACATCCATTTCTACGACAAGTAATTCCGAACGGAGGGAGTACGTACTACTGTATATGGAATCCAACGAACCATAGAAGACTGCTGGATGTACCAATGACTGCCGACCTGAAATACTGAATGCTACTTATAATTGTGATGTGATGTGACGCTACTCGTACACTACATATAACATAATGTTTGTGACCTTTGTGCGATAACCGCTGTATTTTTAGTTATGGCAAGTTCGTTCTGCTTAAAAA |
| TaCBL4 (657 bp) | >KU736850.1 Triticum aestivum calcineurin B-like protein 4 (TaCBL4) mRNA, complete cds  ATGGGCTGCGTGTTGTCATCGCCGAGGCGGTCCAGGCGCACGCCGGGGTACGAGGAGCCCACCGTCCTCGCCTCCCAGACCTCCTTCACGGTGAACGAGGTGGAGGCGCTGTACGAGCTCTACAAGAAGCTCAGCTACTCCATCTTCAAGGACGGCCTCATCCACAAGGAGGAGTTCCGGCTGGCGCTGTTCAGGACCAGCAAAGGGGCGAACCTCTTCGCGGACAGGGTGTTCGACCTCTTCGATCTCAAGCGCAACGGGGTCATCGAGTTCGGCGAGTTCGTGCGCTCGCTCAGCATCTTCCACCCCAAAGCGCCTGAATCGGACAAGACCGCGTTTGCATTCAAGTTGTATGATTTGAGGGGGACAGGCTACATCGAGAAAGAAGAGCTCCGGGAGATGGTGGTGGCACTCCTCGACGAGTCCGACCTGTGCCTCTCCGACAGCGCCGTCGAGGAGATCGTCGACAATACGTTCAGTCAAGCAGACTCGAATGGCGACGACAGGATAGACCCCAAGGAGTGGGAGGAGTTCGTCAAGAAGAACCCGGCATCACTCAGGAACATGTCACTGCCCTATCTCCAGGACATTACGACGGCGTTTCCGAGCTTTGTGATGCATTCGGAAGTCGACGATTACAGCGGAATCAGCAAATAA |
| TaMDAR6 (1856 bp) | >KP201873.1 Triticum aestivum cultivar Suwon 11 chloroplast MDAR6 protein mRNA (TaMDAR6), complete cds; nuclear gene for chloroplast product  GAACGCACGAGGCCGCAGCTCCCCCCAAACCCTTCTGAGTTCTGATTCGCCGCCCGTTGGTCAGTTTTTTCCTTGGAGGTGGCTCTAGCTCTCCGGCAAAAGGCGGCGCAGATGGCTTCCGCGGCGGCGGCTGCGGCGGCGGGGACGGGGTGCTACTCGCAGACGTCCTGGGCGCTGCGACGCCTTGGCGCCGGAGGCGGCGCCCTCGCATCGGCGGCCACCCGCAGGAGGAACTGCTCCGTCGCGGCCGCCGTCTTTGACAACCAGAACCGCGAGTACGTGATCGTGGGCGGCGGGAACGCGGCGGGCTACGCGGCTAGGACGTTCGTCGAGCATGGCATGGCCGACGGCCGCCTCTGCATCGTCTCCAAAGAGGCTGTTCCACCATACGAGCGACCGGCACTGACCAAAGGCTATCTGTTTCCCCCAGAAAAAAAGCCGGCACGCCTACCTGGATTCCATACCTGTGTTGGATCTGGTGGCCAGAGGCAGACTGCCGAATGGTACAAGGAGAATGGCATAGAGGTGCTGTATGAGGATCCAGTTGAAGCATTTGATGGCAAAACACAGACCTTGAAAACTTCATCAGGGAAAATTCTGAAGTATGGGTCACTTATCATTTCTACTGGTTGTGCAGCTGCAAGACTACCTGAGAAAATTGGAGGAGACTTACCTGGAGTTCACTATATACGTGATGTTGCTGATGCTGATTCTCTAGTATCTTCATTGGGAAAAGCAAAGAAAATTGTTGTTATTGGTGGGGGCTATATTGGCATGGAGGTGGCTGCTGCAGCTTGTGGCTGGAATCTTGACACAACTATAATATTCCCAGAAGATCACATAATGCCAAGATTGTTTACGCCTTCCCTTGCTAAGAAGTATGAGGAACTGTATGAGCAAAATGGCGTCAAATTCGTAAAGGGAGCTCTTATTGACAAACTTGATGCTGGCTCTGATGGAAGGGTGTCTTCAGCTATACTTAAAGATGGTTCTGTTGTTGAAGCTGATACAGTTATTGTTGGTATAGGAGCAAAACCATCTGTCAGCCCCTTCGAAGCTGTGGGAGTTAACATCGAAGTTGGTGGAATAGAGGTTGATTCCATGTTTAGAACAAGTATACCTAGCATCTTTGCTATTGGAGATGTGGCAGCTTTTCCGCTCAAGATGTATGATAGAATAGCTCGAGTGGAGCATGTGGATCATGCCAGAGAGTCTGCACAACATTGTATAGAAACACTCGTAACATCCCAGGCAAAAGCGTATGACTACCTTCCGTATTTCTATTCTCGAGTTTTTGAGTATGAAGGAAGCTCCAGGAAAATTTGGTGGCAATTCTACGGAGATAACGTTGGTGAAACAATTGAAGTAGGGAACTTTGATCCTAAGATTGCTACCTTCTGGATTGACTCTGATAGTCGATTGAAGGGTGTTTTCCTTGAGAGTGGAACCTCAGAGGAATTCTCGCTTCTTCCAAAGCTCGCGAGATCACAGCCCATTGTCGACAAAGCTAAGCTCAAGAGTGCAACTTCAGTTGAAGATGCATTAGAGATTGCGAGAAGCTCTCTTTAGTCTGGCGCTTCTGTTTAATTAGTGGTCAACACATATGGCCTCTTCAGTTTGGGACGACAACATCTTCTTTTGAAAAGTCAGGACTAAGATGACAACTGATGCGGCCATAGTATACGTTCTATGGGACGTGTGTAATTTTCTTAGATGCCCCTCGGCTGCAAAGCACTACATCAACGTAGAGAGGTCTGCGGTGGAGTAATAACTGAGTCTGATCTGCTTCTTTCTTCCCAGCATCTCCTTGGTTGCTGTAATGTTGCTTCGTTTTGGTTGTAAAATATATGCGGTCC |
| TaEIL1 (2097 bp) | >KU030837.1 Triticum aestivum ethylene insensitive 3-like product (TaEIL1) mRNA, complete cds  CGGATAAAACTAGCAAGTGGGAGATCGACGCAGGTGAACACCACCACAGCTACAAGTAGAGGAAGTGGAATTTCTCTTGTGGAAAAAAGGGAGAGTTGCTTGAGTGGCCATGATGGGAGGTGGGCTGCTCATGGATCAGGGCATGGCGTTCTCCGGCGTGCACAACTTCGTGGATCTGCTCCAGCAGAACGGCGCCGACAAGAACCTCGGCTTCGGCTCCCTTATGCCGCAGACTTCCTCCGGCGACCAGTGCGTCATGGGCGAGGGCGACCTCGTGGACCCCCCGACGGACAACTTCCCGGACGCCGGGGAGGACGACAGCGACGACGATGTGGATGACATCGAGGAGCTGGAGCGCCGCATGTGGCGCGACCGCATGAAGCTCAAGCGCCTCAAGGAGCTGCAGCAGAGCCGCGGCAAAGAGCAGGCCGCCGCCGGCGGCGGCGTTGGCGACGGCTTGAAGCCGCGGCAGTCGCAGGAGCAGGCGCGCCGCAAGAAGATGTCGCGCGCGCAGGACGGCATCCTCAAGTACATGCTCAAGATGATGGAGGTGTGCCGCGCCCAGGGGTTCGTGTACGGCATCATTCCGGAGAAGGGCAAGCCCGTGAGCGGCGCCTCTGACAACCTCCGTGCCTGGTGGAAGGAGAAGGTCCGCTTCGACCGGAACGGCCCGGCCGCCATCGCCAAGTACCAGGCGGACAACGCCGTGCCGGGCTCCGAGAGCGAGCTGGCTTCCGGCACCGCCAGCCCGCACTCGCTGCAGGAGCTGCAGGACACCACGCTGGGCTCGCTCCTCTCGGCGCTCATGCAGCACTGCGATCCCCCGCAGCGAAGGTTCCCGCTCGAGAAGGGCATCTCTCCTCCATGGTGGCCGTCCGGCGACGAGGAGTGGTGGCCGGAGCTTGGCATCCCCAAGGACCAGGGCCCGCCCCCGTACAAGAAGCCCCATGACCTCAAGAAGGCTTGGAAGGTGAGCGTGCTCACCGCTGTCATCAAGCACATGTCGCCGGACATCGAGAAGATCCGGCGCCTCGTCCGCCAGTCCAAGTGCCTCCAGGACAAGATGACCGCCAAGGAGATCTCCACCTGGCTGGCCGTGGTGAAGCAGGAAGAGGAGCTGTTCATGAGGCTGCACCCGGGCGCTCGCCCTCCAGCGTCTGCCGGCGGCATCGCCAGTGCCATATCATTCAACGCCAGCTCGAGTGAGTACGACGTTGACCTCGCCGACGACTGCAAGGGCGATGAGGCCGGCACCCACAAGATGGCCATGGCCGATCCAACCGCCTTCAACCTCGGCGTGGCCATCCTGAATGACAAGTTCCTGATGCAAGCGCCCATGAAAGAGGAGACCGCCGACATGGAGTATGTCCAGAAGAGGAGCGCGGTGGCCGCCGAGCCGGAGCTGATGCTGAACAACCGCGTCTACACCTGCAACAACGTCCAGTGCCCGCACAGCGACTACGGGTACGGATTCCTTGACCGGAATGCGCGCAGCAGCCACCAGTACACCTGCAAGTACAATGATCCCCTCCCGCCAAGCGCGGAGAACAAGGCAGCGCCACCTGCGCCGCCGCAAGTCTTCCCGGCGGCCTACAACCAGCAGAACCATGGGCTCAACAACCTGGATTTTGGCCTGCCCATGGATGGGCAGAGGTCCATCGCCGAGCTGATGAACATGTACGACACCGCCTTCCCGGCCACCAACAAGAACATGGGCAACGACGACGTCACCATTATAGAGAGGCCCAATGCCATCACCCCGGGAGCGCAGATGGACGAGGGTTTCTTTGGACAGGGCAATGGAATTGGAGGCAATGGCGACAGTATGTTCAGTGATGTCAGTAACATGATGCAGCAGCAGCAGCAGCAGCAAGCACAACAGCCACAGCAGCAGCAGGCCCCGGCGCAGCAGCAGTTCTTCATCCGTGACGACGCGCAGGCGCAGTTCGGGAACCAGATGGGCAGCATCTCCGGCGCATCGGATTTCAGGTTCGGCTCTGGCTTCAACATGTCTGGCACCGTCGACTACCCGCAGAAGAACGACGGCCCCAATTGGTACTACTGAATGAAGAATAAACTCGTAGGGTCATCATAAGC |
| TaBln1 (573 bp) | >AK333112.1 Triticum aestivum cDNA, clone: WT005_K21, (TaBln1) cultivar: Chinese Spring  GACAAATTGTGAGCCAGGTCGCCAATCCAAGTAGAAACACAGCAAGGAAGCAAAGAGCCGACCGACCGAGGATGGCAAAGAACAACTCCTCTGCGACCTCGGTTCGGCTGATGCTCATCCTCCTCCTTCTGCTGGTTTTCGTTGGTGGCATCCTGGCAAATAGTGGGCCGTCCGAATGCAGCAACCCTGCGGCTCAGCGAGACTGCCCGCGGATCCCTAATGGCGGCTCATAAGCACGAGATGAACCATTTCCGCGCAGCAGGATTCGGTAGGATCTTGTGTTGGCAGGCGCTGGAAGCTTTCTATTATATATGCGAAACAAACAATATACATGTTCTTGAGGTGTTGGTTTTGTGATGTTTGTTCGTATTGATATGTTTGTACTGTGACCTGGTTTACATATAAATAATATGGCCTACCCCGTACATTAAAAAGTACTCTTCAGATCCTATATCTAAATAGGTGTCTACAATATCATTTTATTACCCAACAATGCCCTGTTATTATGTATCAGTATGATTTTTTTGGCCAGGTCCTAGACCCTGCCTAAAACCAAAAAAAAAAAAAAAACGA |
| TaADF4 (687 bp) | >KF246580.1 Triticum aestivum cultivar Suwon11 actin-depolymerizing factor 4 (TaADF4) mRNA, complete cds  AAGAGAAAGCATCAACCATGTCGAATTCCGCGTCAGGAATGGCCGTTTGCGACGAATGCAAACTCAAGTTCCAGGAACTCAAGGCGAAGAGGAGCTTCCGCTTCATCGTGTTCAAGATCAATGAGAAGGTGCAGCAGGTGGTGGTGGACAGGGTCGGGGAAAAAACCGAGAGCTATGATGATTTCACAGCCTGCTTGCCAGCTGACGAGTGCCGCTATGCAGTGTTTGATTTTGACTTCGTCACTGATGAGAACTGCCAGAAGAGCAAGATCTTCTTCATCTCTTGGGCTCCTGACACATCAAGGGTGAGGAGCAAGATGCTGTACGCGAGCTCCAAGGACCGCTTCAAGAGGGAGCTTGACGGCATCCAGGTGGAGCTACAGGCGACCGACCCGAGCGAGATGAGCATGGACATCGTAAAGGGGCGAGCCCTCTGAAGAAGAAAATCCGAGCAGCTACTTACTACGTACACCGACACCCAACCCAGGAGTAGGAGCAGAGGGGAGGCCCTCGTCTGCACCTGCTCCTGTTCTTGCAGTTCCTCCCCATCCATTTTCCCTGTCATTCTTTTCCTGGAAGCAAAGTGTTGTGTTCTGTATGAGACTGTATGCCGTCTTCATTCTTGTTATCCCCCGTGTTTGGTGCCATAGTCTCTGACTACTGCTGTAAACTTAGAACATATTTGCA |
| TaRAR1 (3512 bp, 5083 bp, 3486 bp) | >KJ907388.1 Triticum aestivum RAR1 (RAR1) gene, TaRAR1-A1 allele, complete cds  ATGTCGGCGGAGACGGAGACGAGCGCCGCCGCGCCGGCGCCCGCGCCCATGCGGTGCCAGCGCATAGGCTGCGACGCCATGTTCACTGACGACGACAACCCCGACGGCTCCTGCCACTACCACCCCTCCGGAAGCTCCCTCTTCCCCATCCCCTCGCTCGAAAAAATTGCATCGTCTCGCTCCTTCGAGCCCCTGTAGATCATGGAGGTGGAAGCCTTCCTTGTAGCCCCCAAGCTCGCTTCTCGGATCTCGCTTTGGTACTCGTATGCTGTTAGGATCTGGGTGTAGTCGGGTGTCGAAGGTGTAGATCCTCTACGCCCACGGATGGCACCAATCGGGATAGCTGCTGTGTCGCAGAAATTGTAATCTACCCAAATCTTGGAGACAGTCAACTAAGCATGTACTCTTATTATGTTGTTGGCATCAACATTGAATCGACCCATATGGTGGTAAATTGGTAGCATGTTCAAGCTAATCGCATTATTCAGTCAGCGAGAAGCGGCGGTTGATTTCATTGACGAAATAGGCTACAAGTAAATGCAGGCGCACTTTTGGGGAACATTGTTTTGTGGCTTGCAGACAGACCTGGAAGTTCCCTGTTGACGACCATGTTATATTATGTTTTTTTAGAGTTTGGTCACCTGCTTATTTATTTTAGCCTTTTAGGACATATAAAAACTTGGCACCCTAAACAAAACAAAAATGGGAAGGGGTGAAGGGACCTGTGCTTGGTTTGATGTTATGATTGAAGTGTGATCTTGCATTTATGTTCTACTCCCTCCGGTCCTTTTTAGTTTGTATATAAGATTTGTCCGAAGTCAAAGTATCTTTACTTTGACCAAACTTATAGAAAACAGTATCAACATTAACAATACCAAATCAATAATGTTAGATTCATTATGAAATGTAGTTTCATAGTATATATAGTTGGTACTGTACATGTTTATATTTTTTAATATAAATTTGGTCAAACTTGGTTGAGTTTGACTTGACAGAAATCTAATACGCGGGGTAAAAAGGACCGGAGGGAGTACAAAGCAATCTGCTAGGAAAGAGTGAATTCCAGAAAGATACAAGTTTAATATAGGATCTATCACTAAGCTACAGATTGAAGAACAGATATTACAACTACAGATTCGAGCAAACACCATGTAGTTTTGTGGTGTAATTGCCAAATTGGTCACTAAATCTGCAGTTACTTGAAGTTGGTCAAATCTGTAGCTTTATTATACGGATGGTCCATAAATCTGTGTGTGAAGTTTCTTTAAATACGTAATTTTGAACATGTAGTTTTGCTAAATTTACTGCAGGAAAATAGCAATTTAAACACTTATTTCACTGTTACTCTAGTTATTCTGCAATGTTTGATACCAATGCATTAAGTAACTGGTGCCAAGGAAGCTTTAAGTAATTCTCTTTTTGAAGTTGGTCAAATCTTTAGTTGTATTATACAATGGGTCCATACATCTGCGTGTGAAGTTTATTTAAATTCGTAATTTCGTGATAGTTTCTACACTGAACATGTACAACAACAACAACAACAAAACCTTTAGTCCCAAACAAGTTGGGGTAGGCTAGAGGTGAAACCCATAAGATCTCGCAACCAACTCATGGCTCTGGCACATGGATAGCAAGCTTCCACGCACCCCTGTCCATAGCTAGCTCTTTGTCGATACTCCAATCCTTCAGGTCTCTCTTAACGGACTCCTCCCATGTCAAAATCGGTCGACCCCGCCCTCTCTTGACATTCTCCGCACGCTTTAGCCGTCCGCTATGCACTGGAGCTTCTGGAGGCCTGCGCTGAATATGCCCAAACCATCTCAAACGATGTTGGACAAGCTTCTCCTCAATTGGTGCTACCCCAACTCTATCTCGTATATCATCATTCCGGACTCGATCCTTCCTCGTGTGGCCACACATCCATCTCAACATACGCATCTCCGCCACACCTAACTGTTGAACATGCCGCCTTTTAGTCGGCCAACACTCCGCGCCATACAACATTGCGGGTCGAACCGCCGTCCTGTAGAACTTGCCTTTTAGCTTTTGTGGCACTCTCTTGTCATAGAGAATGCCAGAAAGCTTGGCGCCACTTCATCCATCCGGCTTTGATTCGATGGTTCACATCTTCATCAATACCCCCATCCTCCTGCAACATTGACCCCAAATACCGAAAGGTGTCCTTCCGAGGTACCACCTGGCCATCAAGGCTAACCTCCTCCTCCTCACAGCTAGTAGTACTGAAACCGCACATCATGTACTCGGTTTTAGTTCTACTAAGCCTAAACCCTTTCAATTCCAAGGTTTGTCTCCATAACTCTAACTTCCTATTTACCCCCGTCCGACTATCGTCAACTAGCACCACATCATCCGCAAAGGGCATACACCATGGGATATCTCCTTGTATACCCCTTGTGACCTCATCCATCACCAATGCAAAAAGATAAGGGCTCAAAGCTGACCCCTGATGCAGTCCTATCTTAACCGGGAAGTCATCGGTGTCGACATCACTTGTTCGAACACTTGTCACAACATTATTGTACATGTCCTTGATGAGGGTAATGTACTTTGCTGGGACTTTGTGTTTCTCCAAGGCCCACCACATGACATTCTGCGGTATCTTATCATAGGCCTTCTCCAAGTCAATGAACACCATATGCAAGTCCTTCTTATGCTCCCTATATCTCTCCATAAGTTGTCGTACCAAGAAAATGGCTTCCATGGTCGACCTCCCAGGCATGAAACCAAACTGATTTTTGGTCACGCTTGTCATTCTTCTTAAGCGGTGCTCAATGACTCTCTCCCATAGCGTGTAGTTGTACGAAATTTACTCCAGGAAAAAATAGCTATTTAAACACCTATTTCACTGATGCAGATGTTTGATACCAATACAAGAAGTAACTGGCACTAAGGAATCTTCATATAATTCTGTTTTTGTCTTTTCAAAAAATACGAGAACTGCTCAACAATAAGCACTACTCTGTAGGCAATCCCAAATTCGATTGTGATATTCACCAGTTGAACTACTGTATTTGATCTTAAACATTTTTGAAAGTACACTAATAAGGCGCGTTCAGGATACCAGTTATAGATCTGAGATTGAATAGTTACATATTGGAAAAATAAGTTATGAATCAATTAGGACATGATAATAACTTAGCATGCATTTCTATCTTTCTGACCAGGGAAAATCCTTTCCTTTTTGTTTTTGTGAAGTGTTTCAGAAAATTACAAATTTACAGCATCCATTTCCATATTTTTAGCTATTAACAGCTCTCCCCCTTTTTTGTTTTGTTGCAGGGAGTAAGTTCGTTTGCTACACTTGTGGGATTATTTCTGTTTTTGATAAACCATAGGTTCTTTAATATATTATTTTCAGCCTATGTTTCATGATGGCATGAAAGAGTGGAGCTGTTGCAAGCAAAGAAGCCATGATTTTAGCTTATTTTTGGCTATTCCTGGGTATGGATCCTCTCTCCTCCCATGACAGGCAGTGGCAGAATAGTTACGTATTTCTTGTTATGCATGGTGTCATAGATACAAATTATGTTTGCAGATAATCTAAATTATTAGCGAGTCCAGCTCTCTATGCATTGCAATTAGAAAGTTAGATTTGCACCACCTATGTCTGATTTGTGATGGAACAGATGGGCGTTGGAGATTTCTTTGGTCACCGATTAGGGATTAGCACTATGATCAACCATTCTCATTTCCTTCTTCATTTATTGCCTAGGACTCAGACTTGTCTAATTTATAGCTTGATTGAACGTGGGGGGTCACTTGTCAACATACCTTTTTAAATCCACGTCGTTTGTTTCTGTAAGGTACTACTACTTGTCTGATAAGGAGAACCCTGTATGCATTTATTTGGATTCACTCGAAGTCTAGTACAGTTTTCTTCTTGATGTCATGTTATAATTGGTCTTTGGCAAGTACTGTAATTTAACTTGTTTGGCCCAGTCAATACCATTGCGCTACAGCGCTACATGGCCGAATAAACATATGCTTGCAGTTCAGAGTTTAGACTGAACAGGTTCTCAGTTATCTTGTTGGTTCATTTTACTGAGTAAGCGCAGCATAATGTACAGAAGATATTTGTATGTTAGTGCTTTGGCTACTTCACCATCCTCTATTAGATCAGCTTGAATTGGCTAAAGAAAGTTATTCCACTTTTCAGGGAGCTCCAGTGATATGGCTCCAATTTGCTGGCAAATAAACCTGAAAACGATGCCTTATGTCTTGGTTCTTACAGTACTATTTTTGTAATTCAATGTTCCAGCTTTAACCATTGCCTCCTCTCATTCTAGACCAGACCGTCCTTGCTTTAAATATCAACACCCATGACCTGTTATATTTGGGTAAACTAGACATAACTGACCGCATTCTCTACCGTGGTGTAGATGCGCCACAGGGAAGCATACAACTGAGAAACCAGTCACAAAAGCTGTTTCTCTTAACTCAAAGGCAACCCCACCAAAGTTAGCTCCAATCCAGTCTTCTAAGCAGGGTGTGGAAACCGAGGCCTGCTCCAGGTGCCGTCAGGGTTTCTTTTGCTCCGACCATGGTGAGTTTGTGTCTTTGAACTCTTTGCTTCAATGTGACAATGCATAACTTCAAAAAATTGGTTGATGTTTTCTCTTTTGTTGAAAAATTTAGTGTATGAACCATTCATCAATGTATTTTTAGGATCACAGCCGAAGGCACAAAAACCAGTTGCTGTAAATGGTACAAATACGGAACCTGTCGAAAAATGCTCAGTTCCACAGCCCAGGAAAAAAGTTGTTAATATAAATGAGCCTAGGGTTTGTAAGAATAAAGGATGTGGTAAAACCTACAAGGAGAAGGATAACCATGATGCTGCATGTGAATACCATCCAGGTCCTGCGGTTTTCCATGACAGGAATAGAGGGGTACGTATTTTCTCTCTAATTAAATTTGTGTTCATTTATGTTCACCCTGATTCTTACCTCCATGTATTTTCTCTTTGCAGTGGAAGTGTTGCGATATCCACGTCAAGGAGTTTGACGAATTTATGGAGATACCTCCGTGCACAAAGGGGTGGCACAATGCCGATGCTGTGTGA  >KJ907389.1 Triticum aestivum RAR1 (RAR1) gene, TaRAR1-B1 allele, complete cds  ATGTCGGCGGAGACGGAGAAGAGCGCCGCCGCGCCCGCGCCCGCGCCCATGCGGTGCCAGCGAATAGGCTGCGACGCCATGTTCACCGACGACGACAACCCCGACGGCTCCTGCCACTACCACCCCTCCGGAAGCTCACTCCCCCTCCCCCCACTCGAAAAAATCGCATCGTTTCGCTCCCTCGACCCCCTGTAGATCATGGAGGTGCATGTCTTCCTTGTAGCCCCTAAGCTCGCTTCTCGGATCTCGCTTTGGTTAGACTCATATGCTAAGGATCTGGGTGTAGTTTGGGAGTCGAGGGTTTAGATCCTCTACGTCCACGGAAAGCGCGAATTGGGATAGCTGCTCTGTCGTAGAAAGTGTAATCTACCCAAATCTTGGAGACAGTCAACTAAGCATGCACTCTTATTATGTTGTTGGCATCAACATTGATTCAATCGATATGGCGGTAAATTGGCAGCATGTTCCCGCTAATCGCATTATTCAGTCAGTGAGAAGCGGTGGTTGATTGCATTGACGAAATAGGCTACAAATAAATGCAGGCGCACTTTTGGGGAACATTGTTTTGTGGGTTGTAGACAGACCTGGAAGTTCCCTGTTGATGAGGCCTTGTGCGCAATATGTGTGACGACCATGTTATATTATGTTTGTTTAGAGTTCGGTCACGTGCTCATTTATTTTAGCCTTTTAGGACATAAAAACTTGGCACCCTAAACAAAACAAAAATGGAAAGGGGTGAAGGGACCTTTGCTTGGTTTGATGTTATGATTGAAGTGTGATCTTGCATTTATGCTCTACAAAGCAATTTGCTAGGAAAGAGTGAATTCCAGAAAGCTATAAGTTTAATATACGATCTGTCACTAAGCTACAGTTTGAAGATCAAATATACAACTACAGATTCGAGCAAACATCATGTAGTTTTGTGGTGTAATTGCCAAATTGGTCTCTAAATCTGCAGTTCTTTGAAGTTGGTCAAATCTTTAGCTTTATTTTACAATTGGTCCATAAATCTGTGTGTGAAGTTTCTTTAAATACGTAATTTTGAACATGTAGTTTTGCTAAATTTACTGCAGGAAAATAGCAATTTTAAACACTTGTTTCACCGTTACTCTAGTTATTCTGCAATGTTTGATACCAATGCATTAAGTAACTGGCGCCAAAGGAATCTTTAAGTAATTCTCCTTTTGAAGTTGGTCAAATCTTTAGTTGTATTATACAATGGATCCATACATCTGCGTGTGAAGTTTATTTAAATTCGTAATTTCGTGATAGTTTCTACACTGAACATGTAGTTGTACGAAATTTACTCCAGGAAAAATAGCTATTTAAACGCCTATTTCACTGATGCAGATGCTTGATACCAACACAAGAAGTAACTGGCACCAAGGAATCTTCATATAATTCTGTTTTTGTCTTTTGAAAAAATACGAGAACTGCTCAACAATAAGCACTACTCTGTAGGCAATCCCAAGTTCGATTGTGATATTCACCAGTTGAACTACTGTAATTGATCTTAAACATTTTCAAAAGTACACTAATAAGACGCGTTCAGGATATCAGTTATAGATATGAGATTGAATAGTTAAATATTGGAAAAATAAGTTATGAATCAATTAGCTTTTCTTTTGTTTTCGTGAAGTGTTTCACTGTTTCAGAAAATTACAAATTTACAGCATCCATTTCCATATTTTTAGCTATTAACAGCTCTCCCCCTTTTTTGTTTTGTTGCAGGGAGTAAGTTCGTTTGCTGCCCTTGTGGGATTATTTCTGTTTTTGATAAAACCATAGGTTCTCTAATATATTATTTTCAGCCTATGTTTCATGATGGCATGAAAGAGTGGAGCTGTTGCAAGCAAAGAAGCCATGATTTTAGCTTATTTTTAGCTATTCCTGGGTAAGGATCCTCTCTCCTCCCATGACAGCCAGTGGCAGAATAGTTACGTATGTCTTGTTATGGTGTCATAGATACAAATTATGTTTGCAAATAATCTAAATTATTAGCGAGTCCAGCTCTCCATGCATTGCAATTAGAAAGTTAGATTTGCACCATCTATGGCTGATTTGTGATGGAACAGATGGGTGTTTGAGATTTCTTTGGTCACCGACTAGGAATTAGCACTATGATCAACCATTCTCATTTCCTTCTTCATTTGTTGCCTAGGACTTAGACTTGTCTAATTTATAGCTTGATTGAACGTAGGGGGTCACTTGTCAACATACCTTTTTAAATCCACGTTATTTGTTTCTGTAAGGTGCTACTACTGTCTGATAAGGAGAACCCTGTATGCACTTATTTGGATTCACTCGAAGTCTAGTGCAGTTTCCTTCTTAATGTCATGTTATAATGGGGTCTTTGCTAAGTACTGTAATTTAACTTGTTTGGCCCAGTCAATATCATTGCGCTACATCGCTACATGGCCGAATAAACATATACTTGCAGTTCAGAGTTTAGACTGAACAGGTTCTCAGTTATCTTATTGGTTCATTTTACTGATTAAGCGCAGCATAATGTACAGAAGATATTTGTATGTTAGTGCTTTGGCTACTTCACCACCCTCTATTAAATCACCTTGAATTGCCTAAAGAAAGTTATTCCACTTTTCAGGGAGCTCCAGTGATATGGCTCCAATTTGCTGGCAAATAAACCTGAAAACGATGCCTTATGTCTCGGTTCTTACAGTACTATTTTTATAACGTAATCAATGTTCCAGCTTTAACCATTGCCTTCTCTCATTCTAGACCAGACCGTCCTTGCTTTAAATACCAACACCCATGACCTGTTATATTTAGGGAAACTAGACATAACTGACCGCATTCTCTACCGTGGTGTAGATGTGCCACAGGGAAGCATACAACTGAGAAACCAGTCACAAAAGCTGTTTCTCTTAACTCAAAGGCAACCCCACCAAAGTTAGCTCCAGTCCAGTCTTCTAAGCAGGGTGTGGAAACCGAGGCCTGCTCCAGGTGCCGTCAGGGTTTCTTTTGCTCCGACCATGGTGAGTTTGTGTCTTTGAACTCTTCACTTCAATGTGACTATGCATAACGTCAAAAAATTGGTTGATTTTTTTTCTTTTGTTGAAAAATTTAGTTTATGAACCATTCATCAATGTATTTTTAGGATCACAGCCCAAGGCACAAAAACCAGTTGCTGTAAATGGTACAAATACGGAACCTGTCGAGAAATGCTCGGTTCCACAGCCCAAGAAAAAGGTTGTTAATATAAACGAGCCTAGGGTTTGTAAGAATAAAGGATGTGGTAAAACCTACAAGGAGAAGGATAACCATGATGCTGCATGCGAATACCATCCAGGCCCTGCGGTTTTCCATGACAGGAATAGAGGGGTATGTATTTTCTCTCGTTAAATTCGTGTTCATGTATATTCACCCTGATTCTTACCTCATGTGTTTTTCTCTTGCAGTGGAAGTGTTGCGATGTCCATGTCAAGGAGTTTGACGAATTTATGGAGATACCTCCATGCACAAAGGGGTGGCACAATGCTGATGCCGTGTGA  >KJ907390.1 Triticum aestivum RAR1 (RAR1) gene, TaRAR1-D1 allele, complete cds  ATGTCGGCGGAGACGGAGAAGAGCGCCGCCGCGCCGGCGCCCGCGCCCGCGCCCATGCGGTGCCAGCGAATAGGCTGTGACGCCATGTTCACCGACGACGACAACCCCGACGGCTCCTGCCACTACCACCCCTCCGGAAGCTCCCTCCTCCCCCGTCCCCTCCCACTCGAAAAAATCGCATCGTTTCGCTCCTTCGAGCTCCTGTAGATCATGGAGGTGGAAGCCTTCCTTGTAACCCCTAAGCTCGCTTCTCGGATCTCACTTTGGTACTCGTATGCTATAAGGATCTGGGTGTAGTCGGGAATCGAGGGTTTAGATCCTCTACGTCCACGGAAGGCACGAATTGGGGTAGCTGCTGTGTAGTAGAAAATGCAATCTACCCAAACCTTGGGAGACAAGTCTTAAGAGACGACTTGGGTAACAAACTAAGCATATACTCTTATTATGTTGCTGGCATCAGTATCCATTCGATCCATATGGTGGTAGATTGGTAGCATGTTCACTCTAATCGCGTTATTCAGTCAGTCAGAAGCGGCGGTTGATTGCATTGACAAAATGGGCTACAAGTAAACGCGGGCACACTTTTGGGGAACATTGTTTTGTGGGTTACAGACAGACCTGGAAGTTGCCTGTTGATGAGGCCTTGTGTGCAATATGTGTGACGACCATGTTATATTATGTCTATTTAGAGTTCGGTCACGTACTCATTTATTTTAGGACATAAAAACTTGGCACGCTAAACAAAACAAAAATGCAAAGGGGTGAAGGGACCTTTGCTTGGTTTGATGTTATGATTGAAGTGTGATCTTGCATTGATGTTCTACAAAGCGATTTGCTAGGAAAGAGTGAATTCCAGGAAGCTACAAGTTTAATATACGATCTATCACTAAGCTACAGATTGAAGAACAAATATTACAACTACAGATTCGAGCAAACACCATGTAGTTTTGTGGTGTAATTGCCAAATTGGTCACTAAATCTGCAGTTCTTTGAAGTTGGTCAAATCTGTAGCTTTATCATACAATTGGTCCATAAATCTGTGTGTGAAGTTTCTTTAAATACGTAATTTTGAACATGTAGTTTTGCTAAATTTACTGCAGGAAAAATAGCAATTTAAACACTCATTTCACTGTTACTCTATTTATTCTGCAATGTTTGGTACCAATGCATTAAGTAACTGGTGCCAAGGAATCTTTAAGTAATTCTCTTTTTGAAGTTGGTCAAATCTTTAGTTGTATTATACAATGGGTCTATACATCTGCGTGTGAAGTTTATTTAAAATCGTAATTTCGTGATAGATTCTACACTGAACATGTAGTTGTACGAAATTTACTCCAGGACAAAATAGCTATTTAAACACCTATTCCACTGATGCAGATGTTTGATACCAATACAAGAAGTAACTGGCATAAAGGAATCTTCATATAATTCTGTTATTGTCTTTTCGAAAAATACGAGAACTGCTCAACAATAAGCACTACTCTGTAGGCAATCCCCAATTTGATTGTGATTGAATAGTTAAATATTGGAAAAAATAAGGTATGAATCAATTAGGACATGATAATAACTTAGCATGCATTTCTATCTTTCTGACCAGGGAAAATCCTTCTTTTTTCATGAAGTGTTTCAGAAAATTACAAATTTACAGCATCCATTTCCATATTTTAGCTATTAACAGCTCTCCCCCTTTTTTGTTTTGTTGCAGGGAGTAAGTTCGTTTGCTGCACTTGTGGGATTATTTCTGTTTTTGATAAAACCATAGGTTCTCTAATATATTATTTTCAGCCTATGTTTCATGATGGCATGAAAGAGTGGAGCTGTTGCAAGCAAAGAAGCCATGATTTTAGCTTATTTTTGGCTATTCCTGGGTAAGGATCCTCTCTCCTCCCATGACAGGCAGTGGCAGAATAGTAGTTATGTATTTCTTGTTATGCATGGTGTCATAGATACAGATTATGTTTGCAAATAATCTAAATTATTAGCGAGTCCAGTTCTCTATGCATTGCAATTAGAAAGTTAGATTTGCACCATCTATGGCTGATTTGTGATGGAACAGATGGGCGTTTGAGATTTCGTTGGTCACCGATTAGGAATTAGCACTATGATCAACCATTCTCATTTCCTTCATTTGTTGCCTAGGACTTAGACTTGTCTAATTTATAGCTTGATTGAACGTGGGGGGTCACTTGTCAACATACCTTTTTAAATCCAGGTTGTTTGTTTCTGTAAGGTACTACTACTGTCTGATAAGGAGAACCCTGTATGCATTTATTTTGATTATTCGAAGTCTAGTACAGTTTCCTTCTTAATGTCATGTTATAATGGGTCTTTGGTAAGTACTGTAATTTAACTTGTTTGGCCCAGTCAATATCATTGCGCTACATCGCTACATGGCCGAATAAACATATGCTTGCAGTTCAGAGTTTAGACTGAACAGGTTCTCAGTTATCTTATTGGTTCATTTTACTGATTAAGCGCAGCATAATGTACAGAAGATATTTGTATGTTAGTGCTTTGGCTACTTCACCACCCTCTATTAAATCAGCTTGAATTGGCTAAAGAAAGTTATTCCACTTTTCAGGGAGCTCCAGTGATATGGCTCCAATTTGCTGGCAAATAAACCTGAAAACGATGCCTTATGTCTCGGTTCTTACAGTACTATTTTTGTAACGTAATCAATGTTCCAGCTTTAACCATTGCCTTCTCTCATTCTAGAGCAGACCGTCCTTGCTTTAAGTATCAACACCCATGACCTGTTATATTTAGGGAAACTAGACATAACTGACCGCATTCTCTACCGTGGTGTAGATGTGCCACAGGGAAGCATACAACTGAGAAACCAGTCACAAAAGCTGTTTCTCTTAACTCAAAGGCAACCCCACCAAAGTTAGCTCCAATCCAGTCTTCTAAGCAGGGTGTGGAAACCGAGGCCTGCTCCAGGTGCCGTCAGGGTTTCTTTTGCTCCGACCATGGTGAGTTTTGTGTCTTTGAACTCTCAATGTGACAATGCATAACTTCAAAAAATTGGTTGATTTTTTTTCTTTTGTTGAAAAATTTAGTGTATGAACCATTCATCAATGTATTTTTAGGATCACAGCCCAAGGCACAAAAACCAGTTGCTGTAAATGGTACAAATACGGAACCTGTCGAAAAATGCTCAGTTCCACAGCCCAAGAAAAAAGTTGTTAATATAAATGAGCCTAGGGTTTGTAAGAATAAAGGATGTGGTAAAACCTACAAAGAGAAGGATAACCATGATGCTGCATGTGAATACCATCCAGGTCCTGCGGTTTTCCATGACAGGAATAGAGGGGTACGTATTTTCCCTCTAATTAAATTCATGTTCATGTATGTTCACCCTGATTCTTACCTCCCATGTATTTTCTCTTGCAGTGGAAGTGTTGCGATGTCCACGTCAAGGAGTTTGACGAATTTATGGAGATACCTCCATGCACAAAGGGCTGGCACAATGCTGATGCCGTATGA |
